# Diversifying the structure of zinc finger nucleases for high-precision genome editing

**DOI:** 10.1038/s41467-019-08867-x

**Published:** 2019-03-08

**Authors:** David E. Paschon, Stephanie Lussier, Tenzin Wangzor, Danny F. Xia, Patrick W. Li, Sarah J. Hinkley, Nicholas A. Scarlott, Stephen C. Lam, Adam J. Waite, Lynn N. Truong, Nimisha Gandhi, Bhakti N. Kadam, Deepak P. Patil, David A. Shivak, Gary K. Lee, Michael C. Holmes, Lei Zhang, Jeffrey C. Miller, Edward J. Rebar

**Affiliations:** Sangamo Therapeutics, Inc., 501 Canal Boulevard, Suite A100, Richmond, California 94804 USA

## Abstract

Genome editing for therapeutic applications often requires cleavage within a narrow sequence window. Here, to enable such high-precision targeting with zinc-finger nucleases (ZFNs), we have developed an expanded set of architectures that collectively increase the configurational options available for design by a factor of 64. These new architectures feature the functional attachment of the FokI cleavage domain to the amino terminus of one or both zinc-finger proteins (ZFPs) in the ZFN dimer, as well as the option to skip bases between the target triplets of otherwise adjacent fingers in each zinc-finger array. Using our new architectures, we demonstrate targeting of an arbitrarily chosen 28 bp genomic locus at a density that approaches 1.0 (i.e., efficient ZFNs available for targeting almost every base step). We show that these new architectures may be used for targeting three loci of therapeutic significance with a high degree of precision, efficiency, and specificity.

## Abstract (web summary)

Genome editing often requires cleavage within a narrow sequence window. Here the authors develop an expanded set of zinc finger nuclease architectures that increase the available configurations by a factor of 64 and can target almost every base at loci of therapeutic significance.

## Introduction

As the archetypal platform for programmable DNA cleavage^1^, zinc-finger nucleases (ZFNs) have had a central role in the development and application of genome engineering technologies. From initial demonstrations of efficient gene editing in higher eukaryotes^2–4^ to current applications in the engineering of hematopoietic stem cells (HSCs)^5–10^ and tumor-targeted T-cells^11,12^ ZFNs have provided key, targeted cleavage events used to establish new genome engineering concepts and to extend the reach of this technology into wider spheres of biological research. Key milestones have included the first editing of an endogenous human locus^13^, first demonstration of in vivo editing^14^, and the first demonstration that engineered cells^15,16^ and entire organisms^17^ could be derived that exhibit no evidence of off-target cleavage. In recent years, ZFNs have been increasingly developed for therapeutic applications with protocols for engineering HIV-resistant T-cells^18^, restoring productive erythropoiesis to B-thalassemic HSC^19^, and editing gene targets in situ^20,21^ (ClinicalTrials.gov identifier: NCT03041324) having reached the clinic.

**Fig. 1:**
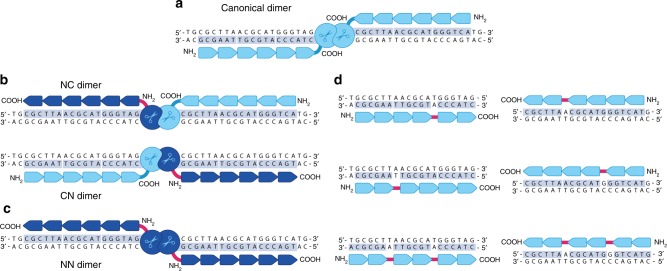
Linkers and architectures developed in this study. **a** Sketch of the canonical ZFN dimer architecture. Circles marked with a scissors symbol denote the FokI cleavage domain. A tandem array of six arrows indicates each designed six-finger ZFP. Key features of this architecture include attachment of the FokI nuclease domain to the carboxy terminus of each zinc finger array and a lack of base-skipping between adjacent zinc fingers. ZFNs are shown interacting with duplex DNA, with black text on a gray background denoting ZFN target sites. **b** Alternative architectures enabled via pairing of ZFNs bearing an amino-terminal FokI cleavage domain (dark blue) and a carboxy-terminal FokI cleavage domain (light blue). The linker joining the FokI nuclease domain to the amino terminus of the ZFP is shown in red. ZFNs bearing an amino-terminal FokI attachment are able to recognize a target on the opposite DNA strand, relative to their canonical counterparts (compare with **a**). Thus, these architectures allow both ZFNs to recognize the same DNA strand. These two architectures are structurally identical, although for this study they will be referred to as NC and CN dimers denoting the FokI attachment point for the upstream and downstream ZFN, respectively. **c** ZFN architecture enabled via pairing two ZFNs with amino-terminal FokI nuclease domain fusions. This architecture is the inverse of the canonical pair shown in **a** and is referred to as an NN dimer. **d** Recognition of alternative DNA frames and sequences enabled by insertion of base-skipping linkers between fingers 2 and 3 or 4 and 5 of a six-finger ZFP. Skipped bases are shown without a gray background. The skipping linker is shown as a red bar between fingers

ZFNs exhibit several features that make them especially suitable for therapeutic applications, including a compact size compatible with AAV vectors^22^ and an all-protein structure that enables access to every genome compartment, including mitochondrial DNA^23^. ZFNs also feature an especially versatile DNA-binding interface that can be adapted to distinguish epigenetic modifications^24^ and to enforce high levels of discrimination against discrete, single bp changes within a given target^25^. ZFNs would also appear to be less susceptible to pre-existing immunity, as seen with other systems^26^, given their lack of epitopes from human commensal microbes or pathogens (e.g., for the origin of the FokI nuclease^27^, see ref. ^27^). A final attraction is that ZFNs can bind extended targets and are routinely designed for recognition of dimer targets bearing up to 36 bp (Fig. 1a). This facilitates development of highly specific cleavage reagents, as a target of this length will typically exhibit substantial divergence ( > 8 mismatches) from even the most similar non-targeted genomic site.

In developing nucleases for any therapeutic application, a critical requirement is the ability to position the requisite double-strand break event for maximal clinical efficacy. For many applications, this consideration restricts the optimal cleavage target to a narrow sequence window. For example, therapeutic strategies that use homology-directed repair to correct point mutations require close proximity of the cleavage event to the targeted base, with gaps of > 10 bp yielding marked reductions in gene repair efficiencies^28,29^. For other therapeutic approaches, clinical benefit requires disrupting small elements such as transcription factor binding sites^19,30^. Even strategies that target larger regions, such as open reading frames, can be constrained by factors such as the need to cleave a common exon among multiple splice variants, or to avoid cleaving highly similar pseudogenes or homologs. Given these considerations, fully realizing the promise of therapeutic genome engineering will require a high degree of “targeting precision”, i.e., the ability to design a nuclease for efficient cleavage at any chosen base position.

The generation of ZFNs for a chosen target has typically been accomplished via modular assembly^31^ of one- and two-finger units with pre-characterized DNA-binding preferences^32,33^. This approach is simple and has yielded highly active and specific ZFNs for diverse applications^34–36^. However, as practiced, this approach has been implemented in the context of a single, canonical dimer architecture that substantially restricts which flanking sequences can mediate ZFN binding and cleavage of any given base. Each ZFN target must be on opposing DNA strands as shown in Fig. 1a and must also be continuous (i.e., contain no gaps). Developing new dimer architectures that effectively relax these requirements could provide a powerful approach for improving ZFN targeting precision, by enabling any given cleavage event to be mediated by a wider range of alternative ZFN designs.

Here we develop new ZFN architectures for this purpose via two distinct approaches. First, we adapt a cleavage-based bacterial selection system to identify linkers that enable functional reversal of ZFP-FokI cleavage domain order, which allows ZFN recognition of the opposite DNA strand. This yields three alternative dimer configurations to the canonical architecture, increasing design options by a factor of 4 (Fig. 1b, c). Next, we use phage display to identify linkers that allow base-skipping between fingers within a ZFP array. This enables ZFN binding via recognition of alternative triplets and frames (Fig. 1d), and increases design options by a further factor of 16 in the context of our ZFP design platform. Finally, we develop ZFNs for three therapeutic targets and show that our new linkers and the architectures they enable can provide for dense targeting of chosen genomic regions with ZFNs manifesting high levels of activity and specificity.

## Results

### Linker development for improving targeting precision

A key motivation for these studies was the realization that a modest amount of diversification in the linkages between the ZFP and FokI cleavage domain, as well as between adjacent fingers, could yield a large increase in the number of distinct zinc-finger arrays enabling cleavage at a chosen genomic site. This point is illustrated in Fig. 1. The left panels (Figs. 1b, c) show how linkage of the FokI cleavage domain to the amino terminus of a ZFP would enable recognition of either DNA strand flanking the intended cleavage site. The availability of these architectures would increase the number of targeting options for cleaving at a given base by a factor of 4. The right panels (Fig. 1d) show how base-skipping linkers would enable an engineered zinc-finger array to bind alternative, partially frame-shifted flanking sequences with new finger designs, while maintaining the same cleavage site. The option to insert such linkers would allow for exploration of diverse alternative ZFPs, to identify the best-performing ZFN pairs for any given target. Substituting just one or two linkers as shown would increase the number of design options by 4-fold for each ZFN in a dimer and yield an additional 16-fold increase in targeting precision.

### Selection of FokI-ZFP linkers using a bacterial system

We first set out to develop linkers that would enable amino-terminal attachment of the FokI cleavage domain to a ZFP. We pursued this goal via directed evolution, as modeling studies suggested that development of such linkers might constitute a challenging task: the linkers would have to span a large gap (20–25 Å) and also navigate a cleft in the FokI cleavage domain that would impose considerable conformational constraint. To accomplish this, we developed a bacterial selection system that could interrogate very large libraries ( >10^8^ members) to identify those comparatively rare linkers enabling efficient DNA cleavage. In the chosen system, efficient cleavage of a target plasmid is linked to relief from expression of a toxic gene. Similar systems have been used previously for selecting homing endonucleases with altered specificity^37,38^ as well as hyperactive FokI variants^39^.

**Fig. 2:**
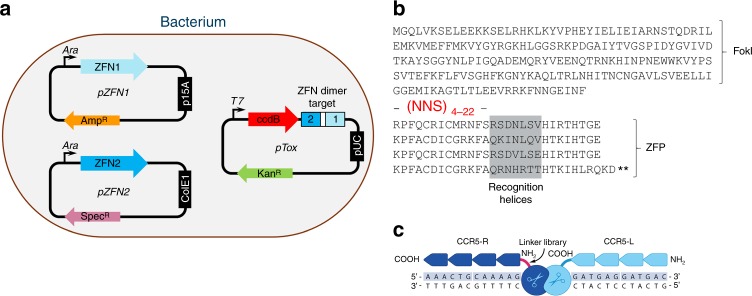
Overview of bacterial selection system and library design. **a** Sketch of a bacterium containing plasmids used for selection. Within the pZFN1 and pZFN2 plasmids (left), each ZFN monomer is placed under control of the inducible arabinose promoter, which allows fine-tuning of the expression level based on the concentration of arabinose in the culture medium. Each expression plasmid contains a different antibiotic resistance marker and also compatible low-copy replication origins. Within the pTox plasmid (at right) the highly lethal topoisomerase inhibitor ccdB is expressed under control of the T7 promoter and a compatible origin of replication. pTox also contains the ZFN dimer target, cleavage of which leads to plasmid clearance. The bacterial cells used in this study express T7 RNA polymerase under control of the *lac* promoter. The cells also express the lac inhibitor and T7 lysozyme. **b** Linker library design. The amino acid sequence of the host ZFN used for selection is shown in single letter code, with the FokI cleavage domain and ZFP regions indicated. The ZFP region is shown as an alignment of the four fingers with recognition helices highlighted in gray. The location and composition of the randomized linker is shown in red (N = mixture of all bases, S = mixture of G and C). The randomized linker library is inserted between the carboxy-terminal residue of the FokI nuclease domain and the first conserved residue in the zinc-finger domain. The library length varies from four to twenty-two residues. **c** Sketch of cleavage target used for selections. The bound ZFN dimer is shown consisting of the fixed right-hand ZFN (CCR5-L) and the left-hand ZFN bearing the linker library (CCR5-R) with randomized linker highlighted in red

An overview of the bacterial selection system is shown in Fig. 2a. The cleavage targets are cloned into a toxin plasmid (pTox) expressing the highly lethal topoisomerase inhibitor ccdB, whereas genes encoding each ZFN are cloned into two additional plasmids for expression (pZFN1 and pZFN2). Expression of the ZFNs leads to cleavage of the target site and clearance of pTox such that cells are able to survive and expand upon subsequent induction of ccdB. A key consideration in the use of this system is the need to completely suppress ccdB when in the uninduced state. This is achieved via the use of an isopropyl β-d-1-thiogalactopyranoside (IPTG)-inducible T7 RNA polymerase system for ccdB expression, with three safeguards against leaky expression as follows: (1) glucose addition to suppress transcription of the polymerase gene in the absence of IPTG; (2) expression of T7 lysozyme to inactivate any low levels of polymerase that are expressed; and (3) use of a GTG start codon in the *ccdB* gene, which reduces translation initiation ~10-fold^40,41^. Selection stringency is enhanced by using low-copy plasmids for ZFN expression and a high-copy replication origin for pTox, thus requiring efficient cleavage for complete clearance. Stringency can be further modulated by adjusting ZFN levels via titration of arabinose in the media^42^ and also adjustment of ZFN induction time.

In spike studies that used ZFN pairs targeting either the gene bearing the AAVS1 safe harbor^43^ or the *CCR5* gene^18^, this system demonstrated an ability to selectively enrich plasmids expressing ZFNs that cleaved pTox from a large excess of plasmid expressing ZFNs that lacked a pTox target. For the AAVS1 ZFNs, the left ZFN was enriched from an excess of left CCR5 ZFN from a frequency of 0.001–95% in six rounds of selection, whereas for the CCR5 ZFNs the left ZFN was enriched from an excess of left AAVS1 ZFN from a frequency of 0.05–99.99% in four rounds of selection (Supplementary Fig. 1).

Having established the system, we proceeded to use it to identify new linkers enabling functional fusion of an amino-terminal FokI cleavage domain. To accomplish this, a DNA segment encoding a randomized linker library was inserted into the gene encoding the CCR5-R ZFN (Fig. 2b) between the carboxy-terminal residue of the cleavage domain and the first conserved residue of the zinc-finger domain^44^. The library featured an NNS randomization scheme (N = mixture of all bases, S = mixture of G and C) with a length range of 4–22 residues and had an estimated size of ~5 × 10^8^. The pTox plasmid was constructed with a cleavage target as in Fig. 2c, in which the binding site for the library-containing ZFN was placed on the same strand as the binding site for the invariant ZFN. Gaps of either 6 or 7 bp were inserted between ZFN target sites, as structural modeling had suggested that 6 bp was likely to be the minimal spacing between ZFN-binding sites that would allow dimerization of the cleavage domains.

Selections were initiated by transforming the linker library into bacterial cells harboring the pTox plasmid containing the targets shown in Supplementary Fig. 2 and the plasmid pZFN2, which expressed the fixed ZFN of the dimer, CCR5-L. Next, ZFNs were induced by the addition of arabinose for 2 h. CcdB toxin was then induced by addition of IPTG followed by incubation overnight and isolation of plasmid DNA. Linker cassettes were PCR-amplified from pZFN1, digested with BsaI-HF, and cloned into freshly prepared pZFN1, to purge any selection events that had occurred outside of the linker sequence. A further eight cycles of selection were performed, with stringency increased at the fifth and seventh rounds via reduction in ZFN induction time to 1 h and then to 30 min. The progress of the selection was monitored by assessing the number of cells containing pTox (Kan^R^) after overnight induction of ccdB. A > 1000-fold reduction in this value during both selections suggested enrichment for ZFNs bearing functional linkers (Supplementary Fig. 2).

Sequencing of selected linkers following rounds four, six, and nine revealed trends suggesting a successful selection (Supplementary Fig. 3). In particular, a strong tendency was observed toward length convergence from the original distribution (4–22 residues), to 10 residues for selections that used the 6 bp gapped target, and to 10–14 residues for selections on the 7 bp gapped target. In addition, we observed small residues at the first two to three positions of most selected linkers, with glycine found at linker position 2 in ~70% of round nine clones. This result is consistent with observations from the crystal structure of FokI, which shows the carboxy terminus of the cleavage domain buried in a cleft^45^, which is expected to impose constraints on the size and flexibility of residues in an adjacent linker sequence.

### Gene-editing activity and portability of selected linkers

Linkers obtained from the selections were then submitted to a four-stage screening process to identify those exhibiting the highest levels of activity and portability. In the first and second stages, linkers were successively screened in the context of their selection host (the CCR5 NC dimer, Fig. 2c) and an alternative NC dimer for the ability to induce indels in K562 cell lines engineered to contain the corresponding cleavage targets (Supplementary Fig. 4). It is noteworthy that although ZFNs with wild-type FokI cleavage domains were used in the selections, these studies and all subsequent cellular studies used ZFNs bearing obligate heterodimer FokI domains^46^. These screens (Supplementary Figs. 4–6 and Supplementary Tables 1–3) identified a subset of linkers that enabled high editing efficiencies by their NC dimer hosts at levels that in many cases matched or exceeded activities manifested by the same ZFNs in the canonical dimer configuration.

In the third screening stage, we assessed how these new linkers functioned in de novo-designed ZFNs generated for endogenous genomic targets. To accomplish this, new ZFNs were designed for cleavage of two sets of ten endogenous sites in the human genome bearing component ZFN targets in an NC or CN configuration, with gaps of 6 or 7 bp. Within each set, ZFNs bearing an amino-terminal FokI cleavage domain were constructed using the top eight candidate linkers identified in stage 2. These ZFNs were then screened for gene modification activity in K562 cells. The results (Supplementary Fig. 7) showed that most linkers yielded average modification activities that matched or exceeded the average activities of a control set of similarly designed canonical ZFNs.

**Fig. 3:**
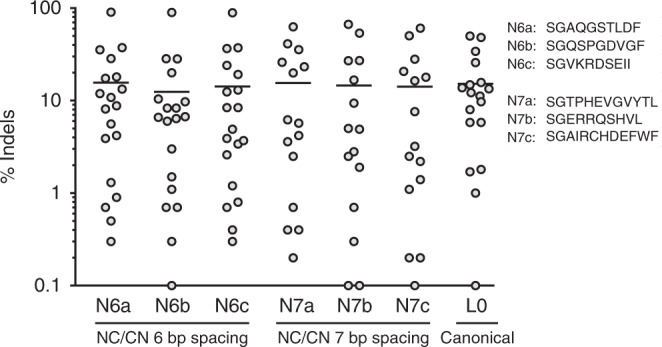
Activity comparison of NC/CN architectures with canonical ZFNs. The NC/CN ZFN architectures were tested in a portability study for their editing efficiency in the context of diverse new ZFN designs. Two sets of 19 NC/CN ZFNs were designed against an endogenous locus (intron 1 of the gene bearing the AAVS1 safe harbor ), one set with a 6 bp spacing, and one with a 7 bp spacing. DNA encoding the top three linkers for their respective spacings (identified in the stage 3 screen, Supplementary Fig. 7) was cloned into an expression vector between the ZFP and FokI domains for each of the designs bearing an amino-terminal FokI cleavage domain. Plasmid DNA for each pair was nucleofected into K562 cells at a dose of 400 ng of DNA per ZFN. Genomic DNA was isolated after three days of incubation and modification was assessed by PCR of the target loci followed by deep sequencing (MiSeq). The results of this study are grouped by architecture type and by linker: NC/CN ZFNs spanning a 6 bp gap with the indicated linker (N6a-N6c), NC/CN ZFNs spanning a 7 bp gap with the indicated linker (N7a-N7c), and canonical ZFNs (L0^62^, included for comparison). Linker sequences are shown at the right. Average values for each group are shown as a solid black bar. Each data point represents a single measurement. For plotted data values see Supplementary Fig. 8. The source data for this figure is available in the Source Data file

In the fourth stage, an additional, larger portability study was performed by designing new ZFNs targeting the first intron of the gene bearing the AAVS1 safe harbor. A total of 19 ZFN pairs were designed to this locus for each of the following configurations: (1) canonical architecture, (2) NC/CN architectures with a 6 bp spacing, and (3) NC/CN architectures with a 7 bp spacing. The ZFN designs with the amino-terminal nuclease domain were constructed with the top three linkers identified in stage three for their respective spacings. These ZFNs were then screened for activity in K562 cells. This study revealed that the NC/CN ZFNs exhibited similar average activity to the canonical ZFNs (Fig. 3, black bars, and Supplementary Fig. 8), with perhaps a wider scatter in the activity levels among the component ZFN pairs. In a final study, we compared our selected linkers with simple glycine-rich sequences for enabling cleavage by the CCR5-targeted NC dimer. As expected, the simple linkers yielded substantially reduced activity, with indel levels reduced by at least fivefold relative to our most active selected linkers (Supplementary Fig. 9).

### Dimers containing two amino-terminal FokI ZFNs

The performance of our linkers in the NC/CN architecture suggested that we could combine two ZFNs containing an amino-terminal FokI cleavage domain to generate an additional new architecture, the NN dimer (Fig. 1c), which is the inverse of a canonical ZFN dimer. To assess this possibility, a new panel of ZFNs was designed targeting sites within intron 1 of the gene bearing the AAVS1 safe harbor. As the optimal gap spacing for this architecture was unknown, ZFNs were designed to recognize dimer sites bearing a range of spacings from 5 to 9 bps. Eleven ZFN pairs were designed for each gap size, with each pair constructed using two to four alternative linkers. These ZFNs were screened for gene modification activity in K562 cells and the results (Supplementary Fig. 10) revealed both a high hit rate and also high average activity for NN pairs spanning 7, 8, and 9 bp gaps, with the highest average indel levels obtained using designs bearing the N6a and N7a linker, and spanning an 8 bp gap.

### Selection of base-skipping linkers via phage display

As our second strategy for increasing ZFN architectural diversity, we sought to develop new linkers that enabled base-skipping between adjacent zinc fingers of an engineered ZFP (Fig. 1d). To accomplish this, linkers were identified via phage display selections in the context of three distinct host ZFPs, each bearing a fully randomized linker library between its second and third finger (Supplementary Fig. 11). Selections were performed for five rounds using a target site containing a degenerate base in the gap between the binding subsites for fingers two and three to bias selection toward linkers that could tolerate any base in the skipped position. A 1000-fold excess of competitor target site bearing either no gap or a 2 bp gap (Supplementary Fig. 12) was added during rounds two through five for increased stringency and to enrich for linkers that enforced skipping of exactly one base. Retention efficiencies rose over the course of the selections (Supplementary Fig. 13) and selected phage pools from the final round showed specific binding to target sites containing a 1 bp gap with a selectivity of up to 26-fold over a non-gapped target (Supplementary Fig. 14). Linkers were sequenced at round five and exhibited strong enrichment for both proline and arginine residues (Supplementary Fig. 15). An enzyme-linked immunosorbent assay (ELISA) study showed ZFPs with the candidate linkers were able to discriminate between a panel of 1 bp gapped targets and a non-gapped target (Supplementary Fig. 16). This study also showed that ZFPs with our selected linkers were more selective for a 1 bp spacing as compared with non-gapped and 2 bp gapped targets, and also had higher ELISA scores than either a flexible linker or other previously described linkers designed to skip 1 bp^47,48^.

Based on these studies we assessed one of the most selective candidate linkers (linker 1c, THPRAPIPKP) for compatibility with ZFN-mediated editing of an endogenous locus (intron one of the gene bearing the AAVS1 safe harbor). ZFNs were designed such that one ZFN in each dimer contained a base-skipping linker. As a comparison set, ten canonical ZFN pairs without skipping linkers were also constructed. Cellular studies in K562 cells revealed comparable average indel levels by both ZFN sets, indicating that our new, skipping linker performed as well as the parental, non-skipping linker (Supplementary Fig. 17). Moreover, these indel levels were substantially higher than those obtained when our selected linker was replaced with a designed 1 bp skipping linker from a prior study (Supplementary Fig. 17a, compare linkers 1c with LRQKDERP).

### Assessing ZFN targeting precision

**Fig. 4:**
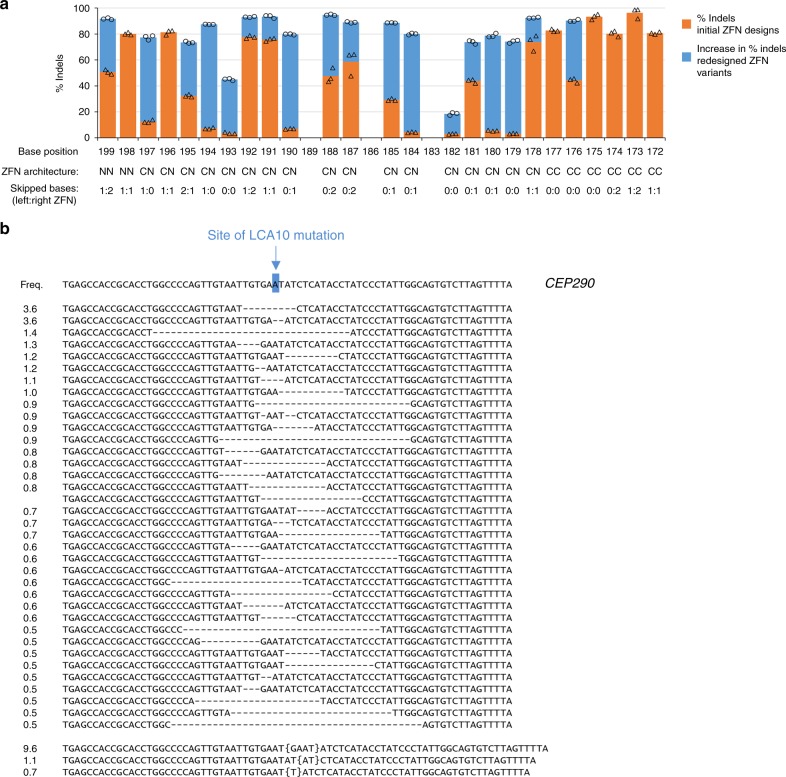
Using new ZFN architectures for high-precision targeting. **a** Indel levels generated in a saturation scanning study of a 28 bp segment of the *HBG1* promoter. ZFNs were designed to center cleavage on as many distinct base steps as possible for positions 172–199 upstream of the transcription start site (corresponding to the sequence 5′-TTCCCCACACTATCTCAATGCAAATATC-3′). ZFN-encoding mRNA was delivered to K562 cells via nucleofection at a dose of 800 ng of mRNA per ZFN, followed by indel quantification via PCR of the target locus and deep sequencing (MiSeq). The *x*-axis indicates base position upstream of the transcription start, defined as the center of cleavage between the two ZFN-binding sites. Details of each dimer are shown below the base position, indicating dimer architecture and the number of skipped bases in each ZFN. Results from the initial screen are shown as orange bars with individual data points provided as triangles, whereas blue extensions indicate the increase in modification observed with ZFNs developed via one additional cycle of design with individual data points provided as circles (e.g., for position 199, the initial designs yielded a dimer inducing 51% indels and the redesigned pair increased the modification by 41% to yield 92% indels). Each transfection was performed in triplicate. **b** Indel types and frequencies induced by a ZFN pair that straddles the site of the LCA10 mutation within the *CEP290* gene. ZFNs were delivered as mRNA to K562 cells via nucleofection of 800 ng of mRNA per ZFN, followed by indel quantification via PCR of the target locus and deep sequencing (MiSeq). At the top of the alignment is the wild-type sequence of the target region in the *CEP290* gene with the location of the LCA10 mutation highlighted in blue and indicated by the arrow. Below this are the deletion and integration events induced by ZFN treatment with frequencies > 0.5% (frequencies noted at the left). The total indel rate induced by this pair was 85% and in the aggregate the indels shown account for 51% of the total indels observed. Target sites for this ZFN are shown in Supplementary Fig. 18. The source data for this figure is available in the Source Data file

As an additional means for evaluating the performance of ZFNs bearing our selected linkers, and to obtain and initial sense of the targeting capabilities afforded by these new design alternatives, we designed ZFNs for a high-density scan of bases 172–199 upstream of the transcription start site in the *HBG1* promoter. This region is of interest to the field of hemoglobinopathies given the association of mutations in this region with hereditary persistence of fetal hemoglobin^49^. To accomplish this, ZFNs using all platform improvements were designed to the region such that the cleavage sites were centered on as many distinct base steps as possible. This yielded ZFN designs for 25 of the possible 28 base steps, which were subsequently screened for activity by delivery of ZFN-encoding messenger RNA in K562 cells. The results of this study are shown in Fig. 4a (orange bars). Active ZFNs were identified for 25 cleavage locations with 16 pairs inducing indels rates above 30% and 10 pairs over 70%. In order to assess the potential to achieve higher cleavage efficiencies at a larger fraction of base steps, ZFNs for a subset of the less-highly modified base steps were submitted to a cycle of redesign that yielded improved variants as shown in Fig. 4a (blue bars). Via this single cycle of redesign, it was possible to identify ZFNs that yielded highly efficient modification ( > 70% indels) at 23 of the 28 base steps. As indicated in Fig. 4a, the resulting ZFN pairs employed all the architectural elements developed in our studies, with all but three pairs using either base-skipping linkers, an amino-terminal FokI linkage, or both. This study showed that our architectural improvements could enable rapid development of ZFNs for dense scanning of a chosen target.

The results of our *HBG1* scan suggested that it should be possible to develop highly active ZFNs for discrete bases of potential therapeutic interest. To test this we targeted cleavage to the site of a point mutation in an intron of the *CEP290* gene that causes a type of Leber congenital amaurosis, LCA10. This mutation disrupts gene function via activation of an otherwise cryptic splice donor element^50^ and it has been proposed that functional elimination of the mutation could be sufficient to disrupt aberrant splicing and restore gene function^51^. ZFNs were designed to cleave at the site of the target mutation and screened via mRNA delivery to K562 cells. An NN ZFN pair (Supplementary Fig. 18 and Supplementary Tables 2 and 3) was identified that was centered on the mutated base and yielded an indel frequency of 85% (Fig. 4b). These data provide further support that ZFNs may be readily generated to cleave at precise locations of interest in the human genome.

### Portability of new linkers to other ZFP design systems

As a number of different platforms have been described for designing ZFPs for novel sequences^52–55^, it was of interest to assess the compatibility of our new linkers and architectures with alternative systems. To accomplish this, we first assessed two well-established alternative finger sets^53,55^ in silico for the ability to target ZFNs to the 2.22 MB human dystrophin gene. This assessment searched for dimers bearing five to six fingers per ZFN, conforming either to the standard canonical configuration (Fig. 1a) or to the expanded set of configurations enabled by our new linkers and amino-terminal FokI (Fig. 1b–d). This analysis–shown in Supplementary Fig. 19–revealed that the addition of the new components developed in this study increased the number of design options by at least 33-fold (from 8 to 267 available designs using the first finger test set^53^ and from 0 to 179 available designs using the second^55^). Next, we tested functionality by assembling 15 pairs from each set (Supplementary Table 4) and screening for activity in K562 cells. For this step, all finger designs were assembled in the context of the framework residues shown in the legend to Supplementary Fig. 20. ZFNs were delivered via mRNA transfection into K562 cells and the results, shown in Supplementary Fig. 20, revealed substantial activity ( > 10% indels) by more than half of tested pairs, with seven pairs exhibiting modification levels above 50%. This study established the compatibility of our linkers and architectures with finger designs developed in other studies.

### Development of highly specific ZFNs for target gene knockout

**Fig. 5:**
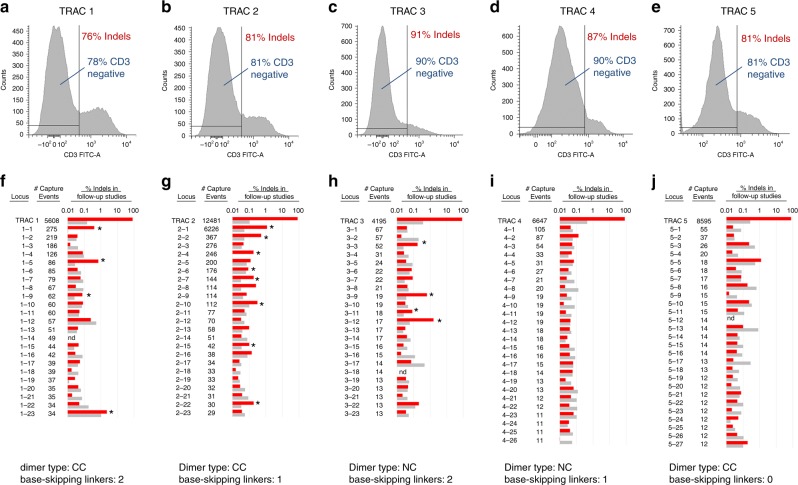
Phenotypic analysis and specificity assessment of ZFNs targeting TRAC. Across the top are shown names of the distinct sites targeted by each pair of ZFNs (Supplementary Fig. 21). Underneath each label are two panels containing the data for the indicated pair (e.g., **a** and **f** for TRAC 1, **b** and **g** for TRAC 2, etc.). Architecture information is indicated at the bottom. **a**–**e** Phenotypic characterization of ZFN-treated T-cells. Histograms for each pair were generated via analysis of 10,000 cells and plots are shown for each of the indicated pairs with the percentage of cells that are negative for CD3 annotated on each graph. CD3 is a component of the T-cell receptor complex queried as a proxy for TRAC surface disruption. Also shown on each plot are the corresponding % indels induced by each ZFN pair in this transfection. Plots for mock transfected T-cells are shown in Supplementary Fig. 22. **f**–**j** Specificity assessment of TRAC ZFNs. Candidate off-target loci were first identified for each pair using an unbiased oligonucleotide duplex-capture assay in K562 cells. In a follow-up study, ZFN-encoding mRNA for each pair was transfected into activated T-cells via BTX transfection. Modification was monitored at each on- and off-target locus by PCR followed by deep sequencing (MiSeq). The off-target characterization for each pair is shown under the corresponding flow cytometry data. On the left of each bottom panel is a table with the locus being monitored (on-target locus at top) and the number of sequences recovered in the capture assay. To the right, log-scale bar graphs are shown that summarize off-target modification in the follow-up indel study. The red bars indicate the % indels for the ZFN-treated T-cells and the gray bars indicate the % indels observed in T-cells treated with mRNA expressing GFP. Candidate loci that were significantly modified in ZFN-treated cells as compared with GFP-treated controls are marked with an asterisk. Statistical significance was determined as previously described^77^ combined with a Bonferroni correction. Each bar represents a single measurement. An expanded dataset for this study is provided in Supplementary Fig. 23. The source data for this figure is available in the Source Data file

Having established that ZFNs could be generated with a high degree of targeting precision, we next sought to deploy the new architectures in an environment where length of the target window was less of a constraint. In such an environment, it was hoped that the availability of a diversity of alternative designs might enable ready identification of highly specific and efficient ZFNs. These studies targeted the T-cell receptor α-constant region (TRAC), as disruption of this gene region is of interest in the field of immuno-oncology as a means of preventing graft-vs.-host response in patients receiving allogeneic CAR T-cells^56^. To accomplish this, ZFNs were designed to the exons of TRAC and were then screened for activity in primary T-cells. An initial screen, followed by two cycles of redesign, yielded five pairs (TRAC 1 through TRAC 5, see Fig. 5), each targeting a distinct cleavage site in TRAC (Supplementary Fig. 21), which exhibited high levels of modification as gauged by deep sequencing as well as flow cytometry of ZFN-treated T-cells (Fig. 5a–e). In addition, four of these ZFN pairs used at least one design component developed in these studies.

These pairs were then assessed for specificity as follows: first, each ZFN pair was submitted to an unbiased oligonucleotide duplex-capture assay to identify a set of candidate off-target loci. For each ZFN pair, this analysis returned the intended cleavage site as the top ranked target (Fig. 5f–j, see columns labeled “Locus” and “# capture events”). Moreover, for three pairs (TRAC 3, TRAC 4, and TRAC 5) the intended target corresponded to the vast majority of recovered sequences ( > 89%), suggesting a high degree of specificity. In the second step of our analysis, ZFNs were delivered to T-cells via electroporation-mediated mRNA delivery, with on-target modification in the range of 79–85%. Genomic DNA was isolated and then candidate off-target loci were queried for indels via PCR amplification followed by deep sequencing. This analysis revealed no statistically significant modification of any candidate off-target locus for two of the pairs (TRAC 4 and TRAC 5; Fig. 5i, j and Supplementary Fig. 23) with low but detectable off-target cleavage seen with the other three pairs (TRAC 1, TRAC 2, and TRAC 3; Fig. 5f–h and Supplementary Fig. 23). Importantly, of the two pairs exhibiting no evidence of off-target cleavage, one was an NC dimer that included a base-skipping linker, demonstrating that the new ZFN architectures and linkers can have comparable activity to canonical ZFNs as well as very high levels of specificity.

## Discussion

Although designed sequence specific nucleases offer considerable promise as therapeutic gene editing agents, ultimate utility for many applications will require an ability to place cleavage events either at a precisely defined base or within a narrow sequence window. As a consequence, assessing and increasing targeting precision has been a longstanding concern in the field^57^, with recent manifestations including efforts to identify or construct new Cas9 variants with broadened or alternative PAM preferences^58–61^, in order to address the targeting limitations inherent to that system. Here we have used architectural diversification as a strategy to substantially increase the targeting precision of ZFNs. Via selection-based methods, we have developed new linker options for spanning finger–finger and finger–FokI cleavage domain junctions that yield a 64-fold aggregate increase in the number of ZFN configurations available for targeting cleavage to any chosen base. In the studies described here, we assessed in aggregate 30 of these new ZFN configurations and identified active nucleases for 97% of those tested. We further show that our new linker components can be combined with available zinc fingers to enable targeting of an arbitrarily chosen genome segment (in *HBG1*) at a density approaching 1.0 (i.e., ZFNs available for targeting almost every base step). To our knowledge, this study provides the first published demonstration of the targeted base-by-base tiling of an endogenous locus with engineered nucleases. Finally, we have shown that these architecture improvements can be used to substantially improve the targeting precision of ZFNs constructed using fingers from other sources.

In this study we have also shown that bacterial selection for relief from ccdB toxicity^37–39^ can provide an effective approach for developing new nuclease architectures. As configured in this study, our ccdB-based system enriched for cleavage-competent species by up to 100-fold per selection cycle (see Supplementary Fig. 1) and, moreover, enabled the isolation of ZFNs with a novel amino-terminal attachment point of the FokI cleavage domain. Given its performance in these studies, we anticipate that this system will find utility in additional nuclease engineering efforts. For example, this system could be applied to selecting improved linkers for the canonical ZFN architecture, as existing linkers were developed via screening of a small panel of discreet linker alternatives^62^. Selections could also be performed to enable a wider range of gap spacings in the context of both canonical and amino-terminal FokI ZFNs such that these longer spacings could be spanned more effectively and provide even more targeting options for a chosen site. Finally, this system could be explored for selecting linkers in the context of other classes of engineered enzymes such as TALENs, FokI-dCas9, and Cas9 base editors, in order to improve the performance of those systems.

These studies have also established that phage systems may be adapted for selection of interfinger linkers that enable selective base-skipping within a ZFN target. A key advantage of using a phage-based system for this purpose is that its in vitro nature allowed the use of competitor (Supplementary Fig. 12) to drive the selection towards more functionally sophisticated linkers that preferred to span gaps bearing exactly one base (i.e., vs. gaps of zero or 2 bp). This yielded linker compositions enriched for both prolines and basic residues (Supplementary Fig. 15), suggesting functional rigidity as well as the potential for bracing of the linker against the DNA via phosphate contacts. Although phage-based systems have been previously applied for selecting new base-contacting interfaces in DNA-binding domains^63,64^ including ZFPs^65,66^, this is, to our knowledge, the first example of their application for identifying structured linkers to enforce relative placement of adjacent domains along the DNA. We anticipate that this approach will find utility for similar applications in the future, e.g., for the development of linkers spanning 2 bp or more between adjacent fingers. Finally, we note that although our base-skipping linkers were developed to increase ZFN targeting capabilities, it is likely to be that they will provide utility outside of this context, given their lack of functional connection to the nuclease domain. We anticipate that they will be useful for diversifying targeting options for other ZFP-fusion proteins, including designed transcription factors^67^ by enabling alternative frame-shifted triplet:finger recognition events for at least a portion of any chosen target (Supplementary Fig. 24).

Genome engineering applications often require the ability to efficiently cleave at a chosen base position for maximum efficacy^19,28–30^. However, even for seemingly permissive applications such as gene knockout, requirements for very high disruption efficiencies may substantially restrict a target landscape to sites that lack occluding histones^68^ or that are unusually prone to misrepair^69^. Such applications can also be constrained by other factors such as the need to cleave a common exon among multiple splice variants, or to avoid cleaving highly similar pseudogenes or homologs. Here we have developed new architectural options for addressing the need for high targeting precision in the context of designed ZFNs and demonstrate their use to achieve both highly dense targeting of a chosen genomic locus, as well has highly active and specific cleavage. The results presented here should broadly facilitate the application of designed ZFNs toward more diverse and challenging applications in research, biotechnology, and therapy.

## Methods

### Components of the bacterial selection system

Three expression vectors were designed to have distinct antibiotic resistance markers and compatible origins of replication. pZFN1 contained the ampicillin resistance gene, the low-copy p15A origin of replication, and ZFN1 under control of the inducible arabinose promoter. Expression from the arabinose promoter is proportional to the level of arabinose in the culture medium allowing the level of ZFN expression to be varied as needed^42^. pZFN2 contained the spectinomycin resistance gene, the low-copy ColE1 origin of replication, and ZFN2 under control of the arabinose promoter. pTox contained the kanamycin resistance gene, the high-copy pUC origin of replication, and the toxic topoisomerase inhibitor ccdB gene under control of the T7 promoter.

The T7 RNA polymerase gene, which is required for expression from the T7 promoter^70^, is inserted into the *lac* operon of the *Escherichia coli* genome. Induction of T7 RNA polymerase is achieved by addition of IPTG^70,71^. The system also used T7 Express lysY/Iq Competent *E. coli* (NEB, Ipswich, MA) for the selection. These cells express the lac inhibitor to suppress expression from the *lac* promoter and also T7 lysozyme, which inactivates any T7 RNA polymerase that may be produced due to leaky expression. These repression mechanisms are then overcome upon the addition of IPTG to the medium.

### Construction of ZFP-FokI linker libraries

Linker libraries were incorporated into the CCR5-R ZFN^18^ (referred to as protein SBS8196z in that study) via PCR with degenerate oligonucleotides. Oligonucleotides were purchased from Integrated DNA Technologies (IDT, Skokie, IL) and the linker length ranged from 4 to 22 residues encoded by an NNS randomization scheme. PCR with these oligonucleotides was performed in combination with a standard primer on the amino-terminal two-finger module for CCR5-R using Accuprime Pfx Polymerase (Thermo Fisher, Waltham, MA) and the carboxy-terminal two-finger module was amplified with standard primers. The reverse primer for the carboxy-terminal module encoded an additional LRQKD(stop) following the terminal histidine residue, which is the natural sequence that terminates the Zif268 protein^44^. All primers also contained BsaI restriction sites to generate complementary overhangs used for ligation of the modules and subsequent ligation into the bacterial expression vector. Sub-libraries for each linker length were amplified separately to avoid length bias during PCR. Following the initial PCR, the reactions were purified using the QIAGEN QIAquick PCR Purification Kit (QIAGEN, Hilden, Germany). The two-finger modules for each library length were then combined with the carboxy-terminal two-finger modules and digested for 2 h with 500 units of BsaI-HF (NEB, Ipswich, MA). Digested amplicons were purified with QIAGEN MinElute PCR Purification Kit and the two fragments were ligated using T4 DNA ligase (Thermo Fisher). Ligated products were run on a 2% agarose gel and extracted using the QIAGEN QIAquick Gel Extraction Kit. Extracted products were then ligated into the bacterial expression vector with T4 DNA Ligase to fuse the assembled ZFP gene containing the linker library to the amino-terminal FokI cleavage domain encoded in the recipient vector (pZFN1). These ligations were then purified by ethanol precipitation. Libraries for each linker length were synthesized individually to avoid length bias during library construction. A library pool was generated by combining 3 µl of each individual library, which was then combined with 300 µl Invitrogen ElectroMAX DH12S cells (Thermo Fisher) and electroporated in one column of 8 using the BTX ECM 630 High Throughput Electroporation System (Harvard Apparatus, Holliston, MA) with a 96-well electroporation plate as per the manufacturer’s protocol. Six of these pools were electroporated in a total of 48 wells, in order to generate the library. All of the electroporations were pooled and recovered for 1 h in 20 ml of 2xYT at 37 °C with shaking at 250 r.p.m. A sample was taken at this point for plating on Luria-Bertani (LB) plates containing ampicillin to determine the number of transformants. The cells were then diluted into 400 ml of 2xYT containing 100 µg/ml ampicillin and 2% glucose, and incubated for 6 h at 37 °C at 250 r.p.m. The cells were then pelleted and resuspended in 2 ml of 2xYT. A volume of 400 µl of resuspended cells was used to extract the DNA library using the QIAGEN HiSpeed Plasmid Maxi Kit.

### Preparation of electrocompetent cells for selections

DNA encoding the CCR5 dimer target site with the NC architecture with either a 6 or 7 bp spacing between ZFN-binding sites was cloned into pTox (Supplementary Fig. 2) via standard cloning techniques. The toxin plasmid was then cotransformed with the CCR5-L expression plasmid (pZFN2) into T7 Express lysY/Iq Competent *E. coli* (NEB) and plated onto LB plates containing 50 µg/ml kanamycin and 25 µg/ml spectinomycin. A single colony was inoculated into 65 ml of 2xYT containing 2% glucose, 50 µg/ml kanamycin, and 25 µg/ml spectinomycin, and shaken overnight at 37 °C and 250 r.p.m. The following morning, 25 ml of the overnight culture was used to inoculate 2 × 500 ml of 2xYT containing 2% glucose, 200 µg/ml kanamycin, and 25 µg/ml spectinomycin in two 2 l flasks. Cells were grown at 37 °C and 250 r.p.m. until an A_600_ of 0.5–0.7. Flasks were chilled on ice for 10 min and then transferred into three 440 ml centrifuge tubes. Cells were then centrifuged at 7860 × *g* for 10 min at 4 °C. The supernatant was removed and the cells were resuspended in 50 ml of chilled 10% glycerol. The resuspended cells were consolidated into one 250 ml centrifuge bottle. Cells were centrifuged at 6375 × *g* for 10 min at 4 °C. The supernatant was removed and the pellet was resuspended in 150 ml of chilled 10% glycerol. This washing procedure was repeated an additional two times. Following the final wash the cells were resuspended in the residual 10% glycerol in the tube. One hundred microliter aliquots were distributed into 1.5 ml microcentrifuge tubes and snap frozen in a dry ice/ethanol bath. Tubes were then transferred to − 80 °C for storage until needed.

### Selection of linkers using the bacterial system

Four micrograms of library maxiprep was mixed with 400 µl electrocompetent cells (6 or 7 bp spacing) and 400 µl of 10% glycerol. A total of 50 µl was distributed into each of 16 wells in a 96-well BTX electroporation plate. Cells were electroporated using a BTX ECM 630 Exponential Decay Wave Electroporation System as per the manufacturer’s protocol. The cells were then recovered in 40 ml of 2xYT with 2% glucose in a 250 ml flask at 37 °C and 250 r.p.m. for 1 h. A sample was taken at this point to determine the number of transformants by plating on LB plates with ampicillin (selectable marker for the library plasmid). Cells were then centrifuged for 10 min at 7860 × *g*. The pellet was resuspended in 80 ml of 2xYT containing 2% glucose, 2.4% arabinose, 150 µg/ml ampicillin, and 25 µg/ml spectinomycin. The cells were transferred to a 250 ml flask and incubated for 2 h at 37 °C and 250 r.p.m. to induce ZFN expression. Cells were then centrifuged for 10 min at 7860 × *g* and resuspended in 500 ml of 2xYT containing 100 mM IPTG, 150 µg/ml ampicillin, and 25 µg/ml spectinomycin, and transferred to a 2 l flask, in order to induce ccdB expression. The flasks were incubated overnight at 37 °C and 250 r.p.m. Following the overnight incubation, 5 ml of culture was taken for miniprep using the QIAGEN QIAprep Spin Miniprep Kit. The linker region between the FokI cleavage domain and the ZFP was then amplified using primers flanking the randomized region and the resulting amplicon was then run on a 2% agarose gel followed by extraction using the QIAGEN QIAquick Gel Extraction Kit. Extracted DNA was then digested with 100 units of BsaI-HF (NEB). Digested linker cassettes were purified with the QIAGEN PCR Purification Kit and ligated into 2 µg of recipient expression vector (pZFN1) with compatible overhangs at 16 °C overnight using T4 DNA Ligase. The following morning, the ligation reactions were ethanol precipitated and used for the transformation to start the next round. Beginning in round 2, a total of eight transformations were done for each selection. Stringency was increased over the course of the selection by reducing the ZFN expression time to 1 h starting in round 5 and then further reducing ZFN expression time to 30 min starting in round 7. Selections were completed after nine rounds.

### Standard indel analysis

PCR primers for the loci of interest were designed using Primer3 with the following optimal conditions: amplicon size 200 nt, Tm 60 °C, primer length 20 nt and G+C content 50%. Adaptors were included in the primers for a second PCR reaction to add the Illumina library sequences (ACACGACGCTCTTCCGATCT forward primer and GACGTGTGCTCTTCCGAT reverse primer). Regions of interest were amplified in 25 µl volume using 5 µl of genomic DNA isolated using QuickExtract (Epicentre) with the QIAGEN HotStarTaq Master Mix Kit. (Note that genomic DNA from T cells was isolated using the MasterPure DNA purification kit (Epicentre).) Primers were used at a final concentration of 0.1 µM with the following cycling conditions: initial melt of 95 °C for 15 min, followed by 30 cycles of 95 °C for 30 s, 55 °C for 30 s and 72 °C for 1 min, followed by a final extension at 72 °C for 10 min. PCR libraries were purified using the QIAquick PCR purification kit (Qiagen). A second PCR reaction was then performed to add sample-specific sequence barcodes. The scheme for the barcode primers is described in ref. ^72^. PCR products were diluted 1:20 in water. Two microlitres of diluted PCR product was used in a 25-µl PCR reaction to add the Illumina library sequences with Phusion High-Fidelity PCR MasterMix with HF Buffer (NEB). Primers were used at a final concentration of 0.5 µM with the following cycling conditions: initial melt of 98 °C for 30 s, followed by 12 cycles of 98 °C for 10 s, 60 °C for 30 s and 72 °C for 40 s, followed by a final extension at 72 °C for 10 min. PCR libraries were purified using the QIAquick PCR purification kit (Qiagen). Samples were quantified with the Qubit dsDNA HS Assay kit (Invitrogen) and diluted to 2 nM. The libraries were then run according to the manufacturer’s instructions on either an Illumina MiSeq with 150 bp paired-end sequencing using a standard 300 cycle kit or an Illumina NextSeq 500 (CEP290 samples only) using a mid-output 300-cycle kit.

### Engineering K562 cell lines with NC target sites

K562 cells (ATCC, Manassas, VA; catalog number CCL-243) were engineered to have an NC CCR5 target site with either a 6 or 7 bp spacing or a canonical CCR5 site inserted into the gene bearing the AAVS1 safe harbor^43^. Analogous cells lines were also generated that inserted an NC AAVS1 target site with either a 6 or 7 bp spacing or a canonical AAVS1 site into the CCR5 target site. Transfections were performed by combining 200 ng of DNA for each expression plasmid with 2E5 K562 cells and 2 µM of a single-stranded oligo donor using the Amaxa 96-well Shuttle System (Lonza, Allendale, NJ) as per the manufacturer’s protocol (oligo sequences in Supplementary Table 1). DNA-PK inhibitor NU7441 (Cayman Chemical, Ann Arbor, MI) was added at a concentration of 2 µM at 4 and 20 h post transfection. Three days post transfection, single-cell clones were generated by pelleting and resuspending cells at a concentration of 0.3 cells/200 µl media and 200 µl was put into each well of two 96-well plates. After 2 weeks of incubation, a sample was taken from each single-cell clone for sequencing the modified loci via PCR followed by deep sequencing using an Illumina MiSeq (Illumina, San Diego, CA). The cell lines with the correct DNA sequence inserted were consolidated, frozen with 5% dimethyl sulfoxide, and stored in liquid nitrogen.

### ZFN design and construction

ZFNs were designed and constructed essentially as described^73^, except that an archive of one- and two-finger modules was used for generating five- and six-finger ZFNs. Nucleases containing six fingers comprised three 2-finger modules, whereas those containing five fingers comprised two 2-finger modules and a single 1-finger module. Sangamo ZFNs and ZFNs from ref. ^57^ were designed from available one- and two-finger module archives. The ZFNs from ref. ^59^ were designed by generating a 2-finger archive of 256 members consisting of all possible combinations of helices from 16 GNN triplets (G = guanine, N = mixture of all bases). The 16 individual fingers were then used for the one-finger archive. For the six-finger ZFNs, base-skipping linkers were deployed as shown in Fig. 1c. For the five-finger ZFNs, base-skipping linkers were inserted between fingers 2 and 3 or fingers 3 and 4. To design ZFNs, target regions were scanned for binding sites of modules in the archive that allow fusion of three modules to form 5- or 6-finger proteins that recognize 15–18 bases, respectively. Nuclease pairs containing gap spacings compatible with the architecture configurations shown in Fig. 1 were then paired resulting in ZFN dimers.

Helices from the alternative systems were first transferred into our one- and two-finger module backbones (Supplementary Fig. 20) before gene assembly. ZFNs were assembled using a PCR-based procedure. In brief, each one- and two-finger module was amplified by PCR in a separate reaction with primers containing BsaI-HF restriction sites. The PCR products that comprise each ZFN are then combined and joined by BsaI-HF (NEB) restriction enzyme digestion followed by ligation into a mammalian expression vector to yield the ZFN expression construct for each nuclease. Design information for the key ZFNs used in these studies is provided in Supplementary Tables 2–4.

### Studies performed in K562 cells

Transfections into K562 cells (either wild-type or engineered cells) were performed by combining 2E5 cells with either plasmid- or mRNA-encoding ZFNs and SF solution (Lonza) using the Amaxa 96-well Shuttle System as per the manufacturer’s protocols. Following nucleofection, cells were placed into RPMI media containing 10% fetal bovine serum. A variation of a transient cold shock protocol^74^ was employed by placing the 96-well plate of cells at 30 °C for 24 h followed by incubation at 37 °C for an additional 48 h. Cells were then pelleted and genomic DNA was isolated using QuickExtract (Lucigen, Middleton, WI) as per the manufacturer’s instructions. Target loci were then analyzed by PCR amplification followed by deep sequencing (MiSeq).

### Studies performed in T-cells

Peripheral blood CD3 + Pan T-cells were purchased from AllCells (Alameda, CA). Transfections into activated T-cells were performed by first pelleting and washing the cells with Dulbecco's phosphate-buffered saline (DPBS) followed by resuspension in BTX electroporation solution (Harvard Apparatus). ZFN-encoding mRNAs were then mixed with 2E5 cells in a volume of 100 µl and transferred to a 96-well electroporation plate. The plate was then placed in a BTX HT-200 Plate Handler (Harvard Apparatus) and transfection was carried out using a BTX ECM 830 Square Wave Electroporation system (Harvard Apparatus) as per the manufacturer’s instructions. T-cells were transferred to a 24-well plate containing 500 µl of pre-warmed media supplemented with 100 units/ml Proleukin (Novartis, Basel, Switzerland) and incubated at 30 °C overnight followed by incubation at 37 °C until 96 h post transfection. Genomic DNA was then isolated using the QIAGEN DNeasy Blood & Tissue Kit (QIAGEN) and target loci were amplified by PCR followed by indel assessment by deep sequencing (MiSeq). All MiSeq samples were processed using background correction by removal of indel-containing sequences that were common to both the ZFN-treated sample and the green fluorescent protein-negative control.

### Flow cytometry of ZFN-treated T-cells

Activated T-cells were transfected as described above with 250 µg/ml of mRNA-encoding 2A-linked^75^ ZFNs. To prepare cells for flow cytometry, approximately 5E5 of ZFN-treated T-cells (or mock transfected T-cells) were pelleted at 300 × *g* for 5 min at room temperature. The supernatant was aspirated and pellets were resuspended in 500 µl of phosphate-buffered saline. Cells were stained with 1 µl of Fixable Viability Dye eFlour 780 (Thermo Fisher) for 10 min on ice. Cells were then pelleted at 300 × *g* for 5 min at room temperature and resuspended in 200 µl staining buffer (1% bovine serum albumin, 0.05% sodium azide in 500 ml DPBS filtered with a 0.22 µm cellulose acetate filter). Cells were then stained by addition of 10 µl of PerCP Mouse Anti-Human CD3 antibody (BD Biosciences, San Jose, CA) for 30 min wrapped in foil at room temperature. Then, 500 µl of lysis buffer (1 × FACS Lysing Solution (BD Biosciences)) was added and cells were incubated for 15 min at room temperature wrapped in foil. Cells were then subjected to flow cytometry on a BD FACSCanto (BD Biosciences) as per the manufacturer’s instructions and the resulting data were gated on CD3-negative cells (Supplementary Fig. 22).

### Identification of candidate off-target loci for TRAC ZFNs

Candidate loci for specificity studies were identified in K562 cells using an oligonucleotide duplex-capture assay^76^. Briefly, K562 cells were transfected with mRNA-encoding TRAC ZFN pairs (TRAC 1–TRAC 5) such that a similar, high level of indels was achieved at each on-target site (~75%). Double-stranded GUIDE-seq oligonucleotides were also included in this transfection at a concentration of 1 µM. Four days post transfection, genomic DNA was extracted using the QIAGEN DNeasy Blood and Tissue kit following the manufacturer’s instructions and 400 ng of genomic DNA was sheared to an average length of 500 bp with a Covaris M220 Focused-ultrasonicator as per the manufacturer’s protocol. Library preparations were performed with the original adapters and primers^76^. Libraries were then quantified with the Qubit dsDNA High Sensitivity Assay kit (Invitrogen) and deep sequencing was performed using an Illumina MiSeq with 150 bp paired-end sequencing using a standard 300 cycle kit. The data processing required clusters of unique oligo integrations within 100 bp of each other. Clusters of integrations also had to have at least fivefold more integrations than the same locus in control cells that lacked a nuclease, individual sequence reads had to map equally well to less than three loci in the hg38 genome, they had to map best to a known region of the hg38 genome, and they had to be at least 10 kb from the intended target.

The protocol used here differed from the published version^76^ in three key aspects as follows: (1) the oligoduplex contained 4 bp degenerate overhangs to facilitate capture at FokI cleavage sites; (2) data processing was fully unbiased (i.e., no target similarity filter was applied); and (3) experiments were run in full quadruplicate (i.e., from transfections through data processing) with a requirement that candidate loci listed in Fig. 5 yield capture events in at least two replicates. Average on-target modification in these studies were as follows: TRAC 1–76.7% ± 1.3%, TRAC 2–74.8% ± 4.6%, TRAC 3–72.9% ± 3.8%, TRAC 4–73.3% ± 2.8%, and TRAC 5–68.8% ± 3.8%. The oligoduplex was constructed from the following oligos:

5′-(Phosphate)-N*N*NNGTTTAATTGAGTTGTCATATGTTAATAACGGT*A*T-3′; 5ʹ-(Phosphate)-N*N*NNATACCGTTATTAACATATGACAACTCAATTAA*A*C-3ʹ

where N represents fully randomized bases, asterisks represent phosphorothioate linkages, and a 5′-phosphate was added to each oligo. Oligos were ordered from IDT and synthesized using bottle mixes for the degenerate bases. Primer sequences for amplifying on-target and candidate off-target loci for the TRAC studies are shown in Supplementary Table 5.

### Code availability

Custom computer scripts used to perform the standard indel analysis and custom computer scripts used to automate more standard portions of the data analysis pipeline are available upon request.

## Supplementary information

Supplementary Information

Source Data

## Data Availability

The datasets generated during and/or analyzed during the current study are available from the corresponding author on reasonable request. Deep sequencing data have been uploaded to the Sequence Read Archive under BioProject accession number PRJNA515925.

## References

[1] Kim YG, Cha J, Chandrasegaran S (1996). Hybrid restriction enzymes: zinc finger fusions to Fok I cleavage domain. Proc. Natl Acad. Sci. USA.

[2] Bibikova M, Golic M, Golic KG, Carroll D (2002). Targeted chromosomal cleavage and mutagenesis in *Drosophila* using zinc-finger nucleases. Genetics.

[3] Bibikova M, Beumer K, Trautman JK, Carroll D (2003). Enhancing gene targeting with designed zinc finger nucleases. Science.

[4] Porteus MH, Baltimore D (2003). Chimeric nucleases stimulate gene targeting in human cells. Science.

[5] Diez B (2017). Therapeutic gene editing in CD34(+) hematopoietic progenitors from Fanconi anemia patients. EMBO Mol. Med..

[6] Chang KH (2017). Long-term engraftment and fetal globin induction upon BCL11A gene editing in bone-marrow-derived CD34(+) hematopoietic stem and progenitor cells. Mol. Ther. Methods Clin. Dev..

[7] DiGiusto DL (2016). Preclinical development and qualification of ZFN-mediated CCR5 disruption in human hematopoietic stem/progenitor cells. Mol. Ther. Methods Clin. Dev..

[8] De Ravin SS (2016). Targeted gene addition in human CD34(+) hematopoietic cells for correction of X-linked chronic granulomatous disease. Nat. Biotechnol..

[9] Torikai H (2016). Genetic editing of HLA expression in hematopoietic stem cells to broaden their human application. Sci. Rep..

[10] Laskowski TJ (2016). Gene correction of iPSCs from a Wiskott-Aldrich syndrome patient normalizes the lymphoid developmental and functional defects. Stem Cell Rep..

[11] Mastaglio S (2017). NY-ESO-1 TCR single edited stem and central memory T cells to treat multiple myeloma without graft-versus-host disease. Blood.

[12] Beane JD (2015). Clinical scale zinc finger nuclease-mediated gene editing of PD-1 in tumor infiltrating lymphocytes for the treatment of metastatic melanoma. Mol. Ther..

[13] Urnov FD (2005). Highly efficient endogenous human gene correction using designed zinc-finger nucleases. Nature.

[14] Li H (2011). In vivo genome editing restores haemostasis in a mouse model of haemophilia. Nature.

[15] Soldner F (2011). Generation of isogenic pluripotent stem cells differing exclusively at two early onset Parkinson point mutations. Cell.

[16] Yusa K (2011). Targeted gene correction of alpha1-antitrypsin deficiency in induced pluripotent stem cells. Nature.

[17] Wood AJ (2011). Targeted genome editing across species using ZFNs and TALENs. Science.

[18] Perez EE (2008). Establishment of HIV-1 resistance in CD4+T cells by genome editing using zinc-finger nucleases. Nat. Biotechnol..

[19] Vierstra J (2015). Functional footprinting of regulatory DNA. Nat. Methods.

[20] Laoharawee K (2018). Dose-dependent prevention of metabolic and neurologic disease in murine MPS II by ZFN-mediated in vivo genome editing. Mol. Ther..

[21] Sharma R (2015). In vivo genome editing of the albumin locus as a platform for protein replacement therapy. Blood.

[22] Dong JY, Fan PD, Frizzell RA (1996). Quantitative analysis of the packaging capacity of recombinant adeno-associated virus. Hum. Gene Ther..

[23] Gammage, P. A. et al. Genome editing in mitochondria corrects a pathogenic mtDNA mutation in vivo. *Nat. Med.* **24**, 1691–1695 (2018). 10.1038/s41591-018-0165-9 PMC6225988 30250142

[24] Choo Y (1998). Recognition of DNA methylation by zinc fingers. Nat. Struct. Biol..

[25] Elrod-Erickson M, Pabo CO (1999). Binding studies with mutants of Zif268. Contribution of individual side chains to binding affinity and specificity in the Zif268 zinc finger-DNA complex. J. Biol. Chem..

[26] Simhadri VL (2018). Prevalence of pre-existing antibodies to CRISPR-associated nuclease Cas9 in the USA population. Mol. Ther. Methods Clin. Dev..

[27] Nakagawa Y, Sakane T, Yokota A (1996). Emendation of the genus *Planococcus* and transfer of *Flavobacterium okeanokoites* Zobell and Upham 1944 to the genus *Planococcus* as *Planococcus okeanokoites* comb. nov. Int. J. Syst. Bacteriol..

[28] Liang X, Potter J, Kumar S, Ravinder N, Chesnut JD (2017). Enhanced CRISPR/Cas9-mediated precise genome editing by improved design and delivery of gRNA, Cas9 nuclease, and donor DNA. J. Biotechnol..

[29] Yang L (2013). Optimization of scarless human stem cell genome editing. Nucleic Acids Res..

[30] Bauer DE (2013). An erythroid enhancer of BCL11A subject to genetic variation determines fetal hemoglobin level. Science.

[31] Segal DJ (2003). Evaluation of a modular strategy for the construction of novel polydactyl zinc finger DNA-binding proteins. Biochemistry.

[32] Beerli RR, Segal DJ, Dreier B, Barbas CF (1998). Toward controlling gene expression at will: specific regulation of the erbB-2/HER-2 promoter by using polydactyl zinc finger proteins constructed from modular building blocks. Proc. Natl Acad. Sci. USA.

[33] Kim HJ, Lee HJ, Kim H, Cho SW, Kim JS (2009). Targeted genome editing in human cells with zinc finger nucleases constructed via modular assembly. Genome Res..

[34] Perez-Pinera P, Ousterout DG, Brown MT, Gersbach CA (2012). Gene targeting to the ROSA26 locus directed by engineered zinc finger nucleases. Nucleic Acids Res..

[35] Sander JD (2011). Selection-free zinc-finger-nuclease engineering by context-dependent assembly (CoDA). Nat. Methods.

[36] Shukla VK (2009). Precise genome modification in the crop species Zea mays using zinc-finger nucleases. Nature.

[37] Chen Z, Zhao H (2005). A highly sensitive selection method for directed evolution of homing endonucleases. Nucleic Acids Res..

[38] Doyon JB, Pattanayak V, Meyer CB, Liu DR (2006). Directed evolution and substrate specificity profile of homing endonuclease I-SceI. J. Am. Chem. Soc..

[39] Guo J, Gaj T, Barbas CF (2010). Directed evolution of an enhanced and highly efficient FokI cleavage domain for zinc finger nucleases. J. Mol. Biol..

[40] O’Donnell SM, Janssen GR (2001). The initiation codon affects ribosome binding and translational efficiency in *Escherichia coli* of cI mRNA with or without the 5’ untranslated leader. J. Bacteriol..

[41] Van Etten WJ, Janssen GR (1998). An AUG initiation codon, not codon-anticodon complementarity, is required for the translation of unleadered mRNA in *Escherichia coli*. Mol. Microbiol..

[42] Johnson CM, Schleif RF (1995). In vivo induction kinetics of the arabinose promoters in *Escherichia coli*. J. Bacteriol..

[43] Hockemeyer D (2009). Efficient targeting of expressed and silent genes in human ESCs and iPSCs using zinc-finger nucleases. Nat. Biotechnol..

[44] Pavletich NP, Pabo CO (1991). Zinc finger-DNA recognition: crystal structure of a Zif268-DNA complex at 2.1 A. Science.

[45] Wah DA, Hirsch JA, Dorner LF, Schildkraut I, Aggarwal AK (1997). Structure of the multimodular endonuclease FokI bound to DNA. Nature.

[46] Doyon Y (2011). Enhancing zinc-finger-nuclease activity with improved obligate heterodimeric architectures. Nat. Methods.

[47] Kim JS, Pabo CO (1998). Getting a handhold on DNA: design of poly-zinc finger proteins with femtomolar dissociation constants. Proc. Natl Acad. Sci. USA.

[48] Nomura W, Sugiura Y (2003). Effects of length and position of an extended linker on sequence-selective DNA recognition of zinc finger peptides. Biochemistry.

[49] Gilman JG, Huisman TH (1985). DNA sequence variation associated with elevated fetal G gamma globin production. Blood.

[50] den Hollander AI (2006). Mutations in the CEP290 (NPHP6) gene are a frequent cause of leber congenital amaurosis. Am. J. Human. Genet..

[51] Ruan GX (2017). CRISPR/Cas9-mediated genome editing as a therapeutic approach for leber congenital amaurosis 10. Mol. Ther..

[52] Choo Y, Klug A (1994). Selection of DNA binding sites for zinc fingers using rationally randomized DNA reveals coded interactions. Proc. Natl Acad. Sci. USA.

[53] Gupta A (2012). An optimized two-finger archive for ZFN-mediated gene targeting. Nat. Methods.

[54] Sander JD (2011). Selection-free zinc-finger-nuclease engineering by context-dependent assembly (CoDA). Nat. Methods.

[55] Segal DJ, Dreier B, Beerli RR, Barbas CF (1999). Toward controlling gene expression at will: selection and design of zinc finger domains recognizing each of the 5’-GNN-3’ DNA target sequences. Proc. Natl Acad. Sci. USA.

[56] Torikai H (2012). A foundation for universal T-cell based immunotherapy: T cells engineered to express a CD19-specific chimeric-antigen-receptor and eliminate expression of endogenous TCR. Blood.

[57] Maeder ML (2008). Rapid “open-source” engineering of customized zinc-finger nucleases for highly efficient gene modification. Mol. Cell.

[58] Hirano H (2016). Structure and engineering of *Francisella novicida* Cas9. Cell.

[59] Kim E (2017). In vivo genome editing with a small Cas9 orthologue derived from Campylobacter jejuni. Nat. Commun..

[60] Kleinstiver BP (2015). Broadening the targeting range of Staphylococcus aureus CRISPR-Cas9 by modifying PAM recognition. Nat. Biotechnol..

[61] Zetsche B (2015). Cpf1 is a single RNA-guided endonuclease of a class 2 CRISPR-Cas system. Cell.

[62] Bibikova M (2001). Stimulation of homologous recombination through targeted cleavage by chimeric nucleases. Mol. Cell. Biol..

[63] Nilsson MT, Widersten M (2004). Repertoire selection of variant single-chain Cro: toward directed DNA-binding specificity of helix-turn-helix proteins. Biochemistry.

[64] Simon MD, Sato K, Weiss GA, Shokat KM (2004). A phage display selection of engrailed homeodomain mutants and the importance of residue Q50. Nucleic Acids Res..

[65] Jamieson AC, Kim SH, Wells JA (1994). In vitro selection of zinc fingers with altered DNA-binding specificity. Biochemistry.

[66] Rebar EJ, Pabo CO (1994). Zinc finger phage: affinity selection of fingers with new DNA-binding specificities. Science.

[67] Tan S (2003). Zinc-finger protein-targeted gene regulation: genomewide single-gene specificity. Proc. Natl Acad. Sci. USA.

[68] Yarrington RM, Verma S, Schwartz S, Trautman JK, Carroll D (2018). Nucleosomes inhibit target cleavage by CRISPR-Cas9 in vivo. Proc. Natl Acad. Sci. USA.

[69] van Overbeek M (2016). DNA repair profiling reveals nonrandom outcomes at Cas9-mediated breaks. Mol. Cell.

[70] Tabor S, Richardson CC (1985). A bacteriophage T7 RNA polymerase/promoter system for controlled exclusive expression of specific genes. Proc. Natl Acad. Sci. USA.

[71] Jacob F, Monod J (1961). Genetic regulatory mechanisms in the synthesis of proteins. J. Mol. Biol..

[72] Miller JC (2015). Improved specificity of TALE-based genome editing using an expanded RVD repertoire. Nat. Methods.

[73] Doyon Y (2008). Heritable targeted gene disruption in zebrafish using designed zinc-finger nucleases. Nat. Biotechnol..

[74] Doyon Y (2010). Transient cold shock enhances zinc-finger nuclease-mediated gene disruption. Nat. Methods.

[75] Ryan MD, Drew J (1994). Foot-and-mouth disease virus 2A oligopeptide mediated cleavage of an artificial polyprotein. EMBO J..

[76] Tsai SQ (2015). GUIDE-seq enables genome-wide profiling of off-target cleavage by CRISPR-Cas nucleases. Nat. Biotechnol..

[77] Pattanayak V, Ramirez CL, Joung JK, Liu DR (2011). Revealing off-target cleavage specificities of zinc-finger nucleases by in vitro selection. Nat. Methods.

