# Supplementary material for: Diversifying the structure of zinc finger nucleases for high-precision genome editing
Source: Nat Commun. 2019 Mar 8;10:1133. doi: 10.1038/s41467-019-08867-x (PMC6408524; doi:10.1038/s41467-019-08867-x)
Supplement: Supplementary file 1 — Supplementary Information [file 41467_2019_8867_MOESM1_ESM.pdf]

Supplementary Materials for

Diversifying the Structure of Zinc Finger Nucleases for High-Precision Genome Editing

Paschon et al.

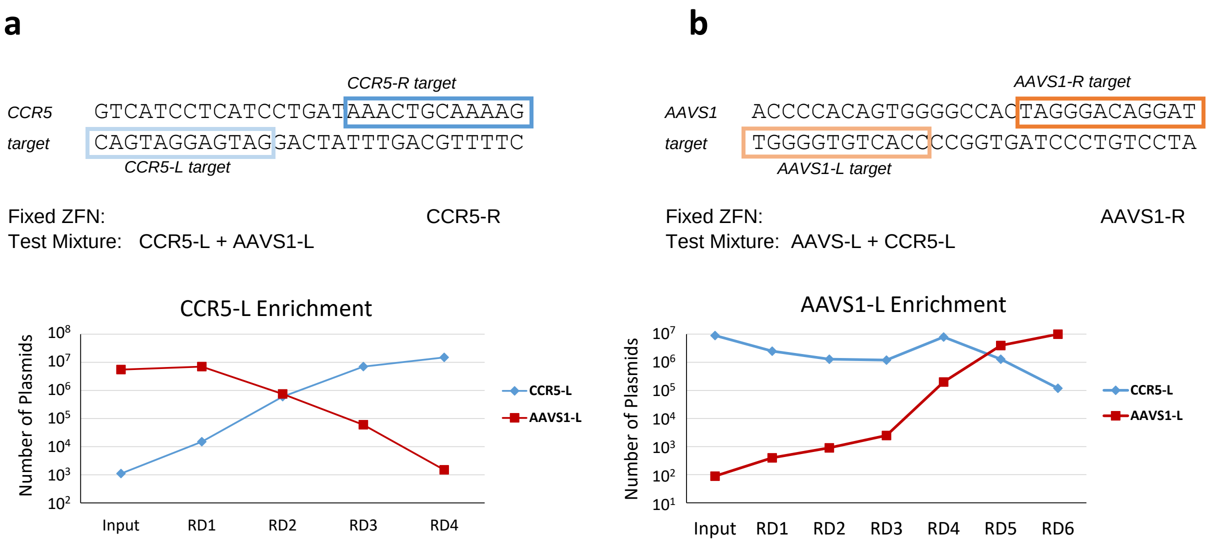

Supplementary Figure 1

Spike studies used to characterize the bacterial selection system.

(**a**) Enrichment of a plasmid encoding the CCR5-L ZFN[1] (referred to as SBS8266 in that study) from a vast excess of plasmid encoding the AAVS1-L ZFN[2]. At the top is the dimer cleavage target site used for selection with individual ZFN binding sites boxed in blue (CCR5-L on the left and CCR5-R on the right). Beneath the target site is noted that for this spike study CCR5-R was fixed while the test mixture contains both CCR5-L and an unrelated ZFN, AAVS1-L.

The CCR5 dimer site was cloned into the pTox plasmid whereas genes encoding the ZFNs were cloned into pZFN1 and pZFN2 (**Fig. 2a**). The spike study was performed as follows: 1) a mixture of CCR5-L and AAVS1-L plasmids was made containing an approximately 2000-fold excess of AAVS1-L, 2) the plasmid mixture was transformed into cells bearing the pTox plasmid and a plasmid expressing the fixed ZFN in the study (CCR5-R), 3) the ZFNs were induced for two hours by addition of arabinose into the culture media, 4) the ccdB protein was induced by addition of IPTG to the culture media followed by overnight incubation, 5) plasmids were then prepped and ZFN genes were subcloned into freshly-prepared pZFN plasmid in order to minimize accumulation of any potential background events, and 6) steps two to five were repeated for up to five more cycles. The level of each test plasmid was quantified in the initial mixture and following each round of selection via qPCR with primers designed to specifically detect each test plasmid. Resulting data are shown in the graph at the bottom of the panel. Each data point represents the average of duplicate qPCR reactions.

(**b**) Inverse study in which plasmid expressing the AAVS1-L ZFN was enriched from a large excess of CCR5-L expressing plasmid.

a

CCR5-R target site: AAACTGCAAAAG
CCR5-L target site: GATGAGGATGAC

6 bp spacing: AAACTGCAAAAGctgcatGATGAGGATGAC
7 bp spacing: AAACTGCAAAAGctgacatGATGAGGATGAC

b

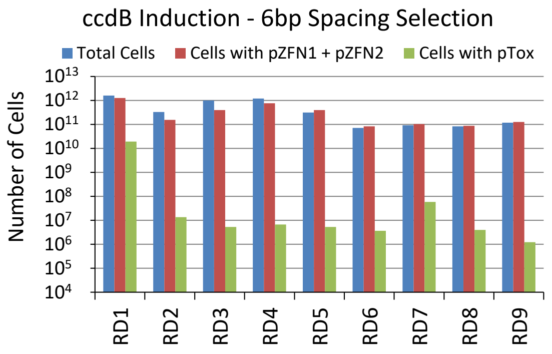

c

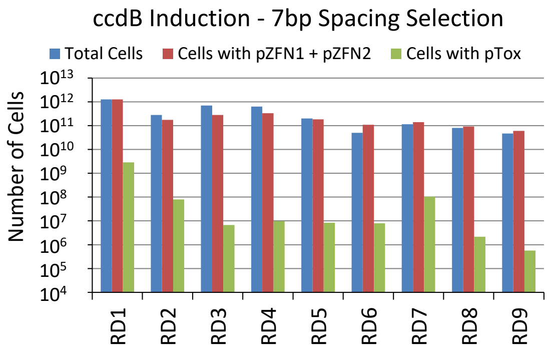

Supplementary Figure 2

Selection target sites and monitoring the progress of the selections.

(**a**) Target sites used for the bacterial selections. At the top are the target sites for the CCR5 ZFNs. At the bottom are the full dimer sites cloned into pTox used for selections with the indicated spacing (in bp) between targets. (**b**) Progress of the selection for linkers enabling the NC architecture with a 6 bp spacing as gauged by depletion of the Kan resistance marker (pTox plasmid) after each selection cycle. Following ccdB induction and overnight growth, a sample of bacterial cells was plated onto LB plates containing no antibiotic (blue bars, total cells), ampicillin/streptomycin (red bars, pZFN1 and pZFN2), or kanamycin (green bars, pTox). A drop in the fraction of cells containing pTox is indicative of the selection enriching for linkers enabling functional nucleases leading to cleavage and clearance of the pTox plasmid. (**c**) Same as in (**b**) but for the selection with a 7 bp spacing.

a

Round 4			Round 6			Round 9		
L6-1	SGVFSTMTHD		L6-29	SGISSVWS		L6-54	SGMPAMPYGL	
L6-2	STVPNAQRELIY		L6-30	FKPWYDEHVPLGAP		L6-55	SGVEHSLYGA	
L6-3	SGLTDGCVHHYLDC	x2	L6-31	TGGTMWRPEY		L6-56	SGNECSRFRP	
L6-4	SGIVQTWIPY		L6-32	SPFPASVLDY		L6-57	SGRSPEMDWC	x2
L6-5	AGAQSSQWDP		L6-33	SGFDLTRGMLAQ		L6-58	PNSYGLNPQLKT	
L6-6	SGTSSTRDLC		L6-34a*	SGLHYDDLDL		L6-59	SGVATRHVMG	x2
L6-7	AGRTGVTIDL		L6-34b*	SGAIYARPIE		L6-60a*	SGAQGSTLDF	
L6-8	GPRGFKEYYD		L6-35	DPTFSKPYSP		L6-60b*	SGLPMGSYGS	
L6-9	SGSTARMVGS		L6-36	SGCVSTIKAD		L6-61	SGVPSRDF	
L6-10	SGQPCALQWV		L6-37	SGKVRVSMFG		L6-62	TGRPSPNYGV	
L6-11	SGTYPDPLSD		L6-38	CEAGERLPVWSE		L6-63	SGTKSSSDIC	
L6-12	SGLSSLDY		L6-39	TGNFNQGIVD		L6-64	SGSRFQHDFL	
L6-13	VADGTTTRNKSYETWC		L6-40	SGTPVNVGLY		L6-65	SGNIRVHPSY	
L6-14	SATPSSRIEL		L6-41	SGELVQFIGH		L6-66	VNNWGLSSLCPP	
L6-15	SGCKTAKPLL		L6-42	DSGIPRLSPP		L6-67	SGQSPGDVGF	
L6-16	GGVTKGTWSD		L6-43	SGDIPRYYVS		L6-68	GEAPNTPYAP	
L6-17	SGSRTEIDVL		L6-44	SGVIKRVDTP		L6-69a*	SGVKRDSEII	
L6-18	SGTRGMLHFP		L6-45	SGTYPLTFLH		L6-69b*	GASLGPPWCP	
L6-19	TGGELSDTSYAL		L6-46	SGNTVVYSVV		L6-70	SGEVANAGLW	
L6-20	SGAPSCSRSWLF		L6-47	SGRVGWRP		L6-71	SGTRSSDLSC	
L6-21	GAYSTTAFRP		L6-48	GSRMSKQPCWYP		L6-72	DNPHFSYQRLRS	
L6-22	SGNILREVGYWS		L6-49	SGIYTSESLI	x2	L6-73	GYGPWSLTLPRFHG	
L6-23	SGTIKVFAQD		L6-50	SFSANFVVSKPYMG		L6-74	TGLPSKVYGA	
L6-24	SERVMVRYEP		L6-51	GDVASTFGENFY		L6-75	SGSLRGVDPMWH	x3
L6-25	EETPVRQYRP		L6-52	EEKGMLTSARSELV		L6-76	SGTEPWRP	
L6-26	GQDMGLNRSSEWCA		L6-53	SGTSTICEYH				
L6-27	GPPGDRRWAISS							
L6-28	SGVMPLKLLD							

b

Round 4		Round 6		Round 9	
L7-1	LACRPAQPPP	L7-27	TGCKSVPRVGCI	L7-51	SGVFSNPRCA
L7-2	ARSDCPVYVQLADNID	L7-28	WDDPTDPIARSPAECL	L7-52a*	SGTFAVSGVS
L7-3	TGTTVLVSAL	L7-29	EGAKRVEQLR	L7-52b*	GGRALSCMSRDKIV
L7-4	SQATPTLYYTPL	L7-30	SGERRQSHVL	L7-53	SPGIRSSDPYIM
L7-5	RTSGPPNTHRESIESN	L7-31	LGAQPSKLVRSA	L7-54	SGTKWIRSMA
L7-6	RAGCAKSRVD	L7-32	GVETAWVGSLVN	L7-55	SGFNHSSCDVVY
L7-7	SGALQEPWSI	L7-33	TQFRSPEVII	L7-56	SGLVARTSDGFE
L7-8	SERVVMNSIG	L7-34	RLLTHGASPPLL	L7-57	GISQGSYPIS
L7-9	GMLRREVRQAELWE	L7-35	SGRSVAAIGN	L7-58	GGVKSVYPFI
L7-10	IQPSQSQMGKRQMMVV	L7-36	WLSGRTSAHAPDLY	L7-59	SGHPLIVPSM
L7-11	GGWKTLAKDWAW	L7-37	YELSGNGTRSHEWPCS	L7-60	GPFRKPRNAAIH
L7-12	ASGENLGPVRIPEKLA	L7-38	GVVASEFGIDGPWS	L7-61	SGMSSDLLHVTL
L7-13	SAPLMWQAYRRCPDVL	L7-39	VTPARIDHMPIL	L7-62	QEQVSERDYHRMEY
L7-14	RMRPRMTKDSVD	L7-40	LNMPSIQPEV	L7-63	SGSRIALTTN
L7-15	SMTVRKHLNAQKLC	L7-41	GLHPVTSSVL	L7-64	QSGKIASPHVVI
L7-16	SGAIRCHDEFWF	L7-42	GFWSTAHKINFEES	L7-65	GSKRTASTWTVV
L7-17	TTEIDGALTQVPLH	L7-43	TGSMVKCSVV	L7-66	GGSQTNQVIR
L7-18	QTMANPGFCSWVND	L7-44	WGKPYSMGDY	L7-67	TGRIVPKESV
L7-19	ELADDNFARRQVIN	L7-45	SGTLPFHYTC	L7-68	GPKNFDNEEFLH
L7-20	TGARKTLLPEMF	L7-46	SVRRMKQESREC	L7-69	SGAARTEDSY
L7-21	EYLSRSRDYKDAFS	L7-47	GGSRKRVGPFAAYE	L7-70	TGLPHVRECV
L7-22	LGHAAGSAGRGTSV	L7-48	SCRRLASDVAIS	L7-71	SGTPHEVGVYTL
L7-23	LGKTECTLYRTN	L7-49	SGIVCSHSSA	L7-72	NTNRSRVNLVIE
L7-24	QTGSMRQGTSLGHI	L7-50	EQGDPRQGGHWSTSMH	L7-73	SGLFSMPIAT
L7-25	SGQPMFSWSD			L7-74	FGESIFRPAP
L7-26	RLPALGSLSKYEPGVP			L7-75	GNTVSTSGIV
				L7-76	TGTQSRSYAY

Supplementary Figure 3

Linker sequences obtained from bacterial selections.

Linker cassettes were sequenced following rounds four, six, and nine, the final round of selection. The amino acid sequence for the linkers obtained for selection with a 6 bp spacing are shown in (**a**) whereas the amino acid sequence for the linkers obtained for selection with a 7 bp spacing are shown in (**b**). If multiple clones were obtained with the same sequence, the number of instances is shown to the right of the linker sequence. Those linkers marked with an asterisk (*) denote clones that contained a mixture of linkers in the initial sequencing run but were subsequently separated after confirmation of activity in the initial screen in mammalian cells.

a

Cell lines for stage 1 screens

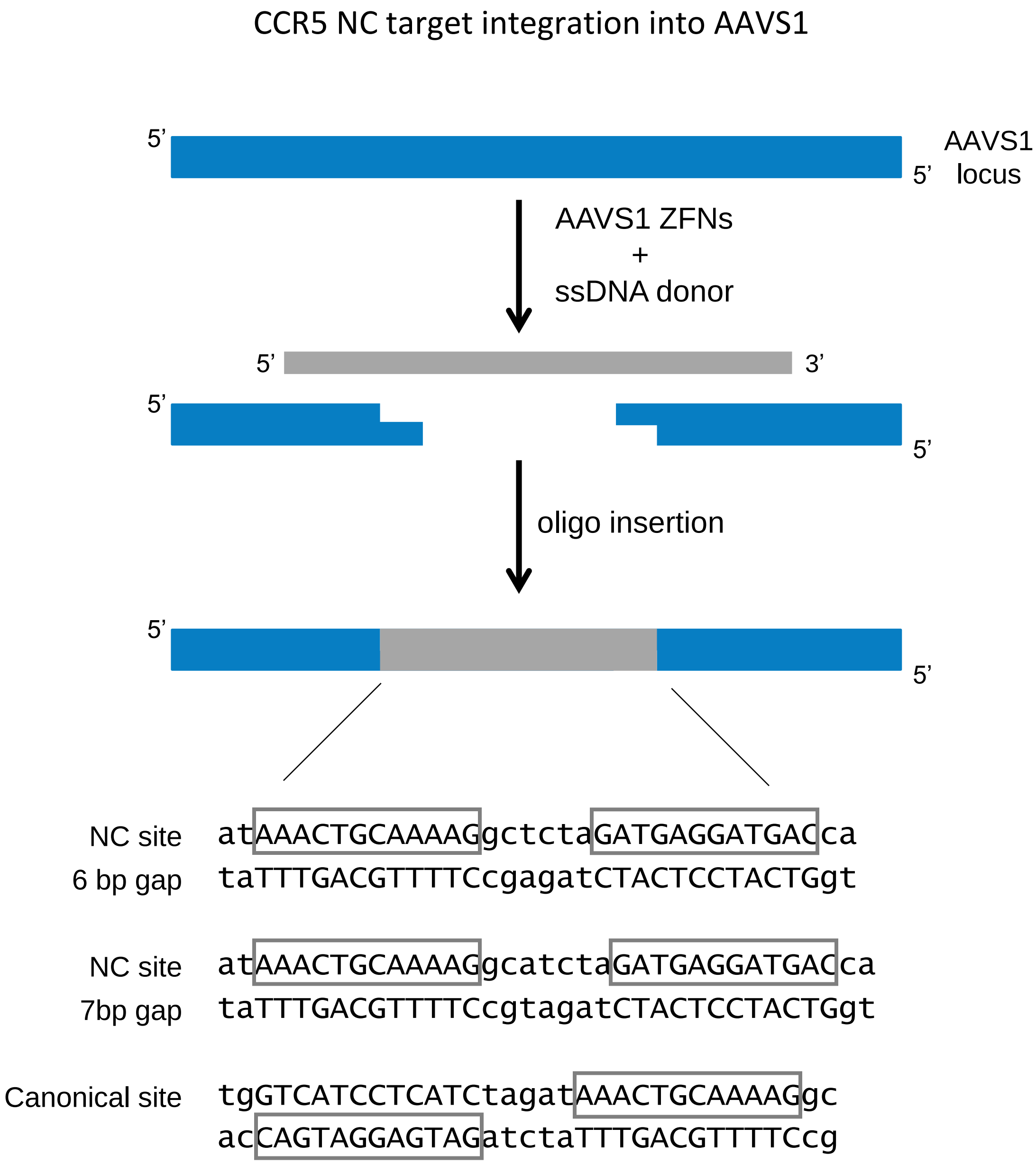

b

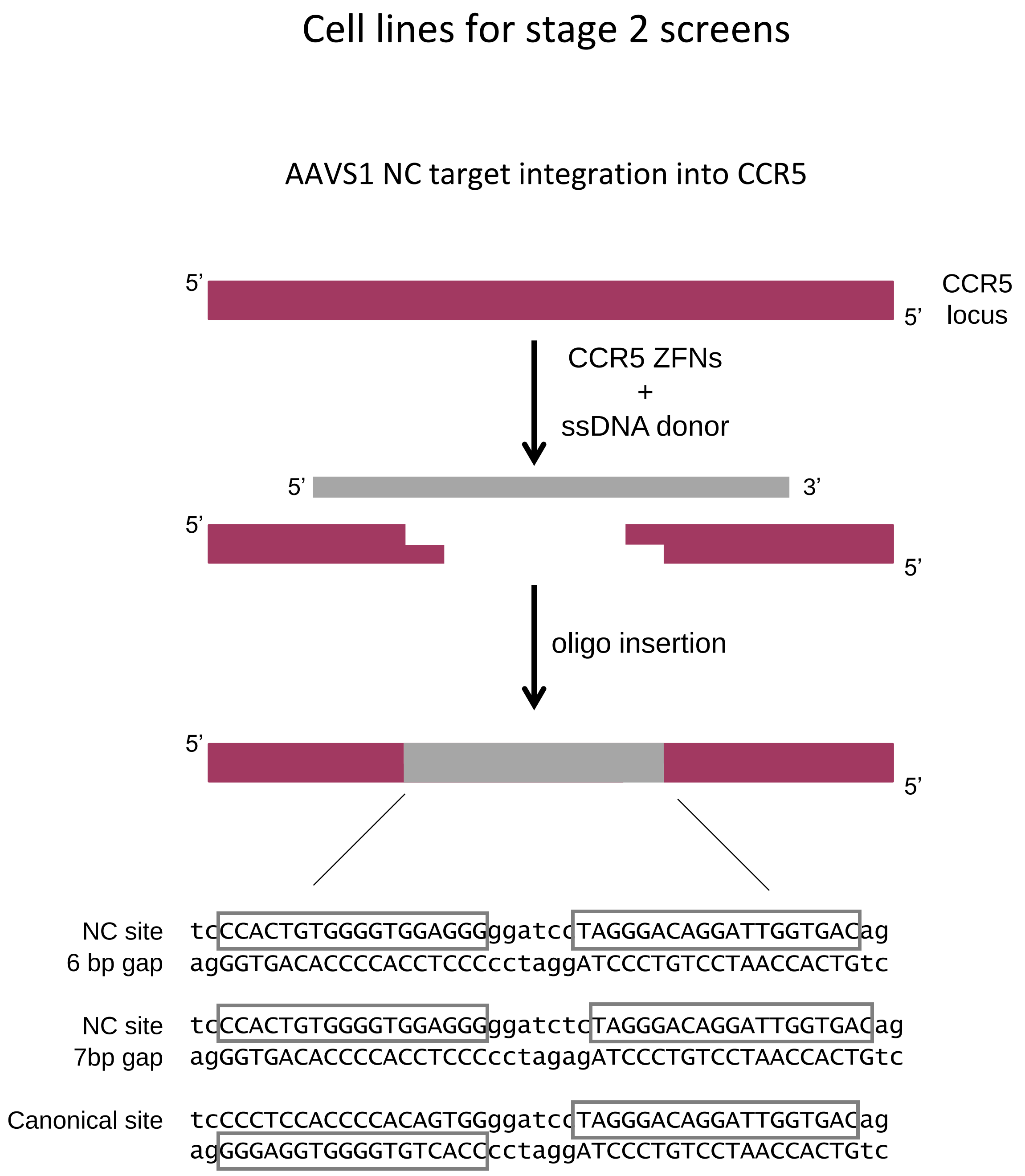

Supplementary Figure 4

Generation of K562 cell lines bearing NC targets for screening selected linkers.

(**a**) Generation of screening lines used for stage 1 screens. Three K562 cell lines were generated in order to perform the first stage of screening: two bearing NC versions of the CCR5 target with a gap of 6 or 7 bp, integrated into the AAVS1 locus as shown, and a third bearing a similarly

integrated canonical CCR5 target, generated for control studies of the canonical CCR5 ZFN dimer. To accomplish this, cells were nucleofected with 200ng of DNA encoding each ZFN and 2 μM single-stranded oligo donor bearing the ZFN target site to be integrated. The DNA-PK inhibitor NU7441 was added to the cells at a concentration of 1 μM after four and 20 hours in order to bias repair towards homology-directed repair. After three days of recovery, single-cell clones were isolated by diluting cell pools into 96-well plates at a density of 0.3 cells/well. Cells were expanded for two weeks, genomic DNA was isolated, and the modified locus was sequenced by MiSeq. At the bottom are the DNA sequences of the three integrated target sites. ZFN target sites are boxed in gray. Note that the cell line bearing the canonical dimer target was generated so that the activity of the canonical ZFN and NC ZFNs could be compared at the same genomic locus. (**b**) As in (**a**) except that targets were for the stage 2 screens and were generated by integrating NC versions of the AAVS1 target into the CCR5 locus as shown, with a canonical AAVS1 target also integrated as a control. Single-stranded oligo sequences are shown in **Supplementary Table 1**.

a

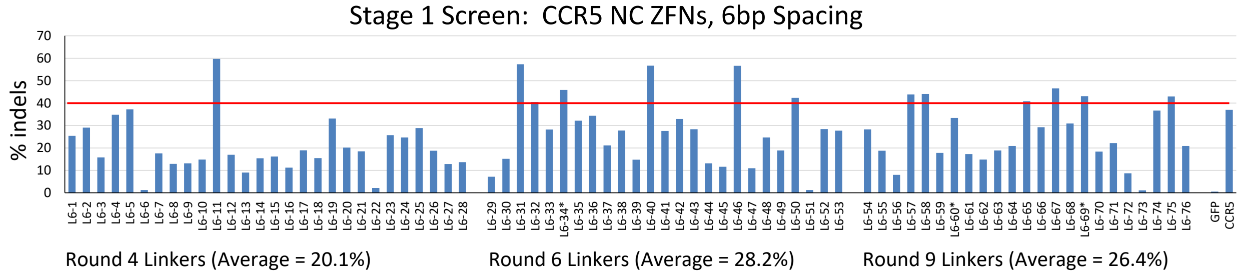

b

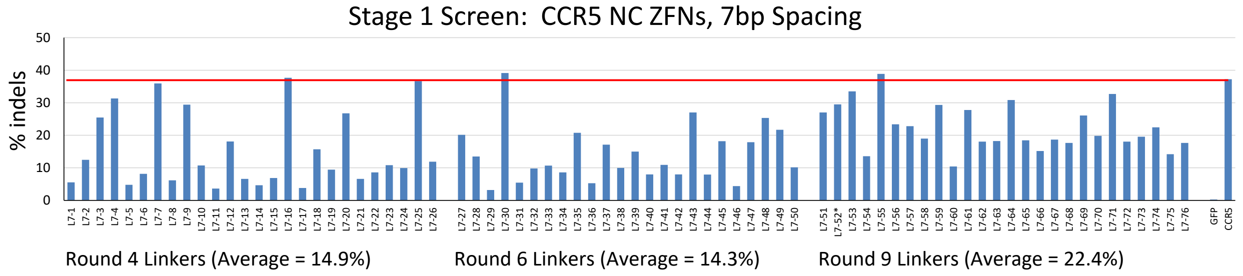

Supplementary Figure 5

Stage 1 screen of CCR5 NC ZFNs with selected linkers in K562 cells.

(**a**) Activity screen of linkers selected using the NC site with a 6 bp gap. Linkers were screened in their selection context (i.e. the NC CCR5 ZFN dimer) for modification activity in a cell line bearing an integrated NC CCR5 target with a 6 bp gap. Linkers are grouped by selection cycle from which they were isolated with the average activity observed within each group indicated. As a control, the parent canonical CCR5 ZFN dimer was also screened for activity in a cell line constructed to bear an integrated copy of its target at the same locus. The percent modification induced by this control ZFN is indicated by the bar at right and by the red line across the chart. Cells were transfected with 200ng of plasmid DNA for each ZFN and genomic DNA was isolated following three days of incubation. Target loci were then assessed for modification via deep sequencing of PCR amplicons. For details on cell line construction see **Supplementary Figure 4**. (**b**) As in (**a**), except that the linkers were selected using a target sequence with a 7bp gap and screened in cells bearing an integrated copy of the 7-bp gapped target. Each data point represents a single measurement.

a

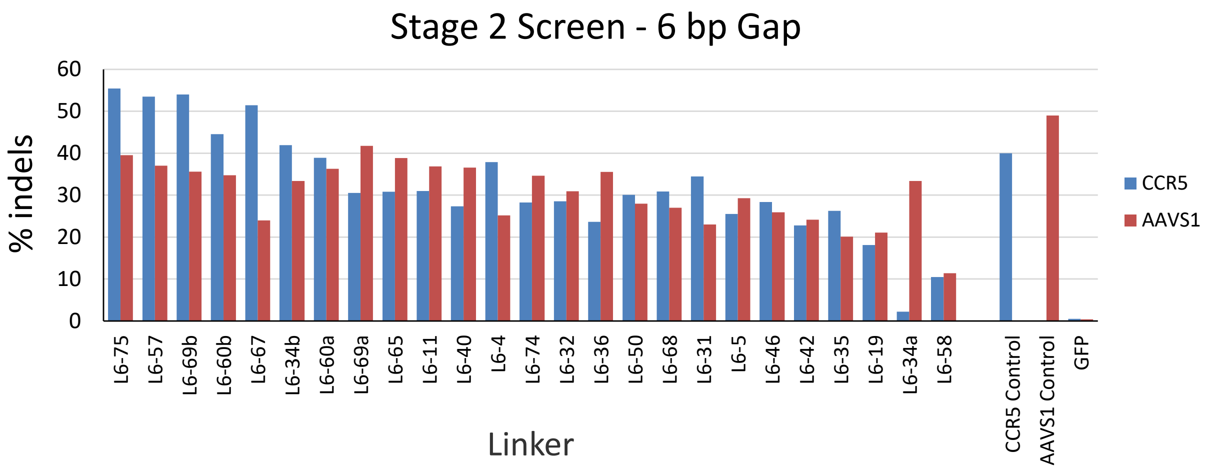

b

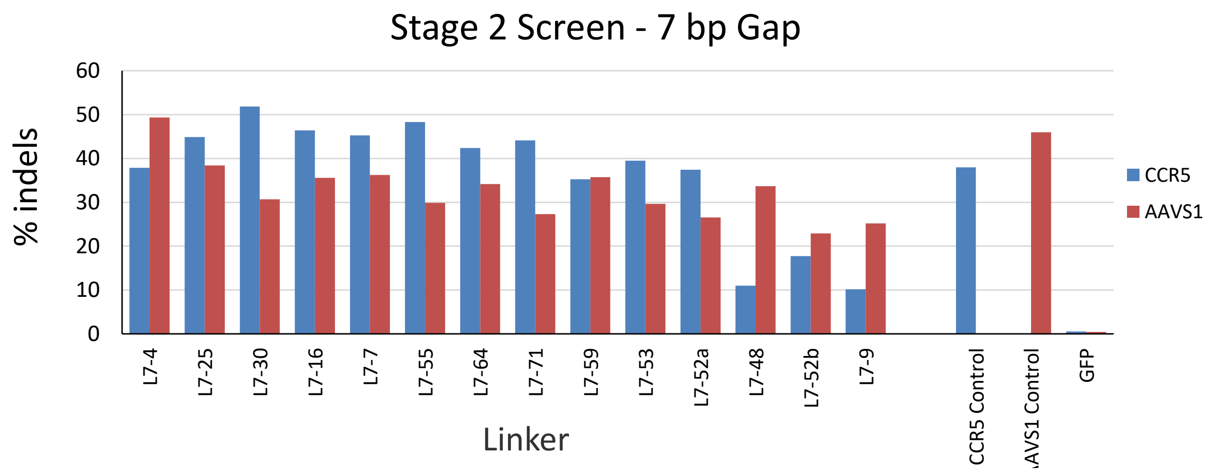

Supplementary Figure 6

Stage 2 screening of selected linkers.

(**a**) Variants of the AAVS1-L ZFN[2] were generated in which the FokI domain was amino-terminally attached to the ZFP via the indicated linker segments. The resultant proteins were then tested in combination with the AAVS1-R ZFN[2] for modification of the NC version of the AAVS1 target bearing a 6 bp gap between ZFP targets that had been engineered into K562 cells. The K562 cell line was nucleofected with plasmid DNA encoding ZFN pairs at a dose of 200ng DNA each ZFN and indels were assessed at each locus by PCR amplification and deep sequencing (MiSeq). Results are provided as red bars. Blue bars show results from the stage 1 screen (see **Supplementary Figure 5**) for comparison. The rightmost bars indicate the %indels generated via the canonical CCR5 and canonical AAVS1 controls as well as the GFP controls. (**b**) As in (**a**), except linkers were screened on a target bearing a 7 bp gap between ZFN binding sites. Each data point represents a single measurement.

a

NC/CN ZFNs with a 6bp Gap - %indels

ZFN Pair	Amino-terminal ZFN harboring Indicated Linker							
	L1	L2	L3	L4	L5	L6	L7	L8
NC/CN-6-1	4.9	7.1	9.3	4.9	3.9	0.3	0.2	0.4
NC/CN-6-2	0.1	0.2	0.3	0.2	0.6	1.1	0.7	0.1
NC/CN-6-3	13.0	9.6	16.9	8.7	5.5	1.4	7.1	2.0
NC/CN-6-4	0.9	2.3	5.7	2.0	0.1	1.5	5.8	3.1
NC/CN-6-5	1.6	1.2	3.5	1.1	0.7	11.5	0.0	0.0
NC/CN-6-6	0.2	0.1	0.1	52.9	63.7	2.3	64.7	58.4
NC/CN-6-7	28.4	nd	32.4	32.3	42.5	39.4	44.6	43.5
NC/CN-6-8	0.2	0.2	0.2	0.1	0.2	0.2	0.2	0.1
NC/CN-6-9	1.5	1.8	2.8	1.8	1.7	2.6	2.9	2.7
NC/CN-6-10	0.3	0.5	0.5	0.5	0.3	0.9	0.7	0.3
average	5.1	2.5	7.2	10.4	11.9	6.1	12.7	11.1

L1: SGSLRGVDPMWH
L2: SGRSPEMDWC
L3: GASLGPPWCP
L4: SGLPMGSYGS
L5: SGQSPGDVGF
L6: SGAIYARPIE
L7: SGAQGSTLDF
L8: SGVKRDSEII

Canonical ZFNs

ZFN Pair	%indels
C1	0.2
C2	10.5
C3	0.8
C4	15.0
C5	0.2
C6	0.3
C7	1.2
C8	21.7
C9	6.1
C10	0.1
average	5.6

b

NC/CN ZFNs with a 7bp Gap - %indels

ZFN Pair	Amino-terminal ZFN harboring Indicated Linker L1	L2	L3	L4	L5	L6	L7	L8
NC/CN-7-1	0.3	3.3	0.7	0.8	0.4	0.6	0.3	0.5
NC/CN-7-2	0.4	0.4	0.4	0.6	0.3	0.6	0.4	0.3
NC/CN-7-3	52.3	36.1	67.4	72.3	49.9	53.6	56.0	57.1
NC/CN-7-4	19.9	27.1	26.5	26.0	22.2	29.7	27.9	39.2
NC/CN-7-5	0.1	0.1	0.1	0.1	0.0	0.1	0.1	0.2
NC/CN-7-6	8.1	9.0	9.3	13.6	8.7	6.8	7.3	11.6
NC/CN-7-7	13.2	20.9	36.4	20.1	19.8	20.1	25.5	37.5
NC/CN-7-8	0.4	1.6	1.6	2.0	0.6	1.3	0.4	3.9
NC/CN-7-9	0.6	3.6	3.4	0.3	2.0	1.6	1.7	4.3
NC/CN-7-10	0.2	0.2	0.3	0.1	0.2	0.2	0.2	0.1
average	9.5	10.2	14.6	13.6	10.4	11.5	12.0	15.5

L1: SQATPTLYYTPL
L2: SGQPMFSWSD
L3: SGERRQSHVL
L4: SGAIRCHDEFWF
L5: SGALQEPWSI
L6: SGFNHSSCDVVY
L7: QSGKIASPHVVI
L8: SGTPHEVGVYTL

Canonical ZFNs

ZFN Pair	%indels
C1	0.2
C2	18.2
C3	2.4
C4	29.3
C5	0.1
C6	0.3
C7	2.3
C8	36.3
C9	12.9
C10	0.2
average	10.2

Supplementary Figure 7

Stage 3 screens of the eight most active linkers for spanning 6- and 7-bp gapped targets.

(**a**) Stage 3 screens of linkers selected for spanning a 6bp gap. A panel of DNA cassettes encoding the eight most active linkers from the stage 2 activity screen (**Supplementary Figure 6**) was cloned into the expression vector containing an amino-terminal FokI ZFN for each of ten new NC/CN ZFN pairs designed for targeting endogenous loci within the human CTLA4 gene. Genes for ten new ZFN pairs bearing the canonical architecture were also designed for screening as a comparison set. All groups were designed such that component ZFPs would exhibit similar levels of affinity and specificity. For each set, 400 ng of plasmid for each ZFN in a pair was transfected into K562 cells and indels for each locus were assessed by PCR amplification and deep sequencing (MiSeq). Linker sequences are shown at the right of each figure and the average modification levels for each linker group are shown at the bottom. (**b**) As in (**a**) except linkers selected for spanning a 7bp gap were tested for cleavage of ZFN dimer targets bearing a corresponding 7 bp gap between monomer targets. Each data point represents a single measurement.

NC/CN ZFNs - 6 bp gap

	%indels		
	Amino-terminal linker		
Pair	N6a	N6b	N6c
NC/CN-6-11	0.5	0.3	0.4
NC/CN-6-12	3.9	8.3	3.9
NC/CN-6-13	4.2	1.1	3.4
NC/CN-6-14	11.9	6.7	8.4
NC/CN-6-15	8.8	8.3	2.6
NC/CN-6-16	0.3	0.1	0.3
NC/CN-6-17	35.4	28.2	37.2
NC/CN-6-18	0.9	0.7	0.8
NC/CN-6-19	28.5	20.0	24.0
NC/CN-6-20	5.6	3.0	3.7
NC/CN-6-21	18.0	10.4	19.2
NC/CN-6-22	90.5	89.6	89.0
NC/CN-6-23	1.3	1.5	1.2
NC/CN-6-24	8.1	6.4	12.4
NC/CN-6-25	17.4	9.7	13.0
NC/CN-6-26	0.7	0.7	0.7
NC/CN-6-27	10.8	6.6	4.9
NC/CN-6-28	13.3	6.0	8.4
NC/CN-6-29	37.3	28.3	36.5
average	15.7	12.4	14.2

NC/CN ZFNs - 7 bp gap

	%indels		
	Amino-terminal linker		
Pair	N7a	N7b	N7c
NC/CN-7-11	35.6	9.4	17.9
NC/CN-7-12	26.0	27.1	28.0
NC/CN-7-13	0.7	0.1	0.2
NC/CN-7-14	0.2	0.1	0.1
NC/CN-7-15	6.2	2.8	2.2
NC/CN-7-16	41.2	53.7	50.2
NC/CN-7-17	nd	nd	nd
NC/CN-7-18	nd	nd	nd
NC/CN-7-19	23.2	27.0	16.4
NC/CN-7-20	3.6	4.9	3.2
NC/CN-7-21	62.8	66.8	60.7
NC/CN-7-22	4.2	2.5	1.4
NC/CN-7-23	5.7	5.0	7.6
NC/CN-7-24	0.4	0.7	2.5
NC/CN-7-25	nd	nd	nd
NC/CN-7-26	2.5	1.9	1.1
NC/CN-7-27	0.4	0.3	0.2
NC/CN-7-28	19.9	16.7	20.7
NC/CN-7-29	nd	nd	nd
average	15.5	14.6	14.2

Canonical ZFNs

Pair	%indels
C11	1.7
C12	0.1
C13	25.8
C14	15.7
C15	14.8
C16	13.4
C17	34.1
C18	1.8
C19	12.2
C20	nd
C21	48.1
C22	13.8
C23	5.8
C24	11.2
C25	1.0
C26	9.7
C27	5.8
C28	8.0
C29	49.8
average	15.1

N6a: SGAQGSTLDF
N6b: SGQSPGDVGF
N6c: SGVKRDSEII

N7a: SGTPHEVGVYTL
N7b: SGERRQSHVL
N7c: SGAIRCHDEFWF

Supplementary Figure 8

Stage 4 screens of dimers bearing the three most active linkers for spanning the 6- and 7-bp gapped targets.

For each spacing and the canonical architecture, 19 new NC/CN ZFNs were designed to an endogenous human locus (intron 1 of the AAVS1 gene). Genes for ZFNs with an amino-terminal FokI were constructed with a panel of the three most active linkers (for their respective spacings) identified in stage 3. All groups were designed such that component ZFPs would exhibit similar levels of affinity and specificity. K562 cells were nucleofected with plasmid DNA at a dose of 400ng DNA each ZFN and indels were assessed at leach locus by PCR amplification and deep sequencing (MiSeq). Linker sequences are shown at the right of the figure and average activities for each linker group are shown at the bottom. Each data point represents a single measurement.

NC CCR5 ZFNs with indicated spacing and linker

6bp Gap

Linker	%indels
N6a	19.6
N6b	21.1
N6c	14.4
$(GGGS)_2$	0.6
$(GGGS)_2G$	1.0
$(GGGS)_2GG$	2.1
$(GGGS)_2GGG$	2.8
$(GGGS)_3$	3.1
$(GGGS)_3G$	2.2
$(GGGS)_3GG$	2.5
GFP	0.5

7bp Gap

Linker	%indels
N7a	23.5
N7b	17.1
N7c	18.9
$(GGGS)_2$	0.5
$(GGGS)_2G$	0.5
$(GGGS)_2GG$	1.2
$(GGGS)_2GGG$	2.1
$(GGGS)_3$	4.1
$(GGGS)_3G$	3.2
$(GGGS)_3GG$	2.8
GFP	0.5

Supplementary Figure 9

Comparison of selected linkers to a panel of flexible linkers in the context of NC CCR5 ZFNs.

The top three linkers for each spacing were evaluated against a panel of simple glycine-rich linkers ranging from 9-14 residues in length. DNA encoding these flexible linkers was cloned into the construct expressing the CCR5-L ZFN. All constructs were paired with CCR5-R and plasmid DNA was nucleofected into the respective K562 cell lines (6 or 7 bp spacing) at a dose of 200 ng of DNA for each ZFN. Indels were monitored at the target locus by PCR amplification followed by deep sequencing (MiSeq). Each data point represents a single measurement.

5bp gap

Pair	%indels Linker N6a	N6b
5-1	nd	nd
5-2	0.1	0.2
5-3	2.4	2.5
5-4	0.2	0.4
5-5	nd	nd
5-6	0.9	0.2
5-7	0.4	0.5
5-8	0.2	0.2
5-9	0.6	0.5
5-10	0.4	0.3
5-11	0.8	0.3
average	0.7	0.6

6bp gap

Pair	%indels Linker N6a	N6b	N7a	N7b
6-1	1.5	1.4	2.2	1.2
6-2	0.5	0.7	0.5	0.3
6-3	2.9	1.2	1.9	1.7
6-4	0.1	0.4	6.9	0.7
6-5	0.4	0.3	nd	0.3
6-6	0.8	0.4	1.7	0.4
6-7	0.4	0.4	1.0	0.4
6-8	0.4	0.4	0.5	0.4
6-9	3.2	0.5	10.7	3.0
6-10	0.1	0.1	0.1	0.1
6-11	0.2	0.2	0.2	0.3
	1.0	0.5	2.6	0.8

N6a: SGAQGSTLDF
N6b: SGQSPGDVGF

N7a: SGTPHEVGVYTL
N7b: SGERRQSHVL

7bp gap

Pair	%indels Linker N6a	N6b	N7a	N7b
7-1	0.1	4.9	0.1	0.7
7-2	35.7	25.1	13.0	11.2
7-3	1.6	0.5	3.9	1.1
7-4	11.5	7.9	5.4	3.1
7-5	16.5	5.4	4.1	1.2
7-6	nd	nd	0.2	12.6
7-7	nd	nd	nd	nd
7-8	12.6	5.0	4.5	4.4
7-9	73.6	76.2	60.4	39.9
7-10	21.2	0.5	0.4	0.8
7-11	1.2	0.7	0.6	0.4
average	19.3	14.0	9.3	7.5

8bp gap

Pair	%indels Linker N6a	N6b	N7a	N7b
8-1	nd	nd	nd	nd
8-2	19.4	4.8	13.5	5.3
8-3	1.0	1.1	99.3	1.0
8-4	18.2	18.1	23.2	17.8
8-5	70.9	71.7	41.5	65.0
8-6	0.8	0.6	0.8	0.3
8-7	nd	nd	nd	nd
8-8	76.6	61.9	79.5	0.5
8-9	47.6	14.5	69.4	43.6
8-10	0.4	0.7	2.2	0.5
8-11	57.1	15.9	56.8	21.8
	32.4	21.0	42.9	17.3

9bp gap

Pair	%indels Linker N7a	N7b
9-1	44.1	1.3
9-2	5.9	2.3
9-3	2.9	2.3
9-4	75.8	2.8
9-5	0.3	0.2
9-6	27.4	0.9
9-7	nd	nd
9-8	23.4	10.1
9-9	13.3	9.2
9-10	0.1	0.6
9-11	0.4	0.3
	19.4	3.0

Supplementary Figure 10

NN architecture screen of dimers with gap sizes of 5-9 bp.

ZFNs with amino-terminal FokI were paired to determine if they could function in an NN architecture. For spacings of 5-9 bp, 11 new ZFN pairs with the NN architecture were designed against an endogenous locus (intron 1 of AAVS1). The pairs for the 5 bp spacing were constructed with the N6a and N6b linkers, the pairs for the 6 to 8 bp spacings were constructed with the N6a, N6b, N7a, and N7b linkers, and the pairs for the 9 bp spacing were constructed with the N7a and N7b linkers. All sets were designed such that component ZFPs would exhibit similar levels of affinity and specificity. DNA encoding ZFN pairs was nucleofected into K562 cells at a dose of 400ng of DNA for each ZFN and target loci were monitored for modification by PCR amplification and deep sequencing (MiSeq). Each data point represents a single measurement.

```
                                          -1      +6
ZFP CCR5-R    F1   MAERPFQCRICMRNFSRSDNLSVHIRTHTGE
              F2      KPFACDICGRKFAQKINLQVHTKIHT(NNS)2-12
              F3      KPFQCRICMRNFSRSDVLSEHIRTHTGE
              F4      KPFACDICGRKFAQRNHRTTHTKIHLRGS

                                          -1      +6
ZFP XSCID-R   F1   MAERPFQCRICMRNFSRSDNLSVHIRTHTGE
              F2      KPFACDICGRKFARNAHRINHTKIHT(NNS)2-12
              F3      KPFQCRICMRNFSRSDDTSEHIRTHTGE
              F4      KPFACDICGRKFAARSTRTNHTKIHLRGS

                                          -1      +6
ZFP XSCID-L   F1   MAERPFQCRICMRNFSRSDTLSEHIRTHTGE
              F2      KPFACDICGRKFAARSTRTTHTKIHT(NNS)2-12
              F3      KPFQCRICMRNFSRSDSLSKHIRTHTGE
              F4      KPFACDICGRKFAQRSNLKVHTKIHLRGS

                                      Recognition Helix      Randomized Linker Library
```

Supplementary Figure 11

Libraries used for selection of base-skipping linkers.

Selections were performed in the context of the ZFP CCR5-R[1] and each ZFP from a previously described ZFN dimer targeted to the human endogenous IL2RG gene (XSCID-L and XSCID-R)[3]. The amino acid sequence for each ZFP is shown in single letter code as an alignment of the four fingers (denoted by F1-F4). The recognition helix for each finger is boxed in orange, with number convention as described[4]. The location and composition of the randomized linker is indicated and shown in red (N = mixture of all bases, S = mixture of G and C).

ZFP	Target Site	Non-Gapped Competitor Site	2-bp Gapped Competitor
CCR5-R	AAACTG<u>d</u>CAAAAG	AAACTGCAAAAG	AAACTG<u>db</u>CAAAAG
XSCID-R	ACTCTG<u>v</u>TGGAAG	ACTCTGTGGAAG	ACTCTG<u>vh</u>TGGAAG
XSCID-L	AAAGCG<u>h</u>GCTCCG	AAAGCGGCTCCG	AAAGCG<u>hd</u>GCTCCG

Supplementary Figure 12

Target sites and competitor sites used for base-skipping linker selections.

The first column shows the name of each host ZFP used in these studies. The second column contains the corresponding target sites used for selection for each host ZFP. Degenerate gap sequences are located between the binding triplets for fingers two and three and are underlined. The third and fourth columns show competitor sites used for counterselection. The complete target sites for these studies were of the form:

TATAAT-[target/competitor site]-<u>TTCACAGTCAGTCCACACGTC</u>

where the sites in the table were inserted into the oligo in the indicated position. The underlined sequence denotes the bases where a complementary oligo was annealed and extended by Klenow to make the double-stranded target. This oligo was either biotinylated at its 5' end to produce the target sites or non-biotinylated to produce the competitor sites.

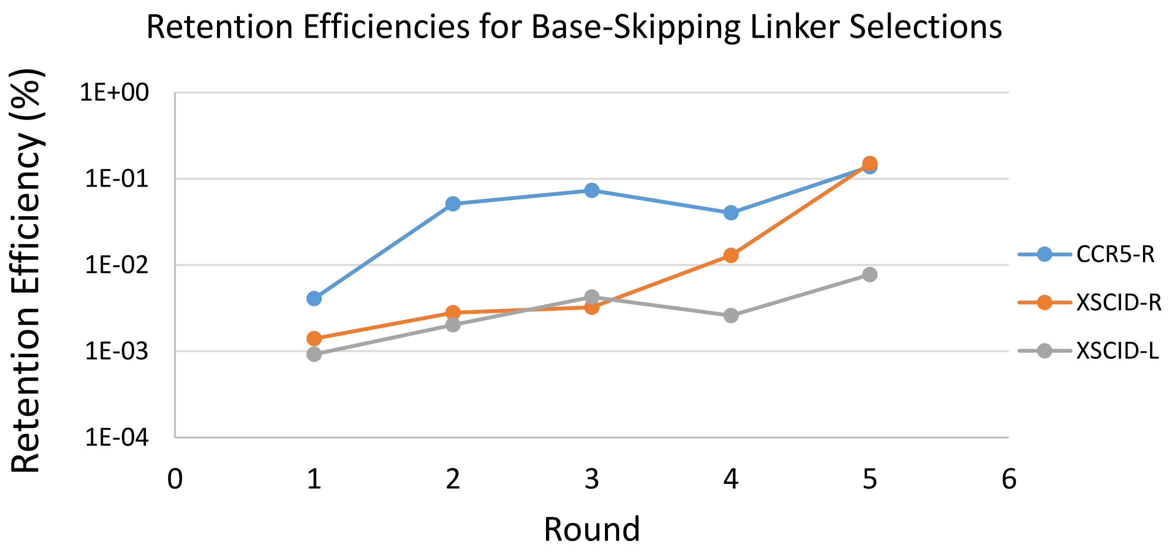

Supplementary Figure 13

Retention efficiencies for base-skipping linker phage selections.

Library construction and phage selections were performed as described in the Supplementary Methods and library sizes obtained were ~5x10^{8}. Selections were completed after five rounds. The retention efficiency was determined at each round via quantification of ampicillin resistant colonies (phagemid selection marker) in the input and output phage pool. Retention efficiency is plotted as a percent (input/output*100) for each round.

a

target	Retention Efficiency (%)		
	CCR5-R	XSCID-R	XSCID-L
0bp gap	0.014	0.009	0.013
1bp gap	0.329	0.241	0.073
2bp gap	0.004	0.001	0.003
no target	0.001	0.001	0.001

b

target	Selectivity - Normalized to 1bp Gap		
	CCR5-R	XSCID-R	XSCID-L
0bp gap	24.0	26.4	5.6
1bp gap	1.0	1.0	1.0
2bp gap	81.8	240.0	27.6
no target	631.6	307.0	53.3

Supplementary Figure 14

Activity and gap discrimination of selected phage pools.

Round five phage pools were assessed for their ability to discriminate between the selection target (bearing a 1bp gap) and alternative targets bearing 0 or 2 bp gaps. A no target negative control was also included. (**a**) Retention efficiencies for each phage pool plated against its target site with the indicated gap spacing between the binding sites for fingers two and three. (**b**) Selectivity of the selected pools normalized to results obtained using a 1-bp gap.

CCR5-R

# clones	Upstream	Randomized linker	Downstream
	H T K I H T	G E	K P F Q C R I C
1	H T K I H T	P D A P K P	K P F Q C R I C
1	H T K I H T	P G L H R P	K P F Q C R I C
2	H T K I H M	E P R A K P P	K P F Q C R I C
1	H T K I H T	P S H T P R P	K P F Q C R I C
1	H T K I H T	G Y S I P R P	K P F Q C R I C
1	H T K I H T	Y P R P I A A	K P F Q C R I C
1	H T K I H T	H P R A P I P	K P F Q C R I C
1	H T K I H T	P N R R P A P	K P F Q C R I C
1	H T K I H T	S P R L P A P	K P F Q C R I C
1	H T K I H T	C P R P P T R	K P F Q C R I C
1	H T K I H T	S S P R S N A	K P F Q C R I C
1	H T K I H T	V S P A P C R S	K P F Q C R I C
1	H T K I H M	P D R P I S T C	K P F Q C R I C

XSCID-R

# clones	Upstream	Randomized linker	Downstream
	H T K I H T	G S Q	K P F Q C R I C
1	H T K I H T	P R P P I P	K P F Q C R I C
3	H T K I H T	Q P R Q I P P	K P F Q C R I C
3	H T K I H T	P N R C P P T	K P F Q C R I C
2	H T K I H T	Y P R P L L A	K P F Q C R I C
10	H T K I H T	P L C Q R P M K Q	K P F Q C R I C
1	H T K I H T	P L C Q R P M K Q	K P F Q C R I C

XSCID-L

# clones	Upstream	Randomized linker	Downstream
	H T K I H T	G S Q	K P F Q C R I C
1	H T K I H T	G L P K P	K P F Q C R I C
3	H T K I H T	S R P R P	K P F Q C R I C
1	H T K I H T	L P L P R P	K P F Q C R I C
4	H T K I H T	V P R P T P P	K P F Q C R I C
1	H T K I H T	L P P C F R P	K P F Q C R I C
1	H T K I H T	L P P C F R P	K P F Q C R I C
1	H T K I H T	K H G T P K H R E D	K P F Q C R I C

Supplementary Figure 15

Sequences of selected base-skipping linkers.

Amino acid sequences of the linker region obtained following five rounds of selection. The randomized portion of the linker is bounded by vertical black lines. The sequence upstream of

the linker comprises carboxy-terminal residues of finger two with the two zinc-coordinating histidines underlined and the downstream sequence comprises amino-terminal residues of finger three with the two zinc-coordinating cysteines underlined. The parent linker sequence for each host ZFP is shown at the top of each alignment. Proline residues are highlighted in orange and basic residues are highlighted in blue. The number of clones obtained for each sequence is listed at the left. Residues shaded in grey represent additional mutations that may have been generated during selection.

a

Sequence of the center linker	Linker origin	ELISA score for CCR5-R with indicated center linker binding to target below. Gap sequence indicated by "X". AAACTG(X)CAAAAG [score is normalized to CCR5-R bound to its non-gapped target (entry in blue)]								
		0-bp gap	1-bp gap					2-bp gap		
		-	A	C	G	T	average ratio of 1bp:0bp score	TC	AC	TG
TGEKP	Perez[1] (parent)	1.00	.01	.01	.03	.01	.02	.002	.002	.003
TPDAPKPKP	Selection	.02	.16	.13	.68	.95	23.75	.01	.003	.01
TPGLHRPKP	Selection	.04	.19	.1	.65	.81	10.94	.01	.004	.01
TEPRAKPPKP	Selection	.01	.39	.17	.78	.93	70.72	.02	.01	.01
TPSHTPRPKP	Selection	.02	.3	.13	.84	.8	25.1	.02	.01	.01
TGYSIPRPKP	Selection	.01	.13	.06	.43	.55	44.57	.01	.005	.01
TYPRPIAAKP (designated 1f)	Selection	.01	.41	.14	.65	.64	82.25	.01	.004	.01
THPRAPIPKP (designated 1c)	Selection	.004	.2	.09	.57	.6	78.86	.01	.004	.004
TPNRRPAPKP (designated 1d)	Selection	.004	.23	.09	.52	.52	90.27	.01	.01	.01
TSPRLPAPKP	Selection	.01	.26	.14	.62	.81	67.95	.01	.005	.01
TCPRPPTRKP	Selection	.005	.18	.05	.48	.62	70.16	.01	.004	.01
TSSPRSNAKP	Selection	.01	.05	.02	.2	.25	20.85	.01	.003	.01
TVSPAPCRSKP	Selection	.01	.03	.01	.14	.19	11.52	.02	.005	.01
TPDRPISTCKP	Selection	.01	.11	.05	.29	.47	15.41	.03	.01	.02
TGGGGSQKP	Flexible linker	.0007	.0003	.0007	.001	.001	1.2	.0006	.002	.001
LRQKDERP	Kim and Pabo[5]	.01	.01	.01	.04	.08	3.49	.003	.002	.004
TGEGGKP	Sugiura[6]	.1	.003	.025	.03	.03	.15	.002	.002	.003

b

Sequence of the center linker	Linker origin	ELISA score for XSCID-R with indicated center linker binding to target below. Gap sequence indicated by "X". ACTCTG(X)TGGAAG [score is normalized to XSCID-R bound to its non-gapped target (entry in blue)]								
		0-bp gap	1-bp gap					2-bp gap		
		-	A	C	G	T	average ratio of 1bp:0bp score	AC	AT	CT
TGSQKP	Urnov[3] (parent)	1.00	.01	.01	.03	.02	.02	.01	.01	.004
TPRPPIPKP	Selection	.14	.97	.67	1.85	2.09	10.2	.02	.01	.01
TQRPQIPPKP	Selection	.15	1.66	1.	2.86	3.05	14.68	.03	.02	.01
TPNRCPPTKP	Selection	.31	1.68	1.13	2.62	3.16	7.53	.03	.02	.01
TYPRPLLAKP	Selection	.29	1.95	1.27	3.88	3.97	10.08	.03	.01	.01
TPLCQRPMKQKP	Selection	.28	1.82	1.28	3.44	4.	10.88	.08	.05	.02
TGGGGSQKP	Flexible linker	.51	.06	.05	.41	.39	.44	.01	.01	.02
LRQKDERP	Kim and Pabo[5]	.25	.03	.02	.18	.13	.36	.01	.01	.01
TGEGGKP	Suigiura[6]	1.3	.02	.02	.05	.04	.03	.01	.01	.01

c

Sequence of the center linker	Linker origin	ELISA score for XSCID-L with indicated center linker binding to target below. Gap sequence indicated by "X". AAAGCG(X)GCTCCG [score is normalized to XSCID-L bound to its non-gapped target (entry in blue)]								
		0-bp gap	1-bp gap					2-bp gap		
		-	A	C	G	T	average ratio of 1bp:0bp score	TT	TA	CT
TGSQKP	Urnov[3] (parent)	1.00	.003	.003	.003	.003	.003	.001	.008	.002
TGLPKPKP	Selection	.14	.19	.11	.38	.86	2.84	.03	.02	.02
TSRPRPKP	Selection	.18	.52	.22	.77	2.07	4.93	.03	.03	.03
TLPLPRPKP	Selection	.25	.58	.25	.85	1.36	3.01	.04	.03	.03
TVPRPTPPKP (designated 1e)	Selection	.16	2.35	1.02	1.58	2.55	11.71	.05	.05	.06
TLPPCFRPKP	Selection	.36	.72	.25	.77	2.72	3.11	.06	.06	.05
TKHGTPKHREDKP	Selection	.01	.01	.01	.01	.01	.79	.002	.002	.002
TGGGGSQKP	Flexible linker	.46	.07	.04	.08	.17	.19	.03	.03	.07
LRQKDERP	Kim and Pabo[5]	.26	.05	.03	.06	.1	.22	.02	.02	.02
TGEGGKP	Sugiura[6]	1.39	.02	.03	.05	.08	.03	.03	.02	.03

Supplementary Figure 16

ELISA characterization of host ZFPs harboring selected linkers.

(**a**) ELISA of linkers in the context of the CCR5-R host ZFP[1]. Tested targets are summarized at top and included the parental binding site and variants that contained a one or two bp insertion at the location of the "X" as shown. Tested linkers are listed at left and included the parental, non-skipping linker (top entry), 13 linkers identified via phage selections (entries labelled "selection") and three control linkers at bottom, consisting either of designs from prior studies (LRQKDERP (Kim and Pabo[5]); TGEGGKP (Sugiura[6])) or a simple poly-glycine tether. Note that the amino acid sequences for the linkers selected in these studies contain an amino-terminal threonine and a carboxy-terminal lysine-proline which were held constant in the selections (see **Supplementary Figures 11** and **15**). The ELISA was performed essentially as described[7], with values normalized to CCR5-R binding to its non-gapped target site (blue entry). The column with the color scale indicates the ratio of the average normalized ELISA score for the 1-bp gapped targets over the normalized ELISA score for the non-gapped target site. (**b**) Same as in (**a**) but for the host ZFP XSCID-R[3]. (**c**) Same as in (**a**) but for the host ZFP XSCID-L[3]. Each data point represents the average of duplicate measurements.

a

One 1bp-skipping Linker per Pair

	%indels	
	Linker	
ZFN Pair	1c	LRQKDERP
Skip-1	31.2	5.4
Skip-2	1.0	0.4
Skip-3	0.3	0.2
Skip-4	24.7	27.1
Skip-5	29.4	0.7
Skip-6	2.0	1.1
Skip-7	47.0	29.2
Skip-8	1.0	1.2
Skip-9	0.5	0.4
Skip-10	65.5	62.6
average	20.3	12.8

b

Canonical ZFN - No Skipping Linkers

ZFN Pair	%indels
NoSkip-1	5.5
NoSkip-2	0.3
NoSkip-3	34.4
NoSkip-4	28.5
NoSkip-5	64.4
NoSkip-6	16.1
NoSkip-7	10.0
NoSkip-8	7.6
NoSkip-9	5.2
NoSkip-10	1.8
average	17.4

Supplementary Figure 17

Comparison of ZFN dimers containing a single base-skipping linker and canonical ZFNs.

(**a**) Target modification levels achieved by a panel of 10 ZFN dimers, each bearing one ZFN that spanned a skipped base using either the 1c linker (developed in this study) or the control linker that was most selective for a 1-bp gap from the ELISA study (LRQKDERP)[5]. ZFNs were targeted to loci within intron 1 of the human AAVS1 gene and screened for activity via nucleofection of plasmid DNA into K562 cells at a dose of 400 ng of DNA per ZFN. Genomic DNA was isolated and target loci were PCR amplified and assessed for modification by deep sequencing (MiSeq). (**b**) Modification levels achieved by a parallel set of 10 all-canonical ZFN pairs (i.e. no base skipping) designed for the same target region. Each data point represents a single measurement.

LCA10 mutation

61029

TGAGCCACCGCACCTGGCCCCAGTTGTAATTGTGA**A**TATCTCATACCTATCCCTATTGGCAGTGTCTTAGTTTTA

ACTCGGTGGCGTGGACCGGGGTCAACATTAACACT**T**ATAGAGTATGGATAGGGATAACCGTCACAGAATCAAAAT

61033

CEP290

Supplementary Figure 18

Binding sites for the ZFN dimer targeting *CEP290*.

A segment of the *CEP290* gene that bears the LCA10 mutation (highlighted in blue) is shown. An NN ZFN dimer was identified that straddled this mutation and induced 85% indels in K562 cells (**Fig. 4b**). The binding sites for the indicated ZFNs are boxed in red. Amino acid and DNA sequences for these ZFNs are provided in **Supplementary Tables 2** and **3**.

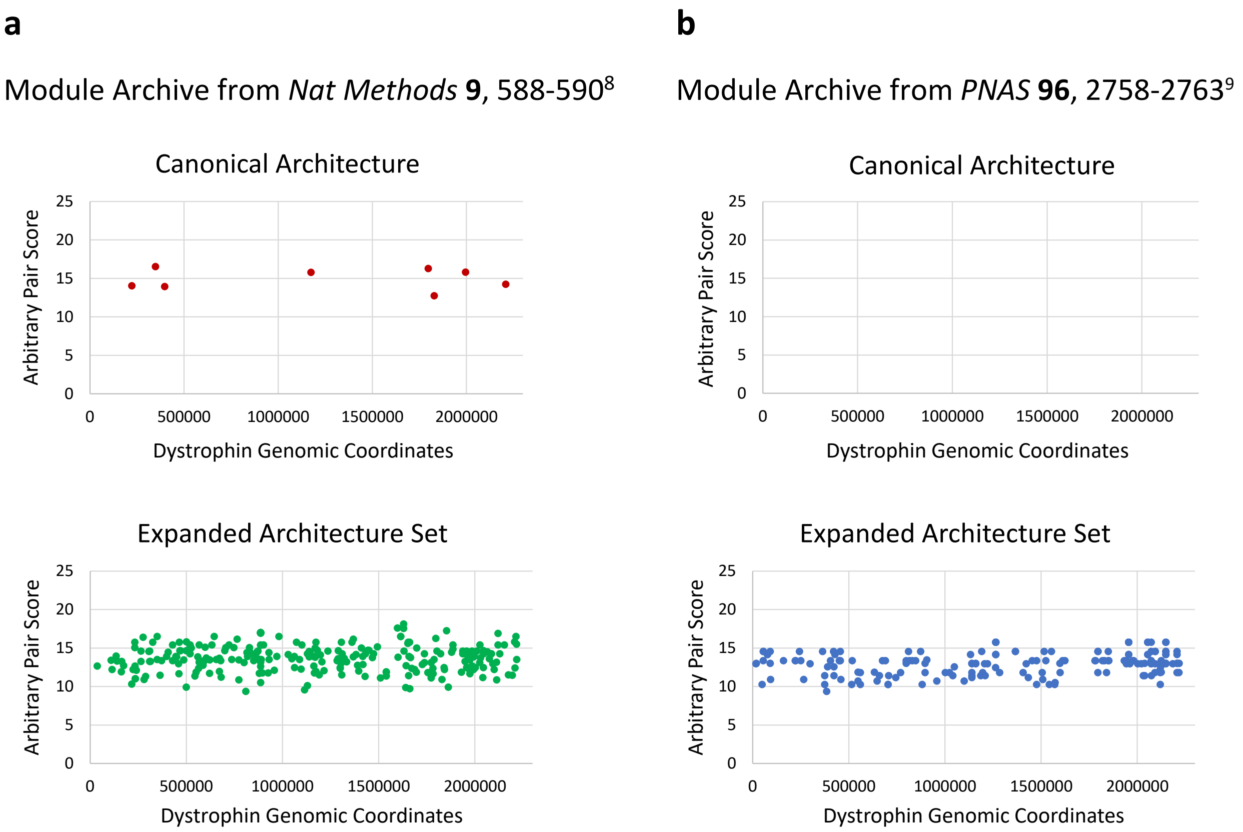

Supplementary Figure 19

Impact of new linkers and architectures on the targeting capabilities of other ZFP design systems.

Zinc fingers and associated target subsites from two well-established alternative finger sets[8, 9] were used to search the human dystrophin gene for potential ZFN targets. This assessment searched for dimers bearing 5-6 fingers per ZFN, conforming either to the standard canonical configuration (top panels), or to the expanded set of configurations enabled by our new linkers and amino-terminal FokI (bottom panels). For plotting purposes, each ZFN pair was given an arbitrarily generated "score" in order to spread out data in the y dimension, to reduce overlap. (**a**) Results obtained using the zinc finger units described in Reference 8, showing a >33-fold improvement in the number of candidate dimer designs, from 8 to 267 unique cleavage sites. (**b**) Results obtained using the GNN-targeted fingers described in Reference 9. Although this finger set yields no design options in the context of the canonical architecture, 179 unique cleavage sites are identified when it is applied in combination with our new linkers and architectures.

a

Module Archive from *Nat Methods* **9**, 588-590[8]

Pair	Architecture	# base-skipping linkers (Left:Right)	% indels - ZFN treated Rep 1	Rep 2	Rep 3	Average indels	SD	GFP
1	NC	0:0	1.4	1.3	0.8	1.2	0.3	0.06
2	NC	0:1	nd	nd	nd	nd	nd	nd
3	CC	1:0	1.3	1.9	1.6	1.6	0.2	0.03
4	CC	1:0	32.9	34.9	33.5	33.8	0.9	0.03
5	NC	0:0	0.1	0.2	0.2	.2	0.0	0.04
6	CN	0:0	0.1	0.1	0.1	.1	0.0	0.09
7	NC	0:0	0.2	0.3	0.8	.4	0.3	0.09
8	NN	0:1	37.3	36.3	41.2	38.3	2.1	0.07
9	CN	0:1	8.6	10.2	7.8	8.9	1.0	0.18
10	NN	0:0	14.7	13.5	15.2	14.5	0.7	0.04
11	CN	0:0	3.7	3.6	4.5	4.0	0.4	0.07
12	NN	1:0	59.6	63.1	65.9	62.8	2.6	0.08
13	CC	1:0	75.6	75.5	79.2	76.8	1.7	0.07
14	NN	0:1	34.6	33.7	33.9	34.1	0.4	0.08
15	CC	1:0	55.8	51.8	62.3	56.6	4.3	0.11

b

Module Archive from *PNAS* **96**, 2758-2763[9]

Pair	Architecture	# base-skipping linkers (Left:Right)	% indels - ZFN treated Rep 1	Rep 2	Rep 3	Average indels	SD	GFP
1	NC	1:0	17.8	16.4	19.4	17.9	1.2	0.08
2	NN	1:1	0.5	0.5	0.4	.5	0.1	0.06
3	NC	1:1	27.9	26.7	26.0	26.9	0.8	0.07
4	NC	0:1	67.4	64.5	65.9	65.9	1.2	0.11
5	CC	0:1	4.3	4.2	3.5	4.0	0.4	0.05
6	CN	0:0	16.8	13.6	12.4	14.3	1.8	0.04
7	NN	0:0	0.6	0.5	0.6	.6	0.1	0.14
8	NC	0:1	24.4	22.7	27.5	24.9	2.0	0.06
9	NN	0:1	73.6	76.3	72.3	74.0	1.6	0.16
10	NC	1:0	0.0	0.0	0.0	.0	0.0	0.06
11	CC	1:0	56.0	56.8	54.8	55.8	0.8	0.07
12	CC	1:0	67.0	67.4	62.6	65.7	2.2	0.08
13	NN	0:1	11.4	11.7	8.3	10.5	1.5	0.15
14	CN	0:0	nd	nd	nd	nd	nd	nd
15	CN	0:0	nd	nd	nd	nd	nd	nd

Supplementary Figure 20

ZFNs using finger designs from alternative ZFP design systems are active in mammalian cells.

Genes for a subset of 15 ZFN dimers from two alternative archives[8, 9] (see **Supplementary Figure 19**) were chosen to be constructed for testing in mammalian cells. Each dimer was chosen to

contain at least one design feature developed in these studies. Helices were first transferred into our 1- and 2-finger module backbones which contained the following amino acid sequences:

2-finger module: FQCRICMRNFSXXXXXXXHIRTHTGEKPFACDICGRKFAXXXXXXXHTKIH
1-finger module: FQCRICMRKFAXXXXXXXHTKIH

where the underlined X sequences represent the recognition helices.

Genes encoding the ZFNs were then cloned into an expression vector, mRNA was produced, and mRNA for each ZFN pair was nucleofected into K562 cells at a dose of 400ng mRNA per ZFN. Indels at each locus were assessed by PCR amplification followed by deep sequencing (MiSeq). Each pair was transfected in triplicate. (**a**) Results obtained for the ZFNs designed from the module archive described in Reference 8. Shown are the pair name, architecture type, number of base-skipping linkers in each ZFN, measurements of indels obtained from three biological replicates, the average % indels from the three replicates, standard deviations for the three replicates, and the % indels for GFP-treated cells. The data shown for each replicate represents a single measurement. A single GFP transfection was used for background correction of the ZFN-treated samples where sequences common to both the ZFN-treated and GFP-treated sequencing reads were removed prior to quantification. The GFP value reported for each pair is the average of the GFP values obtained from the three replicates. Samples marked with "nd" either failed PCR amplification or failed to satisfy MiSeq quality metrics. (**b**) Same as (**a**) except the results are for ZFNs designed from the module archive described in Reference 9. Design information for these ZFN pairs is shown in **Supplementary Table 4**.

Exon c1

aTATCCAGAACCCTGACCCTGCCGTGTACCAGCTGAGAGACTCTAAATCCAGTGACAAGTCTGTCTGCCTATTCA
CCGATTTTGATTCTCAAACAAATGTGTCACAAAGTAAGGATTCTGATGTGTATATCACAGACAAAACTGTGCTAG
ACATGAGGTCTATGGACTTCAAGAGCAACAGTGCTGTGGCCTGGAGCAACAAATCTGACTTTGCATGTGCAAACG
CCTTCAACAACAGCATTATTCCAGAAGACACCTTCTTCCCCAGCCCA

Exon c2

GAAAGTTCCTGTGATGTCAAGCTGGTCGAGAAAAGCTTTGAAACAGgtaagacaggggtctagcc

Exon c3

ATACGAACCTAAACTTTCAAAACCTGTCAGTGATTGGGTTCCGAATCCTCCTCCTGAAAGTGGCCGGGTTTAATC
TGCTCATGACGCTGCGGCTGTGGTCCAGCTGA

Site	Architecture	# of base-skipping linkers (Left ZFN:Right ZFN)
1	Canonical	1:1
2	Canonical	0:1
3	NC	1:1
4	NC	1:0
5	Canonical	0:0

Supplementary Figure 21

Target sites for TRAC ZFNs.

DNA sequence for the three exons of T-cell receptor alpha constant region that were targeted for the studies shown in **Fig. 5**. The black upper-case bases are the exonic sequence and the bases shown in lower-case gray are intronic sequence. ZFN sites are indicated by the colored boxes and are coded according to the table at the bottom. The table also contains the architecture type and number of base-skipping linkers in each ZFN.

a **b**

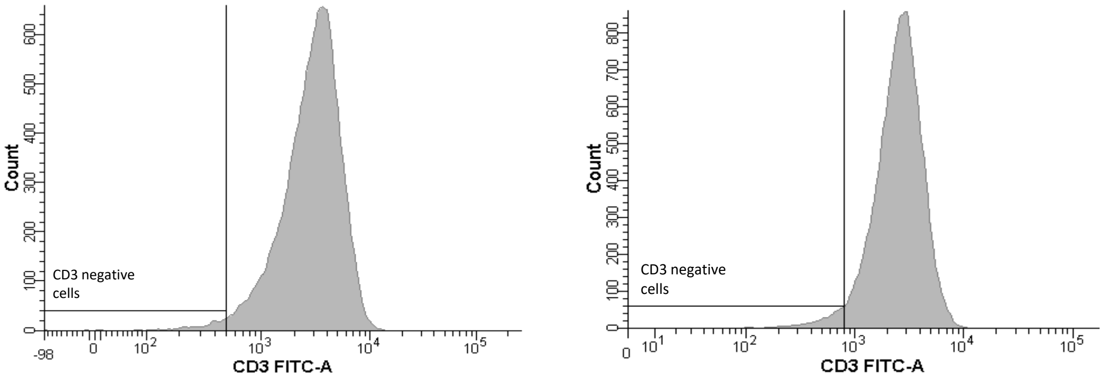

Supplementary Figure 22

Gating strategy for mock transfected T-cells from TRAC flow cytometry studies.

Plots are shown for the mock transfected T-cells used as negative controls for the flow cytometry analysis of ZFN-treated T-cells (**Fig. 5**). CD3 negative cells are gated at the left. The plot for mock transfected T-cells corresponding to the studies with TRAC 1, TRAC 2, and TRAC 3 is shown in (**a**) whereas the plot for mock transfected T-cells corresponding to the studies with TRAC 4 and TRAC 5 is shown in (**b**).

a

TRAC 1 (55204:53759)

Canonical ZFN; 500ng RNA each ZFN

				ZFN-treated		GFP-treated		
Locus	UID	Location	# Capture Events	# Sequences	%indels	# Sequences	%indels	p val if <0.05
TRAC 1	HZDFWLFM	chr14:22547662	5608	54990	81.60	53531	0.15	< .001
1-1	FYCPKCBQ	chr20:34112194	275	67603	0.40	9383	0.01	< .001
1-2	SYYXXPBJ	chr21:34950682	219	66996	0.04	32176	0.03	
1-3	ZVHETYAT	chr2:200751518	186	91155	0.01	38318	0.04	
1-4	QLPTTBWF	chr15:90295624	126	59684	0.08	22348	0.09	
1-5	EYMRKSYT	chr8:78155158	86	41177	0.70	21612	0.03	< .001
1-6	MNFJVJZX	chr15:75786838	85	67527	0.04	21915	0.06	
1-7	EVCWJRIS	chr9:128257174	79	41881	0.06	14053	0.09	
1-8	PLFFZNDT	chr15:75163554	67	48604	0.03	20548	0.09	
1-9	QEJFBHYY	chr22:19741248	62	72050	0.08	23069	0.03	0.03
1-10	XBQLPJBA	chr1:31595900	60	67090	0.08	29154	0.07	
1-11	EPWJHARP	chr2:188294036	60	78784	0.05	31302	0.05	
1-12	FDEMAJYS	chr12:7907396	57	14104	0.25	3272	0.52	
1-13	PXAEWDKF	chr12:57745790	51	52925	0.04	25486	0.04	
1-14	PAZWAIIW	chr12:121302840	49	nd	nd	nd	nd	
1-15	RNLCRFAE	chr1:12434446	44	81952	0.05	36260	0.02	
1-16	AVQJAYMW	chr1:51934140	42	61982	0.03	27988	0.08	
1-17	HFALYSQB	chr1:200969740	39	56707	0.05	26214	0.04	
1-18	MBVYNCER	chr6:13566140	39	70474	0.02	34075	0.04	
1-19	AKECRVWC	chr2:239998960	37	71645	0.03	24538	0.01	
1-20	JDTCQYKM	chr19:47348938	35	61870	0.04	25497	0.06	
1-21	FXCEFHWS	chrX:109959670	35	65896	0.02	36667	0.07	
1-22	NQENBNMY	chr14:53202806	34	73358	0.05	23364	0.18	
1-23	VCNEKCNH	chr19:55115746	34	73399	2.32	25899	1.04	< .001
(1)	(2)	(3)	(4)	(5)	(6)	(7)	(8)	(9)

b

TRAC 2 (55229:53785)

Canonical ZFN; 62.5ng RNA left ZFN + 250ng RNA right ZFN

				ZFN-treated		GFP-treated		
Locus	UID	Location	# Capture Events	# Sequences	%indels	# Sequences	%indels	p val if <0.05
TRAC 2	ESJMZPQX	chr14:22547668	12481	55709	81.70	52165	0.11	< .001
2-1	ARWQNIFR	chr10:48510024	6226	62976	1.18	31263	0.05	< .001
2-2	EEPFXJML	chr14:74911452	376	60631	0.52	29498	0.05	< .001
2-3	ZHBFQEER	chr7:29537680	276	55445	0.05	31302	0.04	
2-4	PFLHFIRW	chr5:134459526	246	8279	0.18	299	0.00*	.006
2-5	ZLHXRNPR	chr3:143018336	200	54514	0.12	31320	0.07	
2-6	ICXEKVQV	chr11:68896870	176	36421	0.09	13986	0.06	
2-7	VQJWWBVS	chr12:2298892	144	62695	0.19	28557	0.03	< .001
2-8	QDRCEHWQ	chr7:139562590	114	44432	0.26	15386	0.03	< .001
2-9	BQLQNFBV	chr16:84138864	114	65169	0.07	28401	0.04	
2-10	LPDALARA	chr14:27506720	112	47499	0.31	15732	0.10	< .001
2-11	YFRXKNKX	chr17:80188358	77	56068	0.05	18953	0.07	
2-12	JEQVZMIS	chr4:26571334	70	43640	0.03	21482	0.05	
2-13	ISINLHPL	chr17:36523288	58	46474	0.10	17927	0.04	
2-14	JDBBAMSV	chr6:148546118	51	68033	0.03	31008	0.08	
2-15	EHEVERHV	chr18:7456644	42	47223	0.10	26079	0.03	0.02
2-16	EMAVLRCE	chr9:120508578	38	46792	0.14	14102	0.08	
2-17	BSWIPATZ	chr1:161160046	34	50445	0.03	17933	0.03	
2-18	IJMAARTS	chr14:22546254	33	51749	0.02	20171	0.03	
2-19	HIXMVQZS	chr14:22546578	33	50149	0.02	32930	0.03	
2-20	RSKKEWZL	chr20:47196552	32	49917	0.03	25185	0.06	
2-21	KLNSIFIF	chr7:2636570	31	56121	0.04	15795	0.09	
2-22	NFCCVPTP	chr17:55642036	30	72083	0.19	36220	0.03	< .001
2-23	VPDPXMQZ	chr18:70127526	29	58442	0.04	16639	0.05	
(1)	(2)	(3)	(4)	(5)	(6)	(7)	(8)	(9)

c

TRAC 3 (55266:53853)

NC ZFN; 2000ng RNA left ZFN + 500ng RNA right ZFN

				ZFN-treated		GFP-treated		
Locus	UID	Location	# Capture Events	# Sequences	%indels	# Sequences	%indels	p val if <0.05
TRAC 3	HSAEXPMF	chr14:22549654	4195	35489	85.14	47182	0.37	< .001
3-1	FIBBPCQN	chr2:10072792	67	50361	0.04	25632	0.04	
3-2	VQYATIBV	chr8:143573222	57	16399	0.02	14469	0.20	
3-3	ZFKQFRTT	chr21:25838562	52	41391	0.18	28354	0.02	< .001
3-4	CIBSSEQD	chr3:185380610	31	41945	0.03	30102	0.05	
3-5	YTHJHJBF	chr3:113729280	24	48310	0.04	24341	0.09	
3-6	TCASMMDR	chr2:127183006	22	40512	0.08	15774	0.06	
3-7	AXJVTBWL	chr3:129120704	22	32907	0.09	24372	0.03	
3-8	WRAKIDZW	chr10:24893938	21	44472	0.04	24199	0.05	
3-9	CZVNWSLD	chr8:80297308	19	43928	0.64	30344	0.04	< .001
3-10	TFTRHMIQ	chr17:6710504	19	47438	0.03	21564	0.05	
3-11	BSKNLJTJ	chr16:14135746	18	74231	0.08	23890	0.03	.003
3-12	PXEZQHDY	chr13:111389124	17	38177	1.60	25486	0.06	< .001
3-13	ZBENRPIA	chr15:66504272	17	53526	0.03	34365	0.02	
3-14	FLRRWIKF	chr17:30104742	17	65765	0.04	30640	0.07	
3-15	LIFIFCHV	chr2:135447360	16	36694	0.02	31038	0.04	
3-16	EEMCLQEA	chr17:62774642	15	34515	0.02	19861	0.12	
3-17	RLVWNFIM	chr10:48510022	14	48749	0.07	21216	0.46	
3-18	NCAQQDQH	chr14:22548650	14	nd	nd	nd	nd	
3-19	NZLCVDQB	chr1:213562812	13	31658	0.03	18801	0.05	
3-20	MRQDNQJW	chr14:96762826	13	42875	0.05	27665	0.04	
3-21	EDQYPRCA	chr20:61650064	13	51743	0.02	18767	0.04	
3-22	JEXMSYAK	chrX:3550018	13	10390	0.21	9369	0.13	
3-23	QLKPCDBI	chrX:100668172	13	61015	0.03	19695	0.05	
(1)	(2)	(3)	(4)	(5)	(6)	(7)	(8)	(9)

d

TRAC 4 (53885:53909)

NC ZFN; 62.5ng RNA left ZFN + 250ng RNA right ZFN

				ZFN-treated		GFP-treated		
Locus	UID	Location	# Capture Events	# Sequences	%indels	# Sequences	%indels	p val if <0.05
TRAC 4	XDJSREYN	chr14:22550580	6647	46406	82.04	25430	0.44	< .001
4-1	EYPPINVY	chr3:19931440	105	47639	0.04	23466	0.06	
4-2	YFLXFLEL	chr16:85765876	87	35714	0.13	5059	0.06	
4-3	KXBKZYII	chr22:40240116	54	30125	0.06	16196	0.07	
4-4	TKCJYLIC	chr17:44006728	33	53952	0.05	9046	0.03	
4-5	ZDYEECBT	chr14:22549656	31	39898	0.07	24858	0.06	
4-6	SLMKESIE	chr12:48296788	27	44954	0.03	32365	0.05	
4-7	HPLYAWKH	chr22:42745540	21	46691	0.03	27625	0.08	
4-8	MLQJFDZP	chr7:106290470	20	34043	0.02	25593	0.13	
4-9	BVSCLNHN	chr1:207819098	19	41932	0.03	20015	0.05	
4-10	NQHEXJEJ	chr6:20550368	19	28926	0.02	25350	0.08	
4-11	KSPYJTWS	chr14:22972708	19	39350	0.02	26263	0.05	
4-12	JERXSCJI	chr19:14384458	19	34412	0.04	21353	0.04	
4-13	SYHTNLXT	chr15:41543978	18	48207	0.04	22648	0.05	
4-14	ANVIHSJX	chr19:1872612	18	49343	0.03	22962	0.02	
4-15	RERBADFH	chr18:51458162	16	35094	0.04	34051	0.08	
4-16	SJHTKYLF	chr19:58546180	16	35540	0.04	28379	0.08	
4-17	MPMZNCVY	chr14:22550180	15	41879	0.06	23175	0.06	
4-18	JLMCTKJK	chr22:16602834	14	44420	0.06	20683	0.06	
4-19	RZSJCREH	chr1:21195292	13	59521	0.02	33375	0.05	
4-20	DHBIBHBZ	chr7:152434960	13	38888	0.07	13088	0.12	
4-21	ERWEMHKL	chr8:129726418	12	36371	0.05	28085	0.10	
4-22	LWVDDXCE	chr18:268500	12	5431	0.04	29111	0.11	
4-23	FMWAJQFA	chr1:37807640	11	23657	0.07	16586	0.10	
4-24	KKWBCEDB	chr3:8426808	11	56631	0.04	37465	0.03	
4-25	RTCJPHHS	chr7:134661772	11	39558	0.04	27788	0.06	
4-26	ANZPESKW	chr22:18563448	11	41221	0.00	32601	0.06	
(1)	(2)	(3)	(4)	(5)	(6)	(7)	(8)	(9)

e

TRAC 5 (55248:55254)

Canonical ZFN; 125ng RNA each ZFN

				ZFN-treated		GFP-treated		
Locus	UID	Location	# Capture Events	# Sequences	%indels	# Sequences	%indels	p val if <0.05
TRAC 5	VWRYIQVY	chr14:22550584	8595	75037	79.37	31338	0.27	< .001
5-1	BTFKQLCV	chr2:102757208	55	48254	0.02	34352	0.08	
5-2	HXPQPEQN	chr10:69524172	37	63741	0.03	32930	0.03	
5-3	MAEZYIBI	chr1:14638544	26	13501	0.23	11282	0.51	
5-4	LWAKIJLT	chr14:22549762	20	49081	0.02	31042	0.05	
5-5	PWIPQEKM	chr2:55427470	18	248	1.21	39866	0.53	
5-6	ZHEKZLXP	chr9:127793076	18	26936	0.04	26163	0.08	
5-7	DJMICCJL	chr9:13565882	17	32614	0.08	21095	0.08	
5-8	PQRFJSPA	chr14:85441204	16	6544	0.18	3273	0.64	
5-9	VPTIRIDQ	chr1:46514082	15	77557	0.01	37542	0.04	
5-10	SBSVEXYN	chr19:14202298	15	51938	0.23	21957	0.33	
5-11	VCEJBZDT	chrX:23799200	15	54452	0.05	20736	0.12	
5-12	EIBAXZYK	chr1:216854328	14	nd	nd	nd	nd	
5-13	FTKMKSSV	chr4:70823134	14	22071	0.10	4032	0.82	
5-14	MWHVLRNQ	chr10:23114036	14	35003	0.05	13051	0.06	
5-15	MYZCKDLJ	chr10:28771380	14	9056	0.04	26433	0.10	
5-16	RLZXIYAM	chr18:72313220	14	31031	0.04	25897	0.09	
5-17	WEBNWTPA	chr6:48877832	13	23204	0.03	22288	0.28	
5-18	VKMXNBHD	chr14:22550900	13	43620	0.02	36081	0.06	
5-19	ZFKLJPQA	chr1:93333506	12	28886	0.02	36861	0.05	
5-20	ZIPSYCJY	chr4:78345662	12	36060	0.02	34747	0.07	
5-21	THXKXXAN	chr5:180298320	12	30389	0.08	24647	0.06	
5-22	QMXAVSAS	chr9:122350678	12	33477	0.04	26694	0.10	
5-23	DVPHRCEP	chr9:128878038	12	43073	0.02	28335	0.08	
5-24	CCJJVJKJ	chr10:59689940	12	41516	0.05	28710	0.02	
5-25	IZMLZEVE	chr11:94681328	12	53879	0.02	27304	0.03	
5-26	VMTPPDSP	chr16:74830352	12	36592	0.04	21798	0.11	
5-27	VNRWNTVA	chrX:114136666	12	17257	0.19	16095	0.10	
(1)	(2)	(3)	(4)	(5)	(6)	(7)	(8)	(9)

Supplementary Figure 23

Off-target analysis of T-cells treated with five distinct TRAC-targeted ZFN dimers.

Summary of the off-target analysis performed on T-cells treated with a high dose of the five ZFN pairs targeted to the TRAC gene. At the top of each panel is the site that is targeted and the SBSID (unique identifier) for the two ZFNs in the pair (left:right ZFN). Below this is annotated the type of ZFN architecture and the dose of each of the ZFNs. The table contains the following information: (1) Locus ID matching those provided in **Fig. 5**, (2) Unique ID generated for each locus being queried, (3) the chromosomal coordinates of each locus, (4) the number of sequences recovered in an oligonucleotide-duplex capture assay to determine candidate off-target loci, (5)

the number of MiSeq sequence reads obtained for ZFN-treated T-cells, (6) the corresponding indel percentage, (7) the number of MiSeq sequence reads obtained for GFP-treated T-cells, (8) the corresponding indel percentage for the GFP-treated T-cells, and (9) the Bonferroni-corrected p-value for statistical significance between indels in the TRAC-treated and GFP-treated samples. The indel percentage shown is background corrected where sequences common to both the TRAC ZFN-treated and GFP-treated samples are discarded from analysis. Panels (**a**)-(**e**) show the data obtained for TRAC 1-5, respectively. Each indel data point represents a single measurement.

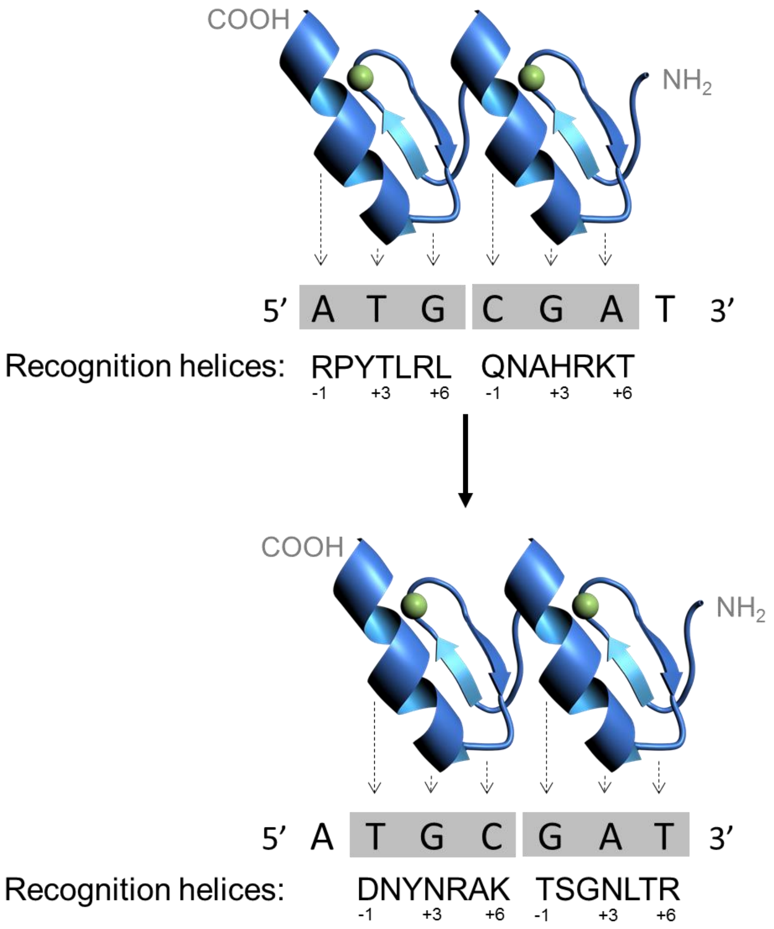

Supplementary Figure 24

Effect of shifting a two-finger zinc finger module binding site by a single base pair.

Shown at the top is an example of a two-finger zinc finger module binding to its target hexamer. Each individual finger binds to a target triplet which is highlighted with a grey box beneath each finger. Below each target triplet is the amino acid sequence of the recognition helix (amino to carboxy-terminal) of the finger binding to that triplet. Also denoted are the typical positions in the helix that make direct contacts with the DNA. The -1 position typically contacts the 3′ base of the triplet, the +3 position typically contacts the middle base of the triplet, and the +6 position typically contacts the 5′ base of the triplet (note the amino-terminus of the zinc finger module is at the 3′ end of the DNA). At the bottom is shown the effect of shifting the module binding site by a single base pair. Even though five of six base pairs are retained in the target hexamer, the stereochemistry of zinc finger-DNA recognition described above leads to new target triplets requiring new finger designs that are typically greatly diverged from the module at the top. This demonstrates how base-skipping linkers can lead to a large increase in the diversity of fingers that may be used to specify a given DNA segment (**Fig. 1d**).

Supplementary Table 1 | Single-stranded oligos used to generate engineered K562 cells with NC target sites. At the top are the oligos that were used for targeted insertion of CCR5 target sites into the AAVS1 locus. The oligo sequence is 5’ to 3’ and the binding sites for the CCR5 ZFNs are underlined. At the bottom are the corresponding oligos used for targeted insertion of AAVS1 target sites into the CCR5 locus. The cell lines generated were used in the first and second stages of screening ZFNs harboring the selected linkers from the bacterial selection system (**Supplementary Figure 6**).

Target Locus	Inserted Target Site	Oligo Name	Oligo Sequence
AAVS1	CCR5 CC target 5bp spacing	AAVS1-WTCCR5	CTGGGTACTTTTATCTGTCCCCTCCACCCCACAGTGGGGCtgGTCATCCTCATCctgatAAACTGCAAAAGgcCACTAGGGACAGGATTGGTGACAGAAAAGCCCCATCCTTA
AAVS1	CCR5 NC target 6bp spacing	AAVS1-CCR5HT6	CTGGGTACTTTTATCTGTCCCCTCCACCCCACAGTGGGGCatAAACTGCAAAAGgctctaGATGAGGATGACcaCACTAGGGACAGGATTGGTGACAGAAAAGCCCCATCCTTA
AAVS1	CCR5 NC target 7bp spacing	AAVS1-CCR5HT7	CTGGGTACTTTTATCTGTCCCCTCCACCCCACAGTGGGGCatAAACTGCAAAAGgcatctaGATGAGGATGACcaCACTAGGGACAGGATTGGTGACAGAAAAGCCCCATCCTTA

Target Locus	Inserted Target Site	Oligo Name	Oligo Sequence
CCR5	AAVS1 CC target 6bp spacing	CCR5-WTAAVS1	TCTTTGGTTTTGTGGGCAACATGCTGGTCATCCTCATCCTtcCCCTCCACCCCACAGTGGggatccTAGGGACAGGATTGGTGACagGATAAACTGCAAAAGGCTGAAGAGCATGACTGACATCTAC
CCR5	AAVS1 NC target 6bp spacing	CCR5-AAVSHT6	TCTTTGGTTTTGTGGGCAACATGCTGGTCATCCTCATCCTtcCCACTGTGGGGTGGAGGGggatccTAGGGACAGGATTGGTGACagGATAAACTGCAAAAGGCTGAAGAGCATGACTGACATCTAC
CCR5	AAVS1 NC target 7bp spacing	CCR5-AAVSHT7	TCTTTGGTTTTGTGGGCAACATGCTGGTCATCCTCATCCTtcCCACTGTGGGGTGGAGGGggatctcTAGGGACAGGATTGGTGACagGATAAACTGCAAAAGGCTGAAGAGCATGACTGACATCTAC

Supplementary Table 2 | Amino acid sequences of the key ZFNs used for these studies. Shown are the target gene, SBSID (unique identifier for each ZFN), the location of the FokI attachment point to the ZFP, and the full amino acid sequence of the indicated protein. For CCR5-R, the amino-terminal FokI harboring the randomized library is also shown.

Target Gene	SBSID	FokI Attachment Point	Amino Acid Sequence
CCR5	20505 (CCR5-R)	C-terminus	MDYKDHDGDYKDHDIDYKDDDDKMAPKKKRKVGIHGVPAAMAERPFQCRICMRNFSRS<br>DNLSVHIRTHTGEKPFACDICGRKFAQKINLQVHTKIHTGEKPFQCRICMRNFSRSDVLSEHI<br>RTHTGEKPFACDICGRKFAQRNHRTTHTKIHLRGSQLVKSELEEKKSELRHKLKYVPHEYIELIE<br>IARNSTQDRILEMKVMEFFMKVYGYRGKHLGGSRKPDGAIYTVGSPIDYGVIVDTKAYSGG<br>YNLPIGQADEMQRYVEENQTRNKHINPNEWWKVYPSSVTEFKFLFVSGHFKGNYKAQLT<br>RLNHITNCNGAVLSVEELLIGGEMIKAGTLTLEEVRRKFNNGEINFRS**
CCR5	8266 (CCR5-L)	C-terminus	MDYKDHDGDYKDHDIDYKDDDDKMAPKKKRKVGIHGVPAAMAERPFQCRICMRNFSDR<br>SNLSRHIRTHTGEKPFACDICGRKFAISSNLNSHTKIHTGSQKPFQCRICMRNFSRSDNLARH<br>IRTHTGEKPFACDICGRKFATSGNLTRHTKIHLRGSQLVKSELEEKKSELRHKLKYVPHEYIELIE<br>IARNSTQDRILEMKVMEFFMKVYGYRGKHLGGSRKPDGAIYTVGSPIDYGVIVDTKAYSGG<br>YNLPIGQADEMQRYVEENQTRNKHINPNEWWKVYPSSVTEFKFLFVSGHFKGNYKAQLT<br>RLNHITNCNGAVLSVEELLIGGEMIKAGTLTLEEVRRKFNNGEINFRS**
CCR5	20505 (CCR5-R) with linker library (used for selection)	N-terminus	MGQLVKSELEEKKSELRHKLKYVPHEYIELIEIARNSTQDRILEMKVMEFFMKVYGYRGKHLG<br>GSRKPDGAIYTVGSPIDYGVIVDTKAYSGGYNLPIGQADEMQRYVEENQTRNKHINPNEW<br>WKVYPSSVTEFKFLFVSGHFKGNYKAQLTRLNHITNCNGAVLSVEELLIGGEMIKAGTLTLEE<br>VRRKFNNGEINF – $(NNS)_{4\text{-}22}$<br>–RPFQCRICMRNFSRSDNLSVHIRTHTGEKPFACDICGRKFAQKINLQVHTKIHTGEKPFQC<br>RICMRNFSRSDVLSEHIRTHTGEKPFACDICGRKFAQRNHRTTHTKIHLRQKD**
CCR5	8266 (CCR5-L) (used for selection)	C-terminus	MAERPFQCRICMRNFSDRSNLSRHIRTHTGEKPFACDICGRKFAISSNLNSHTKIHTGSQKP<br>FQCRICMRNFSRSDNLARHIRTHTGEKPFACDICGRKFATSGNLTRHTKIHLRGSQLVKSELE<br>EKKSELRHKLKYVPHEYIELIEIARNSTQDRILEMKVMEFFMKVYGYRGKHLGGSRKPDGAIY<br>TVGSPIDYGVIVDTKAYSGGYNLPIGQADEMQRYVEENQTRNKHINPNEWWKVYPSSVTE<br>FKFLFVSGHFKGNYKAQLTRLNHITNCNGAVLSVEELLIGGEMIKAGTLTLEEVRRKFNNGEI<br>NF**

Target Gene	SBSID	FokI Attachment Point	Amino Acid Sequence
AAVS1	47694 (AAVS1-L)	C-terminus	MDYKDHDGDYKDHDIDYKDDDDKMAPKKKRKVGIHGVPAAMAERPFQCRICMRNFSRS<br>DHLSRHIRTHTGEKPFACDICGRKFATSGHLSRHTKIHTGERGFQCRICMRNFSYNWHLQRH<br>IRTHTGEKPFACDICGRKFARSDHLTTHTKIHTGSQKPFQCRICMRNFSHNYARDCHIRTHT<br>GEKPFACDICGRKFAQNSTRIGHTKIHLRGSQLVKSELEEKKSELRHKLKYVPHEYIELIEIARN<br>STQDRILEMKVMEFFMKVYGYRGKHLGGSRKPDGAIYTVGSPIDYGVIVDTKAYSGGYNLPI<br>GQADEMQRYVKENQTRNKHINPNEWWKVYPSSVTEFKFLFVSGHFKGNYKAQLTRLNRK<br>TNCNGAVLSVEELLIGGEMIKAGTLTLEEVRRKFNNGEINFRS**
AAVS1	47670 (AAVS1-R)	C-terminus	MDYKDHDGDYKDHDIDYKDDDDKMAPKKKRKVGIHGVPAAMAERPFQCRICMRKFARH<br>SHLTSHTKIHTGEKPFQCRICMRNFSTSGNLTRHIRTHTGEKPFACDICGRKFARRDWRRDH<br>TKIHTGSQKPFQCRICMRNFSQSSHLTRHIRTHTGEKPFACDICGRKFARLDNRTAHTKIHLR<br>GSQLVKSELEEKKSELRHKLKYVPHEYIELIEIARNSTQDRILEMKVMEFFMKVYGYRGKHLG<br>GSRKPDGAIYTVGSPIDYGVIVDTKAYSGGYNLPIGQADEMQRYVKENQTRNKHINPNEW<br>WKVYPSSVTEFKFLFVSGHFKGNYKAQLTRLNRKTNCNGAVLSVEELLIGGEMIKAGTLTLE<br>EVRRKFNNGEINF**

Target Gene	SBSID	FokI Attachment Point	Amino Acid Sequence
CEP290	61033	N-terminus	MDYKDHDGDYKDHDIDYKDDDDKMAPKKKRKVGIHGVPAAMGQLVKSELEEKKSELRHK<br>LKYVPHEYIELIEIARNSTQDRILEMKVMEFFMKVYGYRGKHLGGSRKPDGAIYTVGSPIDYG<br>VIVDTKAYSGGYNLPIGQADEMQRYVKENQTRNKHINPNEWWKVYPSSVTEFKFLFVSGH<br>FKGNYKAQLTRLNRKTNCNGAVLSVEELLIGGEMIKAGTLTLEEVRRKFNNGEINFSGTPHE<br>VGVYTLRPFQCRICMRNFSRSDNLARHIRTHTGEKPFACDICGRKFATSSNRKTHTKIHTGS<br>QKPFQCRICMRNFSRSDNLSEHIRTHTGEKPFACDICGRKFATSANLSRHTKIHTGSQKPFQ<br>CRICMRNFSRSDHLSQHIRTHTGEKPFACEICGRKFAASSNRITHTKIHLRQKD**
CEP290	61029	N-terminus	MDYKDHDGDYKDHDIDYKDDDDKMAPKKKRKVGIHGVPAAMGQLVKSELEEKKSELRHK<br>LKYVPHEYIELIEIARNSTQDRILEMKVMEFFMKVYGYRGKHLGGSRKPDGAIYTVGSPIDYG<br>VIVDTKAYSGGYNLPIGQADEMERYVEENQTRDKHLNPNEWWKVYPSSVTEFKFLFVSGH<br>FKGNYKAQLTRLNHITNCNGAVLSVEELLIGGEMIKAGTLTLEEVRRKFNNGEINFSGTPHEV<br>GVYTLRPFQCRICMRNFSHSNARKTHIRTHTGEKPFACDICGRKFAQSGSLTRHTKIHTGSQ<br>KPFQCRICMRNFSTSGSLSRHIRTHTGEKPFACDICGRKFAQKGTLMSHTKIHTGEKPFQCRI<br>CMRKFADRSTRTKHTKIHLRQKDRS**

Target Gene	SBSID	FokI Attachment Point	Amino Acid Sequence
TRAC - Site 1	55204	C-terminus	MDYKDHDGDYKDHDIDYKDDDDKMAPKKKRKVGIHGVPAAMAERPFQCRICMRNFSDR<br>SNLSRHIRTHTGEKPFACDICGRKFAQKVTLAAHTKIHTHPRAPIPKPFQCRICMRNFSDRS<br>ALSRHIRTHTGEKPFACDICGRKFATSGNLTRHTKIHTGSQKPFQCRICMRNFSYRSSLKEHIR<br>THTGEKPFACDICGRKFATSGNLTRHTKIHLRGSQLVKSELEEKKSELRHKLKYVPHEYIELIEIA<br>RNSTQDRILEMKVMEFFMKVYGYRGKHLGGSRKPDGAIYTVGSPIDYGVIVDTKAYSGGYN<br>LPIGQADEMERYVEENQTRDKHLNPNEWWKVYPSSVTEFKFLFVSGHFKGNYKAQLTRLN<br>HITNCNGAVLSVEELLIGGEMIKAGTLTLEEVRRKFNNGEINFRS**
TRAC - Site 1	53759	C-terminus	MDYKDHDGDYKDHDIDYKDDDDKMAPKKKRKVGIHGVPAAMAERPFQCRICMRNFSQQ<br>NVLINHIRTHTGEKPFACDICGRKFAQNATRTKHTKIHTGSQKPFQCRICMRNFSQSGHLA<br>RHIRTHTGEKPFACDICGRKFANRYDLMTHTKIHTHPRAPIPKPFQCRICMRNFSRSDSLLRH<br>IRTHTGEKPFACDICGRKFAQSSDLTRHTKIHLRGSQLVKSELEEKKSELRHKLKYVPHEYIELIE<br>IARNSTQDRILEMKVMEFFMKVYGYRGKHLGGSRKPDGAIYTVGSPIDYGVIVDTKAYSGG<br>YNLPIGQADEMQRYVKENQTRNKHINPNEWWKVYPSSVTEFKFLFVSGHFKGNYKAQLT<br>RLNRKTNCNGAVLSVEELLIGGEMIKAGTLTLEEVRRKFNNGEINF**
TRAC - Site 2	55229	C-terminus	MDYKDHDGDYKDHDIDYKDDDDKMAPKKKRKVGIHGVPAAMAERPFQCRICMRKFADR<br>SALARHTKIHTGEKPFQCRICMRNFSQSGNLARHIRTHTGEKPFACDICGRKFAHRSTLQGH<br>TKIHTGSQKPFQCRICMRNFSQSGDLTRHIRTHTGEKPFACDICGRKFATSGSLTRHTKIHLR<br>GSQLVKSELEEKKSELRHKLKYVPHEYIELIEIARNSTQDRILEMKVMEFFMKVYGYRGKHLG<br>GSRKPDGAIYTVGSPIDYGVIVDTKAYSGGYNLPIGQADEMERYVEENQTRDKHLNPNEW<br>WKVYPSSVTEFKFLFVSGHFKGNYKAQLTRLNHITNCNGAVLSVEELLIGGEMIKAGTLTLEE<br>VRRKFNNGEINFRS**
TRAC - Site 2	53785	C-terminus	MDYKDHDGDYKDHDIDYKDDDDKMAPKKKRKVGIHGVPAAMAERPFQCRICMRNFSQH<br>QVLVRHIRTHTGEKPFACDICGRKFAQNATRTKHTKIHTGEKPFQCRICMRKFAQSGHLSR<br>HTKIHTHPRAPIPKPFQCRICMRNFSDRSDLSRHIRTHTGEKPFACDICGRKFARSDALARHT<br>KIHLRGSQLVKSELEEKKSELRHKLKYVPHEYIELIEIARNSTQDRILEMKVMEFFMKVYGYRG<br>KHLGGSRKPDGAIYTVGSPIDYGVIVDTKAYSGGYNLPIGQADEMQRYVKENQTRNKHINP<br>NEWWKVYPSSVTEFKFLFVSGHFKGNYKAQLTRLNRKTNCNGAVLSVEELLIGGEMIKAGT<br>LTLEEVRRKFNNGEINF**

Target Gene	SBSID	FokI Attachment Point	Amino Acid Sequence
TRAC - Site 3	55266	N-terminus	MDYKDHDGDYKDHDIDYKDDDDKMAPKKKRKVGIHGVPAAMGQLVKSELEEKKSELRHKLKYVPHEYIELIEIARNSTQDRILEMKVMEFFMKVYGYRGKHLGGSRKPDGAIYTVGSPIDYGVIVDTKAYSGGYNLPIGQADEMQRYVKENQTRNKHINPNEWWKVYPSSVTEFKFLFVSGHFKGNYKAQLTRLNRKTNCNGAVLSVEELLIGGEMIKAGTLTLEEVRRKFNNGEINFSGAIRCHDEFWFRPFQCRICMRNFSQSSDLSRHIRTHTGEKPFACDICGRKFAQSGNRTTHTKIHTHPRAPIPKPFQCRICMRNFSRSANLARHIRTHTGEKPFACDICGRKFADRSALARHTKIHTGSQKPFQCRICMRNFSRSDVLSEHIRTHTGEKPFACDICGRKFAKHSTRRVHTKIHLRQKD**
TRAC - Site 3	53853	C-terminus	MDYKDHDGDYKDHDIDYKDDDDKMAPKKKRKVGIHGVPAAMAERPFQCRICMRNFSTMHQRVEHIRTHTGEKPFACDICGRKFATSGHLSRHTKIHTGSQKPFQCRICMRNFSRSDHLTQHIRTHTGEKPFACDICGRKFADSANLSRHTKIHTHPRAPIPKPFQCRICMRNFSQSGSLTRHIRTHTGEKPFACDICGRKFAAKWNLDAHTKIHLRGSQLVKSELEEKKSELRHKLKYVPHEYIELIEIARNSTQDRILEMKVMEFFMKVYGYRGKHLGGSRKPDGAIYTVGSPIDYGVIVDTKAYSGGYNLPIGQADEMERYVEENQTRDKHLNPNEWWKVYPSSVTEFKFLFVSGHFKGNYKAQLTRLNHITNCNGAVLSVEELLIGGEMIKAGTLTLEEVRRKFNNGEINFRS**
TRAC - Site 4	53885	N-terminus	MDYKDHDGDYKDHDIDYKDDDDKMAPKKKRKVGIHGVPAAMGQLVKSELEEKKSELRHKLKYVPHEYIELIEIARNSTQDRILEMKVMEFFMKVYGYRGKHLGGSRKPDGAIYTVGSPIDYGVIVDTKAYSGGYNLPIGQADEMQRYVKENQTRNKHINPNEWWKVYPSSVTEFKFLFVSGHFKGNYKAQLTRLNRKTNCNGAVLSVEELLIGGEMIKAGTLTLEEVRRKFNNGEINFSGTPHEVGVYTLRPFQCRICMRNFSRSDTLSEHIRTHTGEKPFACDICGRKFATSGSLTRHTKIHTGSQKPFQCRICMRNFSRSDHLSTHIRTHTGEKPFACDICGRKFATSSNRTKHTKIHTHPRAPIPKPFQCRICMRNFSRSDNLSEHIRTHTGEKPFACDICGRKFAWHSSLRVHTKIHLRQKD**
TRAC - Site 4	53909	C-terminus	MDYKDHDGDYKDHDIDYKDDDDKMAPKKKRKVGIHGVPAAMAERPFQCRICMRNFSRSAHLSRHIRTHTGEKPFACDICGRKFADRSDLSRHTKIHTGSQKPFQCRICMRNFSRSDVLSVHIRTHTGEKPFACDICGRKFAQNNHRITHTKIHTGSQKPFQCRICMRNFSRSDVLSEHIRTHTGEKPFACDICGRKFASPSSRRTHTKIHLRGSQLVKSELEEKKSELRHKLKYVPHEYIELIEIARNSTQDRILEMKVMEFFMKVYGYRGKHLGGSRKPDGAIYTVGSPIDYGVIVDTKAYSGGYNLPIGQADEMERYVEENQTRDKHLNPNEWWKVYPSSVTEFKFLFVSGHFKGNYKAQLTRLNHITNCNGAVLSVEELLIGGEMIKAGTLTLEEVRRKFNNGEINFRS**
TRAC - Site 5	55248	C-terminus	MDYKDHDGDYKDHDIDYKDDDDKMAPKKKRKVGIHGVPAAMAERPFQCRICMRNFSDQSNLRAHIRTHTGEKPFACDICGRKFATSSNRKTHTKIHTGSQKPFQCRICMRNFSLQQTLADHIRTHTGEKPFACDICGRKFAQSGNLARHTKIHTGSQKPFQCRICMRNFSRREDLITHIRTHTGEKPFACDICGRKFATSSNLSRHTKIHLRGSQLVKSELEEKKSELRHKLKYVPHEYIELIEIARNSTQDRILEMKVMEFFMKVYGYRGKHLGGSRKPDGAIYTVGSPIDYGVIVDTKAYSGGYNLPIGQADEMQRYVKENQTRNKHINPNEWWKVYPSSVTEFKFLFVSGHFKGNYKAQLTRLNRKTNCNGAVLSVEELLIGGEMIKAGTLTLEEVRRKFNNGEINF**
TRAC - Site 5	55254	C-terminus	MDYKDHDGDYKDHDIDYKDDDDKMAPKKKRKVGIHGVPAAMAERPFQCRICMRNFSRSDHLSTHIRTHTGEKPFACDICGRKFADRSHLARHTKIHTGSQKPFQCRICMRKFALKQHLNEHTKIHTGEKPFQCRICMRNFSQSGNLARHIRTHTGEKPFACDICGRKFAHNSSLKDHTKIHLRGSQLVKSELEEKKSELRHKLKYVPHEYIELIEIARNSTQDRILEMKVMEFFMKVYGYRGKHLGGSRKPDGAIYTVGSPIDYGVIVDTKAYSGGYNLPIGQADEMERYVEENQTRDKHLNPNEWWKVYPSSVTEFKFLFVSGHFKGNYKAQLTRLNHITNCNGAVLSVEELLIGGEMIKAGTLTLEEVRRKFNNGEINFRS**

Supplementary Table 3 | DNA sequences of the key ZFNs used for these studies. Shown are the target gene, SBSID (unique identifier for each ZFN), the location of the FokI attachment point to the ZFP, and the full DNA sequence encoding the indicated protein.

Target Gene	SBSID	FokI Attachment Point	DNA Sequence
CCR5	20505 (CCR5-R)	C-terminus	ATGGACTACAAAGACCATGACGGTGATTATAAAGATCATGACATCGATTACAAGGATGACG<br>ATGACAAGATGGCCCCCAAGAAGAAGAGGAAGGTCGGCATTCATGGGGTACCCGCCGCTAT<br>GGCCGAGAGGCCCTTCCAGTGTCGGATCTGCATGCGGAACTTCAGCAGGAGCGACAACCTGA<br>GCGTACACATCCGCACCCACACAGGCGAGAAGCCTTTTGCCTGTGACATTTGTGGGAGGAAA<br>TTTGCCCAGAAAATCAACCTCCAGGTCCACACCAAGATCCACACCGGAGAGAAGCCCTTTCA<br>GTGCAGAATCTGCATGAGAAACTTCTCCCGGTCCGACGTGCTGAGCGAGCACATTAGGACCC<br>ACACCGGGGAGAAACCCTTCGCCTGCGACATCTGTGGCCGCAAATTTGCCCAGCGCAACCAC<br>CGGACAACACACACAAAGATTCACCTGCGGGGATCCCAGCTGGTGAAGAGCGAGCTGGAGG<br>AGAAGAAGTCCGAGCTGCGGCACAAGCTGAAGTACGTGCCCCACGAGTACATCGAGCTGAT<br>CGAGATCGCCAGGAACAGCACCCAGGACCGCATCCTGGAGATGAAGGTGATGGAGTTCTTC<br>ATGAAGGTGTACGGCTACAGGGGAAAGCACCTGGGCGGAAGCAGAAAGCCTGACGGCGCC<br>ATCTATACAGTGGGCAGCCCCATCGATTACGGCGTGATCGTGGACACAAAGGCCTACAGCGG<br>CGGCTACAATCTGCCTATCGGCCAGGCCGACGAGATGCAGAGATACGTGAAGGAGAACCAG<br>ACCCGGAATAAGCACATCAACCCCAACGAGTGGTGGAAGGTGTACCCTAGCAGCGTGACCG<br>AGTTCAAGTTCCTGTTCGTGAGCGGCCACTTCAAGGGCAACTACAAGGCCCAGCTGACCAGG<br>CTGAACCGCAAAACCAACTGCAATGGCGCCGTGCTGAGCGTGGAGGAGCTGCTGATCGGCG<br>GCGAGATGATCAAAGCCGGCACCCTGACACTGGAGGAGGTGCGGCGCAAGTTCAACAACGG<br>CGAGATCAACTTCTGATAA
CCR5	8266 (CCR5-L)	C-terminus	ATGGACTACAAAGACCATGACGGTGATTATAAAGATCATGACATCGATTACAAGGATGACG<br>ATGACAAGATGGCCCCCAAGAAGAAGAGGAAGGTCGGCATCCACGGGGTACCCGCCGCCAT<br>GGCTGAGAGGCCCTTCCAGTGTCGAATCTGCATGCGTAACTTCAGTGACCGCTCCAACCTGTC<br>CCGCCACATCCGCACCCACACAGGCGAGAAGCCTTTTGCCTGTGACATTTGTGGGAGGAAGT<br>TTGCCATCTCCTCCAACCTGAACTCCCATACCAAGATACACACGGGATCTCAGAAGCCCTTCC<br>AGTGTCGAATCTGCATGCGTAACTTCAGTCGCTCCGACAACCTGGCCCGCCACATCCGCACCC<br>ACACAGGCGAGAAGCCTTTTGCCTGTGACATTTGTGGGAGGAAGTTTGCCACCTCCGGCAAC<br>CTGACCCGCCATACCAAGATACACCTGCGGGGATCCCAGCTGGTGAAGAGCGAGCTGGAGG<br>AGAAGAAGTCCGAGCTGCGGCACAAGCTGAAGTACGTGCCCCACGAGTACATCGAGCTGAT<br>CGAGATCGCCAGGAACAGCACCCAGGACCGCATCCTGGAGATGAAGGTGATGGAGTTCTTC<br>ATGAAGGTGTACGGCTACAGGGGAAAGCACCTGGGCGGAAGCAGAAAGCCTGACGGCGCC<br>ATCTATACAGTGGGCAGCCCCATCGATTACGGCGTGATCGTGGACACAAAGGCCTACAGCGG<br>CGGCTACAATCTGCCTATCGGCCAGGCCGACGAGATGCAGAGATACGTGGAGGAGAACCAG<br>ACCCGGAATAAGCACATCAACCCCAACGAGTGGTGGAAGGTGTACCCTAGCAGCGTGACCG<br>AGTTCAAGTTCCTGTTCGTGAGCGGCCACTTCAAGGGCAACTACAAGGCCCAGCTGACCAGG<br>CTGAACCACATCACCAACTGCAATGGCGCCGTGCTGAGCGTGGAGGAGCTGCTGATCGGCGG<br>CGAGATGATCAAAGCCGGCACCCTGACACTGGAGGAGGTGCGGCGCAAGTTCAACAACGGC<br>GAGATCAACTTCAGATCTTGATAA
CCR5	20505 (CCR5-R) with linker library (used for selection)	N-terminus	ATGGGACAGCTGGTGAAGAGCGAGCTGGAGGAGAAGAAGTCCGAGCTGCGGCACAAGCTG<br>AAGTACGTGCCCCACGAGTACATCGAGCTGATCGAGATCGCCAGGAACAGCACCCAGGACC<br>GCATCCTGGAGATGAAGGTGATGGAGTTCTTCATGAAGGTGTACGGCTACAGGGGAAAGCA<br>CCTGGGCGGAAGCAGAAAGCCTGACGGCGCCATCTATACAGTGGGCAGCCCCATCGATTAC<br>GGCGTGATCGTGGACACAAAGGCCTACAGCGGCGGCTACAATCTGCCTATCGGCCAGGCCG<br>ACGAGATGGAGAGATACGTGGAGGAGAACCAGACCCGGGATAAGCACCTCAACCCCAACG<br>AGTGGTGGAAGGTGTACCCTAGCAGCGTGACCGAGTTCAAGTTCCTGTTCGTGAGCGGCCAC<br>TTCAAGGGCAACTACAAGGCCCAGCTGACCAGGCTGAACCACATCACCAACTGCAATGGCG<br>CCGTGCTGAGCGTGGAGGAGCTGCTGATCGGCGGCGAGATGATCAAAGCCGGCACCCTGAC<br>ACTGGAGGAGGTGCGGCGCAAGTTCAACAACGGCGAGATCAACTTC - $(NNS)_{4\text{-}22}$ -<br>AGGCCCTTCCAGTGTCGGATCTGCATGCGGAACTTCAGCAGGAGCGACAACCTGAGCGTACA<br>CATCCGCACCCACACAGGCGAGAAGCCTTTTGCCTGTGACATTTGTGGGAGGAAATTTGCCC<br>AGAAAATCAACCTCCAGGTCCACACCAAGATCCACACCGGAGAGAAGCCCTTTCAGTGCAG<br>AATCTGCATGAGAAACTTCTCCCGGTCCGACGTGCTGAGCGAGCACATTAGGACCCACACCG<br>GGGAGAAACCCTTCGCCTGCGACATCTGTGGCCGCAAATTTGCCCAGCGCAACCACCGGACA<br>ACACACACAAAGATTCACCTGCGGCAGAAGGACTGATAA

Target Gene	SBSID	FokI Attachment Point	DNA Sequence
CCR5	8266 (CCR5-L) (used for selection)	C-terminus	ATGGCTGAGAGGCCCTTCCAGTGTCGAATCTGCATGCGTAACTTCAGTGACCGCTCCAACCTG<br>TCCCGCCACATCCGCACCCACACAGGCGAGAAGCCTTTTGCCTGTGACATTTGTGGGAGGAA<br>GTTTGCCATCTCCTCCAACCTGAACTCCCATACCAAGATACACACGGGATCTCAGAAGCCCTT<br>CCAGTGTCGAATCTGCATGCGTAACTTCAGTCGCTCCGACAACCTGGCCCGCCACATCCGCAC<br>CCACACAGGCGAGAAGCCTTTTGCCTGTGACATTTGTGGGAGGAAGTTTGCCACCTCCGGCA<br>ACCTGACCCGCCATACCAAGATACACCTGCGGGGATCCCAGCTGGTGAAGAGCGAGCTGGA<br>GGAGAAGAAGTCCGAGCTGCGGCACAAGCTGAAGTACGTGCCCCACGAGTACATCGAGCTG<br>ATCGAGATCGCCAGGAACAGCACCCAGGACCGCATCCTGGAGATGAAGGTGATGGAGTTCT<br>TCATGAAGGTGTACGGCTACAGGGGAAAGCACCTGGGCGGAAGCAGAAAGCCTGACGGCG<br>CCATCTATACAGTGGGCAGCCCCATCGATTACGGCGTGATCGTGGACACAAAGGCCTACAGC<br>GGCGGCTACAATCTGCCTATCGGCCAGGCCGACGAGATGCAGAGATACGTGGAGGAGAACC<br>AGACCCGGAATAAGCACATCAACCCCAACGAGTGGTGGAAGGTGTACCCTAGCAGCGTGAC<br>CGAGTTCAAGTTCCTGTTCGTGAGCGGCCACTTCAAGGGCAACTACAAGGCCCAGCTGACCA<br>GGCTGAACCACATCACCAACTGCAATGGCGCCGTGCTGAGCGTGGAGGAGCTGCTGATCGGC<br>GGCGAGATGATCAAAGCCGGCACCCTGACACTGGAGGAGGTGCGGCGCAAGTTCAACAACG<br>GCGAGATCAACTTCAGATCTTGATAA
AAVS1	47694 (AAVS1-L)	C-terminus	ATGGACTACAAAGACCATGACGGTGATTATAAAGATCATGACATCGATTACAAGGATGACG<br>ATGACAAGATGGCCCCCAAGAAGAAGAGGAAGGTCGGCATCCACGGGGTACCCGCCGCTAT<br>GGCTGAGAGGCCCTTCCAGTGTCGAATCTGCATGCGTAACTTCAGTCGCTCCGACCACCTGTC<br>CCGCCACATCCGCACCCACACCGGCGAGAAGCCTTTTGCCTGTGACATTTGTGGGAGGAAAT<br>TTGCCACCTCCGGCCACCTGTCCCGCCATACCAAGATACACACAGGCGAAAGGGGATTCCAG<br>TGTCGAATCTGCATGCGTAACTTCAGTTACAACTGGCACCTGCAGCGCCACATCCGCACCCAC<br>ACCGGCGAGAAGCCTTTTGCCTGTGACATTTGTGGGAGGAAATTTGCCCGCTCCGACCACCTG<br>ACCACCCATACCAAGATACACACGGGATCTCAGAAGCCCTTCCAGTGTCGAATCTGCATGCG<br>TAACTTCAGTCACAACTACGCCCGCGACTGTCACATCCGCACCCACACCGGCGAGAAGCCTTT<br>TGCCTGTGACATTTGTGGGAGGAAATTTGCCCAGAACTCCACCCGCATCGGCCATACCAAGA<br>TACACCTGCGGGGATCCCAGCTGGTGAAGAGCGAGCTGGAGGAGAAGAAGTCCGAGCTGCG<br>GCACAAGCTGAAGTACGTGCCCCACGAGTACATCGAGCTGATCGAGATCGCCAGGAACAGC<br>ACCCAGGACCGCATCCTGGAGATGAAGGTGATGGAGTTCTTCATGAAGGTGTACGGCTACAG<br>GGGAAAGCACCTGGGCGGAAGCAGAAAGCCTGACGGCGCCATCTATACAGTGGGCAGCCCC<br>ATCGATTACGGCGTGATCGTGGACACAAAGGCCTACAGCGGCGGCTACAATCTGCCTATCGG<br>CCAGGCCGACGAGATGGAGAGATACGTGGAGGAGAACCAGACCCGGGATAAGCACCTCAA<br>CCCCAACGAGTGGTGGAAGGTGTACCCTAGCAGCGTGACCGAGTTCAAGTTCCTGTTCGTGA<br>GCGGCCACTTCAAGGGCAACTACAAGGCCCAGCTGACCAGGCTGAACCACATCACCAACTG<br>CAATGGCGCCGTGCTGAGCGTGGAGGAGCTGCTGATCGGCGGCGAGATGATCAAAGCCGGC<br>ACCCTGACACTGGAGGAGGTGCGGCGCAAGTTCAACAACGGCGAGATCAACTTCAGATCTTG<br>ATAA
AAVS1	47670 (AAVS1-R)	C-terminus	ATGGACTACAAAGACCATGACGGTGATTATAAAGATCATGACATCGATTACAAGGATGACG<br>ATGACAAGATGGCCCCCAAGAAGAAGAGGAAGGTCGGCATTCATGGGGTACCCGCCGCTAT<br>GGCTGAGAGGCCCTTCCAGTGTCGAATCTGCATGCGTAAGTTTGCCCGCCACTCCCACCTGAC<br>CTCCCATACCAAGATACACACGGGCGAGAAGCCCTTCCAGTGTCGAATCTGCATGCGTAACT<br>TCAGTACCTCCGGCAACCTGACCCGCCACATCCGCACCCACACCGGCGAGAAGCCTTTTGCCT<br>GTGACATTTGTGGGAGGAAATTTGCCCGCCGCGACTGGCGCCGCGACCATACCAAGATACAC<br>ACGGGATCTCAGAAGCCCTTCCAGTGTCGAATCTGCATGCGTAACTTCAGTCAGTCCTCCCAC<br>CTGACCCGCCACATCCGCACCCACACCGGCGAGAAGCCTTTTGCCTGTGACATTTGTGGGAG<br>GAAATTTGCCCGCCTGGACAACCGCACCGCCCATACCAAGATACACCTGCGGGGATCCCAGC<br>TGGTGAAGAGCGAGCTGGAGGAGAAGAAGTCCGAGCTGCGGCACAAGCTGAAGTACGTGC<br>CCCACGAGTACATCGAGCTGATCGAGATCGCCAGGAACAGCACCCAGGACCGCATCCTGGA<br>GATGAAGGTGATGGAGTTCTTCATGAAGGTGTACGGCTACAGGGGAAAGCACCTGGGCGGA<br>AGCAGAAAGCCTGACGGCGCCATCTATACAGTGGGCAGCCCCATCGATTACGGCGTGATCGT<br>GGACACAAAGGCCTACAGCGGCGGCTACAATCTGCCTATCGGCCAGGCCGACGAGATGCAG<br>AGATACGTGAAGGAGAACCAGACCCGGAATAAGCACATCAACCCCAACGAGTGGTGGAAG<br>GTGTACCCTAGCAGCGTGACCGAGTTCAAGTTCCTGTTCGTGAGCGGCCACTTCAAGGGCAA<br>CTACAAGGCCCAGCTGACCAGGCTGAACCGCAAAACCAACTGCAATGGCGCCGTGCTGAGC<br>GTGGAGGAGCTGCTGATCGGCGGCGAGATGATCAAAGCCGGCACCCTGACACTGGAGGAGG<br>TGCGGCGCAAGTTCAACAACGGCGAGATCAACTTCTGATAA

Target Gene	SBSID	FokI Attachment Point	DNA Sequence
CEP290	61033	N-terminus	ATGGACTACAAAGACCATGACGGTGATTATAAAGATCATGACATCGATTACAAGGATGACGATGACAAGATGGCCCCCAAGAAGAAGAGGAAGGTCGGCATTCATGGGGTACCCGCCGCTATGGGACAGCTGGTGAAGAGCGAGCTGGAGGAGAAGAAGTCCGAGCTGCGGCACAAGCTGAAGTACGTGCCCCACGAGTACATCGAGCTGATCGAGATCGCCAGGAACAGCACCCAGGACCGCATCCTGGAGATGAAGGTGATGGAGTTCTTCATGAAGGTGTACGGCTACAGGGGAAAGCACCTGGGCGGAAGCAGAAAGCCTGACGGCGCCATCTATACAGTGGGCAGCCCCATCGATTACGGCGTGATCGTGGACACAAAGGCCTACAGCGGCGGCTACAATCTGCCTATCGGCCAGGCCGACGAGATGCAGAGATACGTGAAGGAGAACCAGACCCGGAATAAGCACATCAACCCCAACGAGTGGTGGAAGGTGTACCCTAGCAGCGTGACCGAGTTCAAGTTCCTGTTCGTGAGCGGCCACTTCAAGGGCAACTACAAGGCCCAGCTGACCAGGCTGAACCGCAAAACCAACTGCAATGGCGCCGTGCTGAGCGTGGAGGAGCTGCTGATCGGCGGCGAGATGATCAAAGCCGGCACCCTGACACTGGAGGAGGTGCGGCGCAAGTTCAACAACGGCGAGATCAACTTCAGCGGCACTCCACACGAAGTGGGAGTGTACACACTTAGGCCCTTCCAGTGTCGAATCTGCATGCGTAACTTCAGTCGCTCCGACAACCTGGCCCGCCACATCCGCACCCACACCGGCGAGAAGCCTTTTGCCTGTGACATTTGTGGGAGGAAATTTGCCACCTCCTCCAACCGCAAGACCCATACCAAGATACACACGGGCAGCCAAAAGCCCTTCCAGTGTCGAATCTGCATGCGTAACTTCAGTCGCTCCGACAACCTGTCCGAGCACATCCGCACCCACACCGGCGAGAAGCCTTTTGCCTGTGACATTTGTGGGAGGAAATTTGCCACCTCCGCCAACCTGTCCCGCCATACCAAGATACACACGGGATCTCAGAAGCCCTTCCAGTGTCGAATCTGCATGCGTAACTTCAGTCGTAGTGACCACCTGAGCCAGCACATCCGCACCCACACAGGCGAGAAGCCTTTTGCCTGTGAGATTTGTGGGAGGAAATTTGCCGCCAGCAGCAACCGCATAACGCATACCAAGATACACCTGCGGCAGAAGGACTGATAA
CEP290	61029	N-terminus	ATGGACTACAAAGACCATGACGGTGATTATAAAGATCATGACATCGATTACAAGGATGACGATGACAAGATGGCCCCCAAGAAGAAGAGGAAGGTCGGCATCCACGGGGTACCCGCCGCTATGGGACAGCTGGTGAAGAGCGAGCTGGAGGAGAAGAAGTCCGAGCTGCGGCACAAGCTGAAGTACGTGCCCCACGAGTACATCGAGCTGATCGAGATCGCCAGGAACAGCACCCAGGACCGCATCCTGGAGATGAAGGTGATGGAGTTCTTCATGAAGGTGTACGGCTACAGGGGAAAGCACCTGGGCGGAAGCAGAAAGCCTGACGGCGCCATCTATACAGTGGGCAGCCCCATCGATTACGGCGTGATCGTGGACACAAAGGCCTACAGCGGCGGCTACAATCTGCCTATCGGCCAGGCCGACGAGATGGAGAGATACGTGGAGGAGAACCAGACCCGGGATAAGCACCTCAACCCCAACGAGTGGTGGAAGGTGTACCCTAGCAGCGTGACCGAGTTCAAGTTCCTGTTCGTGAGCGGCCACTTCAAGGGCAACTACAAGGCCCAGCTGACCAGGCTGAACCACATCACCAACTGCAATGGCGCCGTGCTGAGCGTGGAGGAGCTGCTGATCGGCGGCGAGATGATCAAAGCCGGCACCCTGACACTGGAGGAGGTGCGGCGCAAGTTCAACAACGGCGAGATCAACTTCAGCGGCACTCCACACGAAGTGGGAGTGTACACACTTAGGCCCTTCCAGTGTCGAATCTGCATGCGTAACTTCAGTCACTCCAACGCCCGCAAGACCCACATCCGCACCCACACCGGCGAGAAGCCTTTTGCCTGTGACATTTGTGGGAGGAAATTTGCCCAGTCCGGCTCCCTGACCCGCCATACCAAGATACACACGGGCAGCCAAAAGCCCTTCCAGTGTCGAATCTGCATGCGTAACTTCAGTACCTCCGGCTCCCTGTCCCGCCACATCCGCACCCACACCGGCGAGAAGCCTTTTGCCTGTGACATTTGTGGGAGGAAATTTGCCCAGAAGGGCACCCTGATGTCCCATACCAAGATACACACGGGCGAGAAGCCCTTCCAGTGTCGAATCTGCATGCGTAAGTTTGCCGACCGCTCCACCCGCACCAAGCATACCAAGATACACCTGCGGCAGAAGGACAGATCTTGATAA

Target Gene	SBSID	FokI Attachment Point	DNA Sequence
TRAC - Site 1	55204	C-terminus	ATGGACTACAAAGACCATGACGGTGATTATAAAGATCATGACATCGATTACAAGGATGACG<br>ATGACAAGATGGCCCCCAAGAAGAAGAGGAAGGTCGGCATCCACGGGGTACCCGCCGCTAT<br>GGCTGAGAGGCCCTTCCAGTGTCGAATCTGCATGCGTAACTTCAGTGACCGCTCCAACCTGTC<br>CCGCCACATCCGCACCCACACCGGCGAGAAGCCTTTTGCCTGTGACATTTGTGGGAGGAAAT<br>TTGCCCAGAAGGTGACCCTGGCCGCCCATACCAAGATACACACGCATCCCAGGGCACCTATT<br>CCCAAGCCCTTCCAGTGTCGAATCTGCATGCGTAACTTCAGTGACCGCTCCGCCCTGTCCCGC<br>CACATCCGCACCCACACCGGCGAGAAGCCTTTTGCCTGTGACATTTGTGGGAGGAAATTTGCC<br>ACCTCCGGCAACCTGACCCGCCATACCAAGATACACACGGGATCTCAGAAGCCCTTCCAGTG<br>TCGAATCTGCATGCGTAACTTCAGTTACCGCTCCTCCCTGAAGGAGCACATCCGCACCCACAC<br>CGGCGAGAAGCCTTTTGCCTGTGACATTTGTGGGAGGAAATTTGCCACCTCCGGCAACCTGAC<br>CCGCCATACCAAGATACACCTGCGGGGATCCCAGCTGGTGAAGAGCGAGCTGGAGGAGAAG<br>AAGTCCGAGCTGCGGCACAAGCTGAAGTACGTGCCCCACGAGTACATCGAGCTGATCGAGA<br>TCGCCAGGAACAGCACCCAGGACCGCATCCTGGAGATGAAGGTGATGGAGTTCTTCATGAA<br>GGTGTACGGCTACAGGGGAAAGCACCTGGGCGGAAGCAGAAAGCCTGACGGCGCCATCTAT<br>ACAGTGGGCAGCCCCATCGATTACGGCGTGATCGTGGACACAAAGGCCTACAGCGGCGGCT<br>ACAATCTGCCTATCGGCCAGGCCGACGAGATGGAGAGATACGTGGAGGAGAACCAGACCCG<br>GGATAAGCACCTCAACCCCAACGAGTGGTGGAAGGTGTACCCTAGCAGCGTGACCGAGTTC<br>AAGTTCCTGTTCGTGAGCGGCCACTTCAAGGGCAACTACAAGGCCCAGCTGACCAGGCTGAA<br>CCACATCACCAACTGCAATGGCGCCGTGCTGAGCGTGGAGGAGCTGCTGATCGGCGGCGAG<br>ATGATCAAAGCCGGCACCCTGACACTGGAGGAGGTGCGGCGCAAGTTCAACAACGGCGAGA<br>TCAACTTCAGATCTTGATAA
TRAC - Site 1	53759	C-terminus	ATGGACTACAAAGACCATGACGGTGATTATAAAGATCATGACATCGATTACAAGGATGACG<br>ATGACAAGATGGCCCCCAAGAAGAAGAGGAAGGTCGGCATTCATGGGGTACCCGCCGCTAT<br>GGCTGAGAGGCCCTTCCAGTGTCGAATCTGCATGCGTAACTTCAGTCAGCAGAACGTGCTGA<br>TCAACCACATCCGCACCCACACCGGCGAGAAGCCTTTTGCCTGTGACATTTGTGGGAGGAAA<br>TTTGCCCAGAACGCCACCCGCACCAAGCATACCAAGATACACACGGGCAGCCAAAAGCCCTT<br>CCAGTGTCGAATCTGCATGCGTAACTTCAGTCAGTCCGGCCACCTGGCCCGCCACATCCGCAC<br>CCACACCGGCGAGAAGCCTTTTGCCTGTGACATTTGTGGGAGGAAATTTGCCAACCGCTACG<br>ACCTGATGACCCATACCAAGATACACACGCACCCGCGCGCCCCGATCCCGAAGCCCTTCCAG<br>TGTCGAATCTGCATGCGTAACTTCAGTCGCTCCGACTCCCTGCTGCGCCACATCCGCACCCAC<br>ACCGGCGAGAAGCCTTTTGCCTGTGACATTTGTGGGAGGAAATTTGCCCAGTCCTCCGACCTG<br>ACCCGCCATACCAAGATACACCTGCGGGGATCCCAGCTGGTGAAGAGCGAGCTGGAGGAGA<br>AGAAGTCCGAGCTGCGGCACAAGCTGAAGTACGTGCCCCACGAGTACATCGAGCTGATCGA<br>GATCGCCAGGAACAGCACCCAGGACCGCATCCTGGAGATGAAGGTGATGGAGTTCTTCATG<br>AAGGTGTACGGCTACAGGGGAAAGCACCTGGGCGGAAGCAGAAAGCCTGACGGCGCCATCT<br>ATACAGTGGGCAGCCCCATCGATTACGGCGTGATCGTGGACACAAAGGCCTACAGCGGCGG<br>CTACAATCTGCCTATCGGCCAGGCCGACGAGATGCAGAGATACGTGAAGGAGAACCAGACC<br>CGGAATAAGCACATCAACCCCAACGAGTGGTGGAAGGTGTACCCTAGCAGCGTGACCGAGT<br>TCAAGTTCCTGTTCGTGAGCGGCCACTTCAAGGGCAACTACAAGGCCCAGCTGACCAGGCTG<br>AACCGCAAAACCAACTGCAATGGCGCCGTGCTGAGCGTGGAGGAGCTGCTGATCGGCGGCG<br>AGATGATCAAAGCCGGCACCCTGACACTGGAGGAGGTGCGGCGCAAGTTCAACAACGGCGA<br>GATCAACTTCTGATAA
TRAC - Site 2	55229	C-terminus	ATGGACTACAAAGACCATGACGGTGATTATAAAGATCATGACATCGATTACAAGGATGACG<br>ATGACAAGATGGCCCCCAAGAAGAAGAGGAAGGTCGGCATCCACGGGGTACCCGCCGCTAT<br>GGCTGAGAGGCCCTTCCAGTGTCGAATCTGCATGCGTAAGTTTGCCGACCGCTCCGCCCTGGC<br>CCGCCATACCAAGATACACACGGGCGAGAAGCCCTTCCAGTGTCGAATCTGCATGCGTAACT<br>TCAGTCAGTCCGGCAACCTGGCCCGCCACATCCGCACCCACACCGGCGAGAAGCCTTTTGCCT<br>GTGACATTTGTGGGAGGAAATTTGCCCACCGCTCCACCCTGCAGGGCCATACCAAGATACAC<br>ACGGGATCTCAGAAGCCCTTCCAGTGTCGAATCTGCATGCGTAACTTCAGTCAGTCCGGCGAC<br>CTGACCCGCCACATCCGCACCCACACCGGCGAGAAGCCTTTTGCCTGTGACATTTGTGGGAG<br>GAAATTTGCCACCTCCGGCTCCCTGACCCGCCATACCAAGATACACCTGCGGGGATCCCAGCT<br>GGTGAAGAGCGAGCTGGAGGAGAAGAAGTCCGAGCTGCGGCACAAGCTGAAGTACGTGCC<br>CCACGAGTACATCGAGCTGATCGAGATCGCCAGGAACAGCACCCAGGACCGCATCCTGGAG<br>ATGAAGGTGATGGAGTTCTTCATGAAGGTGTACGGCTACAGGGGAAAGCACCTGGGCGGAA<br>GCAGAAAGCCTGACGGCGCCATCTATACAGTGGGCAGCCCCATCGATTACGGCGTGATCGTG<br>GACACAAAGGCCTACAGCGGCGGCTACAATCTGCCTATCGGCCAGGCCGACGAGATGGAGA<br>GATACGTGGAGGAGAACCAGACCCGGGATAAGCACCTCAACCCCAACGAGTGGTGGAAGGT<br>GTACCCTAGCAGCGTGACCGAGTTCAAGTTCCTGTTCGTGAGCGGCCACTTCAAGGGCAACT<br>ACAAGGCCCAGCTGACCAGGCTGAACCACATCACCAACTGCAATGGCGCCGTGCTGAGCGT<br>GGAGGAGCTGCTGATCGGCGGCGAGATGATCAAAGCCGGCACCCTGACACTGGAGGAGGTG<br>CGGCGCAAGTTCAACAACGGCGAGATCAACTTCAGATCTTGATAA

Target Gene	SBSID	FokI Attachment Point	DNA Sequence
TRAC - Site 2	53785	C-terminus	ATGGACTACAAAGACCATGACGGTGATTATAAAGATCATGACATCGATTACAAGGATGACG<br>ATGACAAGATGGCCCCCAAGAAGAAGAGGAAGGTCGGCATTCATGGGGTACCCGCCGCTAT<br>GGCTGAGAGGCCCTTCCAGTGTCGAATCTGCATGCGTAACTTCAGTCAGCACCAGGTGCTGGT<br>GCGCCACATCCGCACCCACACCGGCGAGAAGCCTTTTGCCTGTGACATTTGTGGGAGGAAAT<br>TTGCCCAGAACGCCACCCGCACCAAGCATACCAAGATACACACGGGCGAGAAGCCCTTCCA<br>GTGTCGAATCTGCATGCGTAAGTTTGCCCAGTCCGGCCACCTGTCCCGCCATACCAAGATACA<br>CACGCACCCGCGCGCCCCGATCCCGAAGCCCTTCCAGTGTCGAATCTGCATGCGTAACTTCAG<br>TGACCGCTCCGACCTGTCCCGCCACATCCGCACCCACACCGGCGAGAAGCCTTTTGCCTGTGA<br>CATTTGTGGGAGGAAATTTGCCCGCTCCGACGCCCTGGCCCGCCATACCAAGATACACCTGC<br>GGGGATCCCAGCTGGTGAAGAGCGAGCTGGAGGAGAAGAAGTCCGAGCTGCGGCACAAGC<br>TGAAGTACGTGCCCCACGAGTACATCGAGCTGATCGAGATCGCCAGGAACAGCACCCAGGA<br>CCGCATCCTGGAGATGAAGGTGATGGAGTTCTTCATGAAGGTGTACGGCTACAGGGGAAAG<br>CACCTGGGCGGAAGCAGAAAGCCTGACGGCGCCATCTATACAGTGGGCAGCCCCATCGATT<br>ACGGCGTGATCGTGGACACAAAGGCCTACAGCGGCGGCTACAATCTGCCTATCGGCCAGGCC<br>GACGAGATGCAGAGATACGTGAAGGAGAACCAGACCCGGAATAAGCACATCAACCCCAAC<br>GAGTGGTGGAAGGTGTACCCTAGCAGCGTGACCGAGTTCAAGTTCCTGTTCGTGAGCGGCCA<br>CTTCAAGGGCAACTACAAGGCCCAGCTGACCAGGCTGAACCGCAAAACCAACTGCAATGGC<br>GCCGTGCTGAGCGTGGAGGAGCTGCTGATCGGCGGCGAGATGATCAAAGCCGGCACCCTGA<br>CACTGGAGGAGGTGCGGCGCAAGTTCAACAACGGCGAGATCAACTTCTGATAA
TRAC - Site 3	55266	N-terminus	ATGGACTACAAAGACCATGACGGTGATTATAAAGATCATGACATCGATTACAAGGATGACG<br>ATGACAAGATGGCCCCCAAGAAGAAGAGGAAGGTCGGCATTCATGGGGTACCCGCCGCTAT<br>GGGACAGCTGGTGAAGAGCGAGCTGGAGGAGAAGAAGTCCGAGCTGCGGCACAAGCTGAA<br>GTACGTGCCCCACGAGTACATCGAGCTGATCGAGATCGCCAGGAACAGCACCCAGGACCGC<br>ATCCTGGAGATGAAGGTGATGGAGTTCTTCATGAAGGTGTACGGCTACAGGGGAAAGCACCT<br>GGGCGGAAGCAGAAAGCCTGACGGCGCCATCTATACAGTGGGCAGCCCCATCGATTACGGC<br>GTGATCGTGGACACAAAGGCCTACAGCGGCGGCTACAATCTGCCTATCGGCCAGGCCGACG<br>AGATGCAGAGATACGTGAAGGAGAACCAGACCCGGAATAAGCACATCAACCCCAACGAGT<br>GGTGGAAGGTGTACCCTAGCAGCGTGACCGAGTTCAAGTTCCTGTTCGTGAGCGGCCACTTC<br>AAGGGCAACTACAAGGCCCAGCTGACCAGGCTGAACCGCAAAACCAACTGCAATGGCGCCG<br>TGCTGAGCGTGGAGGAGCTGCTGATCGGCGGCGAGATGATCAAAGCCGGCACCCTGACACT<br>GGAGGAGGTGCGGCGCAAGTTCAACAACGGCGAGATCAACTTCAGCGGCGCCATCAGATGC<br>CACGACGAGTTCTGGTTCAGGCCCTTCCAGTGTCGAATCTGCATGCGTAACTTCAGTCAGTCC<br>TCCGACCTGTCCCGCCACATCCGCACCCACACCGGCGAGAAGCCTTTTGCCTGTGACATTTGT<br>GGGAGGAAATTTGCCCAGTCCGGCAACCGCACCACCCATACCAAGATACACACGCATCCCA<br>GGGCACCTATTCCCAAGCCCTTCCAGTGTCGAATCTGCATGCGTAACTTCAGTCGCTCCGCCA<br>ACCTGGCCCGCCACATCCGCACCCACACCGGCGAGAAGCCTTTTGCCTGTGACATTTGTGGGA<br>GGAAATTTGCCGACCGCTCCGCCCTGGCCCGCCATACCAAGATACACACGGGATCTCAGAAG<br>CCCTTCCAGTGTCGAATCTGCATGCGTAACTTCAGTCGCTCCGACGTGCTGTCCGAGCACATC<br>CGCACCCACACCGGCGAGAAGCCTTTTGCCTGTGACATTTGTGGGAGGAAATTTGCCAAGCA<br>CTCCACCCGCCGCGTGCATACCAAGATACACCTGCGGCAGAAGGACTGATAA
TRAC - Site 3	53853	C-terminus	ATGGACTACAAAGACCATGACGGTGATTATAAAGATCATGACATCGATTACAAGGATGACG<br>ATGACAAGATGGCCCCCAAGAAGAAGAGGAAGGTCGGCATCCACGGGGTACCCGCCGCTAT<br>GGCTGAGAGGCCCTTCCAGTGTCGAATCTGCATGCGTAACTTCAGTACCATGCACCAGCGCGT<br>GGAGCACATCCGCACCCACACCGGCGAGAAGCCTTTCGCCTGTGACATTTGTGGGAGGAAAT<br>TTGCCACCTCCGGCCACCTGTCCCGCCATACCAAGATACACACGGGCAGCCAAAAGCCCTTC<br>CAGTGTCGAATCTGCATGCGTAACTTCAGTCGCTCCGACCACCTGACCCAGCACATCCGCACC<br>CACACCGGCGAGAAGCCTTTTGCCTGTGACATTTGTGGGAGGAAATTTGCCGACTCCGCCAA<br>CCTGTCCCGCCATACCAAGATACACACGCACCCGCGCGCCCCGATCCCGAAGCCCTTCCAGT<br>GTCGAATCTGCATGCGTAACTTCAGTCAGTCCGGCTCCCTGACCCGCCACATCCGCACCCACA<br>CCGGCGAGAAGCCTTTTGCCTGTGACATTTGTGGGAGGAAATTTGCCGCCAAGTGGAACCTG<br>GACGCCCATACCAAGATACACCTGCGGGGATCCCAGCTGGTGAAGAGCGAGCTGGAGGAGA<br>AGAAGTCCGAGCTGCGGCACAAGCTGAAGTACGTGCCCCACGAGTACATCGAGCTGATCGA<br>GATCGCCAGGAACAGCACCCAGGACCGCATCCTGGAGATGAAGGTGATGGAGTTCTTCATG<br>AAGGTGTACGGCTACAGGGGAAAGCACCTGGGCGGAAGCAGAAAGCCTGACGGCGCCATCT<br>ATACAGTGGGCAGCCCCATCGATTACGGCGTGATCGTGGACACAAAGGCCTACAGCGGCGG<br>CTACAATCTGCCTATCGGCCAGGCCGACGAGATGGAGAGATACGTGGAGGAGAACCAGACC<br>CGGGATAAGCACCTCAACCCCAACGAGTGGTGGAAGGTGTACCCTAGCAGCGTGACCGAGT<br>TCAAGTTCCTGTTCGTGAGCGGCCACTTCAAGGGCAACTACAAGGCCCAGCTGACCAGGCTG<br>AACCACATCACCAACTGCAATGGCGCCGTGCTGAGCGTGGAGGAGCTGCTGATCGGCGGCG<br>AGATGATCAAAGCCGGCACCCTGACACTGGAGGAGGTGCGGCGCAAGTTCAACAACGGCGA<br>GATCAACTTCAGATCTTGATAA

Target Gene	SBSID	FokI Attachment Point	DNA Sequence
TRAC - Site 4	53885	N-terminus	ATGGACTACAAAGACCATGACGGTGATTATAAAGATCATGACATCGATTACAAGGATGACG<br>ATGACAAGATGGCCCCCAAGAAGAAGAGGAAGGTCGGCATTCATGGGGTACCCGCCGCTAT<br>GGGACAGCTGGTGAAGAGCGAGCTGGAGGAGAAGAAGTCCGAGCTGCGGCACAAGCTGAA<br>GTACGTGCCCCACGAGTACATCGAGCTGATCGAGATCGCCAGGAACAGCACCCAGGACCGC<br>ATCCTGGAGATGAAGGTGATGGAGTTCTTCATGAAGGTGTACGGCTACAGGGGAAAGCACCT<br>GGGCGGAAGCAGAAAGCCTGACGGCGCCATCTATACAGTGGGCAGCCCCATCGATTACGGC<br>GTGATCGTGGACACAAAGGCCTACAGCGGCGGCTACAATCTGCCTATCGGCCAGGCCGACG<br>AGATGCAGAGATACGTGAAGGAGAACCAGACCCGGAATAAGCACATCAACCCCAACGAGT<br>GGTGGAAGGTGTACCCTAGCAGCGTGACCGAGTTCAAGTTCCTGTTCGTGAGCGGCCACTTC<br>AAGGGCAACTACAAGGCCCAGCTGACCAGGCTGAACCGCAAAACCAACTGCAATGGCGCCG<br>TGCTGAGCGTGGAGGAGCTGCTGATCGGCGGCGAGATGATCAAAGCCGGCACCCTGACACT<br>GGAGGAGGTGCGGCGCAAGTTCAACAACGGCGAGATCAACTTCAGCGGCACTCCACACGAA<br>GTGGGAGTGTACACACTTAGGCCCTTCCAGTGTCGAATCTGCATGCGTAACTTCAGTCGCTCC<br>GACACCCTGTCCGAGCACATCCGCACCCACACCGGCGAGAAGCCTTTTGCCTGTGACATTTGT<br>GGGAGGAAATTTGCCACCTCCGGCTCCCTGACCCGCCATACCAAGATACACACGGGCAGCCA<br>AAAGCCCTTCCAGTGTCGAATCTGCATGCGTAACTTCAGTCGTAGTGACCACCTGAGCACCCA<br>CATCCGCACCCACACAGGCGAGAAGCCTTTTGCCTGTGACATTTGTGGGAGGAAATTTGCCA<br>CCAGCAGCAACCGCACAAAGCATACCAAGATACACACGCACCCGCGCGCCCCGATCCCGAA<br>GCCCTTCCAGTGTCGAATCTGCATGCGTAACTTCAGTCGCTCCGACAACCTGTCCGAGCACAT<br>CCGCACCCACACCGGCGAGAAGCCTTTTGCCTGTGACATTTGTGGGAGGAAATTTGCCTGGC<br>ACTCCTCCCTGCGCGTGCATACCAAGATACACCTGCGGCAGAAGGACTGATAA
TRAC - Site 4	53909	C-terminus	ATGGACTACAAAGACCATGACGGTGATTATAAAGATCATGACATCGATTACAAGGATGACG<br>ATGACAAGATGGCCCCCAAGAAGAAGAGGAAGGTCGGCATCCACGGGGTACCCGCCGCTAT<br>GGCTGAGAGGCCCTTCCAGTGTCGAATCTGCATGCGTAACTTCAGTCGCTCCGCCCACCTGTC<br>CCGCCACATCCGCACCCACACCGGCGAGAAGCCTTTTGCCTGTGACATTTGTGGGAGGAAAT<br>TTGCCGACCGCTCCGACCTGTCCCGCCATACCAAGATACACACGGGCAGCCAAAAGCCCTTC<br>CAGTGTCGAATCTGCATGCGTAACTTCAGTCGTAGTGACGTCCTGAGCGTACACATCCGCACC<br>CACACAGGCGAGAAGCCTTTTGCCTGTGACATTTGTGGGAGGAAATTTGCCCAGAACAACCA<br>CCGCATAACGCATACCAAGATACACACGGGATCTCAGAAGCCCTTCCAGTGTCGAATCTGCA<br>TGCGTAACTTCAGTCGCTCCGACGTGCTGTCCGAGCACATCCGCACCCACACCGGCGAGAAG<br>CCTTTTGCCTGTGACATTTGTGGGAGGAAATTTGCCTCCCCGTCCTCCCGCCGCACCCATACCA<br>AGATACACCTGCGGGGATCCCAGCTGGTGAAGAGCGAGCTGGAGGAGAAGAAGTCCGAGCT<br>GCGGCACAAGCTGAAGTACGTGCCCCACGAGTACATCGAGCTGATCGAGATCGCCAGGAAC<br>AGCACCCAGGACCGCATCCTGGAGATGAAGGTGATGGAGTTCTTCATGAAGGTGTACGGCTA<br>CAGGGGAAAGCACCTGGGCGGAAGCAGAAAGCCTGACGGCGCCATCTATACAGTGGGCAG<br>CCCCATCGATTACGGCGTGATCGTGGACACAAAGGCCTACAGCGGCGGCTACAATCTGCCTA<br>TCGGCCAGGCCGACGAGATGGAGAGATACGTGGAGGAGAACCAGACCCGGGATAAGCACC<br>TCAACCCCAACGAGTGGTGGAAGGTGTACCCTAGCAGCGTGACCGAGTTCAAGTTCCTGTTC<br>GTGAGCGGCCACTTCAAGGGCAACTACAAGGCCCAGCTGACCAGGCTGAACCACATCACCA<br>ACTGCAATGGCGCCGTGCTGAGCGTGGAGGAGCTGCTGATCGGCGGCGAGATGATCAAAGC<br>CGGCACCCTGACACTGGAGGAGGTGCGGCGCAAGTTCAACAACGGCGAGATCAACTTCAGA<br>TCTTGATAA
TRAC - Site 5	55248	C-terminus	ATGGACTACAAAGACCATGACGGTGATTATAAAGATCATGACATCGATTACAAGGATGACG<br>ATGACAAGATGGCCCCCAAGAAGAAGAGGAAGGTCGGCATCCACGGGGTACCCGCCGCTAT<br>GGCTGAGAGGCCCTTCCAGTGTCGAATCTGCATGCGTAACTTCAGTGACCAGTCCAACCTGCG<br>CGCCCACATCCGCACCCACACCGGCGAGAAGCCTTTTGCCTGTGACATTTGTGGGAGGAAAT<br>TTGCCACCTCCTCCAACCGCAAGACCCATACCAAGATACACACGGGCAGCCAAAAGCCCTTC<br>CAGTGTCGAATCTGCATGCGTAACTTCAGTCTGCAGCAGACCCTGGCCGACCACATCCGCACC<br>CACACCGGCGAGAAGCCTTTTGCCTGTGACATTTGTGGGAGGAAATTTGCCCAGTCCGGCAA<br>CCTGGCCCGCCATACCAAGATACACACGGGATCTCAGAAGCCCTTCCAGTGTCGAATCTGCA<br>TGCGTAACTTCAGTCGCCGCGAGGACCTGATCACCCACATCCGCACCCACACCGGCGAGAAG<br>CCTTTTGCCTGTGACATTTGTGGGAGGAAATTTGCCACCTCCTCCAACCTGTCCCGCCATACCA<br>AGATACACCTGCGGGGATCCCAGCTGGTGAAGAGCGAGCTGGAGGAGAAGAAGTCCGAGCT<br>GCGGCACAAGCTGAAGTACGTGCCCCACGAGTACATCGAGCTGATCGAGATCGCCAGGAAC<br>AGCACCCAGGACCGCATCCTGGAGATGAAGGTGATGGAGTTCTTCATGAAGGTGTACGGCTA<br>CAGGGGAAAGCACCTGGGCGGAAGCAGAAAGCCTGACGGCGCCATCTATACAGTGGGCAG<br>CCCCATCGATTACGGCGTGATCGTGGACACAAAGGCCTACAGCGGCGGCTACAATCTGCCTA<br>TCGGCCAGGCCGACGAGATGGAGAGATACGTGGAGGAGAACCAGACCCGGGATAAGCACC<br>TCAACCCCAACGAGTGGTGGAAGGTGTACCCTAGCAGCGTGACCGAGTTCAAGTTCCTGTTC<br>GTGAGCGGCCACTTCAAGGGCAACTACAAGGCCCAGCTGACCAGGCTGAACCACATCACCA<br>ACTGCAATGGCGCCGTGCTGAGCGTGGAGGAGCTGCTGATCGGCGGCGAGATGATCAAAGC<br>CGGCACCCTGACACTGGAGGAGGTGCGGCGCAAGTTCAACAACGGCGAGATCAACTTCAGA<br>TCTTGATAA

Target Gene	SBSID	FokI Attachment Point	DNA Sequence
TRAC - Site 5	55254	C-terminus	ATGGACTACAAAGACCATGACGGTGATTATAAAGATCATGACATCGATTACAAGGATGACGATGACAAGATGGCCCCCAAGAAGAAGAGGAAGGTCGGCATTCATGGGGTACCCGCCGCTATGGCTGAGAGGCCCTTCCAGTGTCGAATCTGCATGCGTAACTTCAGTCGCTCCGACCACCTGTCCACCCACATCCGCACCCACACCGGCGAGAAGCCTTTTGCCTGTGACATTTGTGGGAGGAAATTTGCCGACCGCTCCCACCTGGCCCGCCATACCAAGATACACACGGGCAGCCAAAAGCCCTTCCAGTGTCGAATCTGCATGCGTAAGTTTGCCCTGAAGCAGCACCTGAACGAGCATACCAAGATACACACGGGCGAGAAGCCCTTCCAGTGTCGAATCTGCATGCGTAACTTCAGTCAGTCCGGCAACCTGGCCCGCCACATCCGCACCCACACCGGCGAGAAGCCTTTTGCCTGTGACATTTGTGGGAGGAAATTTGCCCACAACTCCTCCCTGAAGGACCATACCAAGATACACCTGCGGGGATCCCAGCTGGTGAAGAGCGAGCTGGAGGAGAAGAAGTCCGAGCTGCGGCACAAGCTGAAGTACGTGCCCCACGAGTACATCGAGCTGATCGAGATCGCCAGGAACAGCACCCAGGACCGCATCCTGGAGATGAAGGTGATGGAGTTCTTCATGAAGGTGTACGGCTACAGGGGAAAGCACCTGGGCGGAAGCAGAAAGCCTGACGGCGCCATCTATACAGTGGGCAGCCCCATCGATTACGGCGTGATCGTGGACACAAAGGCCTACAGCGGCGGCTACAATCTGCCTATCGGCCAGGCCGACGAGATGCAGAGATACGTGAAGGAGAACCAGACCCGGAATAAGCACATCAACCCCAACGAGTGGTGGAAGGTGTACCCTAGCAGCGTGACCGAGTTCAAGTTCCTGTTCGTGAGCGGCCACTTCAAGGGCAACTACAAGGCCCAGCTGACCAGGCTGAACCGCAAAACCAACTGCAATGGCGCCGTGCTGAGCGTGGAGGAGCTGCTGATCGGCGGCGAGATGATCAAAGCCGGCACCCTGACACTGGAGGAGGTGCGGCGCAAGTTCAACAACGGCGAGATCAACTTCTGATAA

Supplementary Table 4 | ZFN design information for the ZFN pairs screened in **Supplementary Figure 19.** Base-skipping linker locations are shown between recognition helices and labeled as “1c”. The skipped base is also shown in the target site as a lowercase base.

Module archive from *Nat Methods* **9**, 588-5908[8]

			Recognition helices						
Pair	ZFN name	FokI attachment	F1	F2	F3	F4	F5	F6	Target site
1	1-1	N-terminal	RSDKLVR	DCRDLAR 1c	QSSNLVR	RSDDLVR	RSDKLVR	QRAHLER	GGAGGGGCGGAAaGCCGGG
	1-2	C-terminal	RSDDLVR	DPGNLVR	DPGHLVR	RSDNLVR	RSDELVR		GTGGAGGGCGACGCG
2	2-1	N-terminal	DCRDLAR	TSGNLVR	RSDELVR 1c	DPGNLVR	QSSNLVR		GAAGACaGTGGATGCC
	2-2	N-terminal	RSDELVR	QSSSLVR 1c	TSGHLVR	TSGNLVR	TSGELVR		GCTGATGGTgGTAGTG
3	3-1	N-terminal	QRAHLER	QSSNLVR	RSDKLVR	TSGELVR 1c	TSGSLVR	DPGHLVR	GGCGTTtGCTGGGGAAGGA
	3-2	C-terminal	TSGNLVR	TSGHLVR 1c	QSGDLRR	QSSNLVR	RSDKLVR		GGGGAAGCAgGGTGAT
4	4-1	N-terminal	TSGHLVR	TSGHLVR	QSGDLRR	RSDKLVR	RSDDLVR	RSDNLVR	GAGGCGGGGGCAGGTGGT
	4-2	C-terminal	QSGDLRR	TSGELVR 1c	TSGNLVR	TSGNLVR	TSGHLVR		GGTGATGATtGCTGCA
5	5-1	C-terminal	TSGELVR	TSGHLVR	TSGNLVR	TSGNLVR	QRAHLER		GGAGATGATGGTGCT
	5-2	C-terminal	DPGALVR	TSGSLVR	QSGDLRR 1c	TSGNLVR	TSGNLVR		GATGATtGCAGTTGTC
6	6-1	C-terminal	TSGSLVR	TSGNLVR	RSDNLVR	TSGELVR	RSDNLVR		GAGGCTGAGGATGTT
	6-1	N-terminal	QSGDLRR	RSDKLVR	TSGNLVR	QSSNLVR	RSDKLVR		GGGGAAGATGGGGCA
7	7-1	N-terminal	TSGELVR	TSGELVR	TSGELVR	TSGELVR	TSGSLVR	TSGSLVR	GTTGTTGCTGCTGCTGCT
	7-2	N-terminal	QSSNLVR	RSDKLVR	QSSNLVR	RSDELVR	QSSNLVR		GAAGTGGAAGGGGAA
8	8-1	N-terminal	DCRDLAR	QRAHLER	DPGNLVR	QRAHLER	QSGDLRR	RSDDLVR	GCGGCAGGAGACGGAGCC
	8-2	C-terminal	RSDNLVR	RSDNLVR	QSSNLVR	QRAHLER 1c	RSDELVR	QSGDLRR	GCAGTGgGGAGAAGAGGAG
9	9-1	N-terminal	RSDNLVR	TSGHLVR	QSSNLVR	RSDKLVR	QSSNLVR	RSDKLVR	GGGGAAGGGGAAGGTGAG
	9-2	N-terminal	QRAHLER	QSGDLRR	QSSNLVR	QSGDLRR 1c	DPGNLVR	QSSNLVR	GAAGACaGCAGAAGCAGGA
10	10-1	N-terminal	RSDKLVR	TSGNLVR	QSGDLRR 1c	RSDNLVR	TSGSLVR		GTTGAGaGCAGATGGG
	10-2	C-terminal	TSGNLVR	TSGSLVR	RSDKLVR	TSGNLVR	TSGSLVR		GTTGATGGGGTTGAT
11	11-1	C-terminal	RSDKLVR	QSSNLVR	RSDKLVR 1c	TSGNLVR	TSGELVR		GCTGATtGGGGAAGGG
	11-2	C-terminal	TSGELVR	RSDNLVR	RSDKLVR	TSGSLVR	TSGHLVR	RSDNLVR	GAGGGTGTTGGGGAGGCT
12	12-1	C-terminal	QSSNLVR	RSDKLVR 1c	TSGNLVR	TSGELVR	TSGSLVR		GTTGCTGATtGGGGAA
	12-2	C-terminal	TSGELVR	RSDNLVR	RSDKLVR	TSGSLVR	TSGHLVR		GGTGTTGGGGAGGCT
13	13-1	N-terminal	DCRDLAR	QRAHLER	DPGHLVR	RSDKLVR	TSGHLVR		GGTGGGGGCGGAGCC
	13-2	N-terminal	RSDKLVR	RSDDLVR	RSDKLVR	DPGNLVR 1c	DPGHLVR	RSDDLVR	GCGGGCaGACGGGGCGGGG
14	14-1	C-terminal	DPGHLVR	RSDDLVR	TSGELVR	DPGHLVR	RSDKLVR	QRAHLER	GGAGGGGGCGCTGCGGGC
	14-2	N-terminal	DPGHLVR	RSDDLVR	DPGHLVR	DCRDLAR	QRAHLER	QRAHLER	GGAGGAGCCGGCGCGGGC
15	15-1	C-terminal	QRAHLER	TSGHLVR	RSDELVR	RSDNLVR	RSDELVR	TSGNLVR	GATGTGGAGGTGGGTGGA
	15-2	N-terminal	DPGHLVR	TSGHLVR	RSDDLVR	TSGNLVR	RSDKLVR		GGGGATGCGGGTGGC

Module archive from *PNAS* **96**, 2758-2763[9]

			Recognition helices						
Pair	ZFN name	FokI attachment	F1	F2	F3	F4	F5	F6	Target site
1	1-1	N-terminal	QRGTLTQ	QRGDLTR	RSDNLTK	QRGTLTQ	QRGNLTR		GAAACATAGGCAATA
	1-2	C-terminal	QRGHLTQ	QRGNLTR	QRGTLVE	QRGNLTR	RSDHLTQ		AGGGAACCAGAAAGA
2	2-1	N-terminal	QLSNLTR	RSDTLVR	QSGHLTR	QRGTLVE	RKRSLTR		GTGACAGGAGTGGAA
	2-2	C-terminal	QRGTLER	DRGNLTR 1c	RSDTLKE	RSSNLTR	QRGTLVE	QRGNLTR	GAACTAGAGCTGtGACGTA
3	3-1	C-terminal	QRGTLTQ	QRGNLTR	RSDNLTQ 1c	QRGTLTQ	QRGELTR		GTAATAgAAGGAAACA
	3-2	C-terminal	QRSTRKR	RSDTLKQ	AAGDLTR	RSDTLKG	QRCNLTR		GAATTGGCTATGGCA
4	4-1	C-terminal	RSDTLRR	IRFHLTR 1c	QRGHLTR	QRGTLKQ	AAGNLTR		GATATAGGAaGGTGTG
	4-2	C-terminal	QRGNLKQ	AAGNLTR	QRGTLKG	DRCNLTR	QRGTLKQ	AAGHLTR	GGTACAGACTCAGATAAA
5	5-1	N-terminal	RSDHLTR	QRGTLTQ	QRGELTR	QRGHLTQ	QRGNLTR		GAATGAGTAACAGGG
	5-2	C-terminal	DRSDLTR	QRGTLKQ	AAGNLTR	DKGTLVE	QRGNLTR		GAACCTGATACAGCC
6	6-1	C-terminal	RSDTLKG	DRCHLTR	DKGTLVE	QRGNLTR	QSSHLKQ		AGAGAACCTGGCTTG
	6-1	N-terminal	YRQSLTR	QRGTLTQ	QRGHLTR	QRGTLKG	DRCNLTR		GACTTAGGAATAGTT
7	7-1	N-terminal	RSDTLKG	QRCHLTR	RSDTLKE	ARRNLTR	LSFNLTR		GATGGCATGGGATTG
	7-2	C-terminal	QRGHLTQ	QRGNLTR	QRGTLKQ	DKGNLTR	RSDALTQ		ATGGACACAGAAAGA
8	8-1	N-terminal	QRGTLRR	IRFHLTR	QRGTLRR	IRFHLTR	QLSNLTR		GAAGGTGCAGGTGCA
	8-2	N-terminal	RSDTLKG	QRCNLTR 1c	DKGTLTQ	QRGNLTR	QRGHLTQ	QRGNLTR	GAAAGAGAAATTaGAATTG
9	9-1	C-terminal	QRGTLTQ	QRGELTR	YRQSLTR	RSDTLVE	RKRSLTR		GTGATGGTTGTAATA
	9-2	N-terminal	QRGTLKE	ARRNLTR 1c	RSDTLAR	RAEHLTR	RSDALRK		TTGGGGGTGgGGCATA
10	10-1	N-terminal	QRGTLKE	RSSHLTR	QRGTLVE	QRGNLTR	RSDNLTQ		AAGGAACCAGGGCTA
	10-2	N-terminal	RSDTLKE	KGCNLTR	QRGTLKG	QRCHLTR	LSFNLTR		GATGGATAAGAGTTG
11	11-1	C-terminal	RSDTLMV	DRSNLTR	QRGHLTR	QRGTLVR	QSGHLTR		GGAGCAGGAGACCTG
	11-2	N-terminal	QRGNLTQ	QRGNLTR	RSDHLTT	RSDNLTQ	QRGNLTR		GAAAAGTGGGAAAAA
12	12-1	N-terminal	RSDALTQ	QRGTLVE	RKRHLTR 1c	QRGHLTQ	QRGNLTR		GAATGAaGGGACAATG
	12-2	N-terminal	QRGTLRE	QRGHLTR	QSGALTR	RSDTLVE	RKRSLTR		GTGATGGTAGGACTA
13	13-1	C-terminal	QRGTLVR	QSGHLTR 1c	RSDALRK	QRGTLKQ	AAGNLTR		GATACATTGgGGAGTA
	13-2	C-terminal	DRSALAR	QRGTLAR	RAEHLTR	QRGTLKG	DRCNLTR		GACTTAGGGGCAGTC
14	14-1	N-terminal	QRGTLVR	QSGHLTR	RSDHLTQ	QRGTLKE	RSSHLTR		GGGCCAAGGGGAGCA
	14-2	N-terminal	RSDHLTQ	QRGNLTR 1c	YRQSLTR	RSDTLKQ	AAGNLTR		GATATGGTTtGAAAGG
15	15-1	C-terminal	RSDTLMV	DRSNLTR	QRGTLKQ	DKRSLTR 1c	QRGTLTQ	QRGHLTR	GGAATAaGTCACAGACCTG
	15-2	C-terminal	QRGTLTQ	QRGHLTR	RSDNLTQ	RSDTLMR	IRSNLTR		GATGTGAAGGGAATA

Supplementary Table 5 | Primers used to amplify genomic loci for on- and off-target analysis of T-cells treated with TRAC ZFNs. Primer sequences are 5’ to 3’. On-target primers are highlighted in green.

TRAC Site 1 : 55204:53759

Locus	Genomic Coordinates (hg38)	Forward Primer	Reverse Primer
TRAC 1	chr14:22547702 (On-Target)	ACACGACGCTCTTCCGATCTNNNNCAAACAAATGTGTCACAAAGTAAGG	GACGTGTGCTCTTCCGATCTGGAAGAAGGTGTCTTCTGGAAT
1-1	chr20:34112234	ACACGACGCTCTTCCGATCTNNNNGAAGTCAGACGGGTCAGCC	GACGTGTGCTCTTCCGATCTCAACGCTTGGAGGAGAGGG
1-2	chr21:34950722	ACACGACGCTCTTCCGATCTNNNNGAATCACTTGAAACCAGAGG	GACGTGTGCTCTTCCGATCTCCTCTCTGAAATGTAACTAAGTCA
1-3	chr2:200751558	ACACGACGCTCTTCCGATCTNNNNCCACCCAGAATTTTGTATCTGCT	GACGTGTGCTCTTCCGATCTATCTTTTCAGCTGCTCGTTTTG
1-4	chr15:90295664	ACACGACGCTCTTCCGATCTNNNNCTCCGAAGATCTTTGTGAGGTG	GACGTGTGCTCTTCCGATCTAAAGAAAGGCATGCAGAGATTG
1-5	chr8:78155198	ACACGACGCTCTTCCGATCTNNNNTTGGGGATGGGTGACAAATCA	GACGTGTGCTCTTCCGATCTAATCAGGGTCAACTTGTTGA
1-6	chr15:75786878	ACACGACGCTCTTCCGATCTNNNNTTTATGAAGTGAGCCTGGACGA	GACGTGTGCTCTTCCGATCTAAAGAATGACACACAGGGGTTG
1-7	chr9:128257214	ACACGACGCTCTTCCGATCTNNNNCTTCACCTCATCATTCTCGTCAG	GACGTGTGCTCTTCCGATCTAGTGTGGAGCCTGCCCAAG
1-8	chr15:75163594	ACACGACGCTCTTCCGATCTNNNNAGAGGGTTGATTATTTGCATATT	GACGTGTGCTCTTCCGATCTCTGGTGGTGCATGTCTGT
1-9	chr22:19741288	ACACGACGCTCTTCCGATCTNNNNGATGTCTGCCTGGGGTGTT	GACGTGTGCTCTTCCGATCTAGTGGTTCAAGACTGCAGTGAG
1-10	chr1:31595940	ACACGACGCTCTTCCGATCTNNNNAACTCCCACTTTCCCATTCTGT	GACGTGTGCTCTTCCGATCTAATGATACCTGTGAAAGCATGAT
1-11	chr2:188294076	ACACGACGCTCTTCCGATCTNNNNGCAGTTGGATATGACCGAGGAA	GACGTGTGCTCTTCCGATCTTGCAAAGTATGATCAGCACAAAC
1-12	chr12:7907436	ACACGACGCTCTTCCGATCTNNNNCTGCCACATCAGTCTTTTCTTTT	GACGTGTGCTCTTCCGATCTACTGATGCAAGACAACTGCTT
1-13	chr12:57745830	ACACGACGCTCTTCCGATCTNNNNTCATTGGAGTGGCTGCTTG	GACGTGTGCTCTTCCGATCTGACTTGGTGGTGGTTGACAG
1-14	chr12:121302880	ACACGACGCTCTTCCGATCTNNNNGAATGGCGTGAACCCCAGG	GACGTGTGCTCTTCCGATCTTTGCCTGGCTTGGTGAATCT
1-15	chr1:12434486	ACACGACGCTCTTCCGATCTNNNNGTGCTCTTAAAGTTTGGTGGTTG	GACGTGTGCTCTTCCGATCTAGTAGGTGTAACTAAAACCCAGT
1-16	chr1:51934180	ACACGACGCTCTTCCGATCTNNNNCCCCAGCTAGCTATGTGATCA	GACGTGTGCTCTTCCGATCTCAAGCCATGGAAGAGCTTTTGG
1-17	chr1:200969780	ACACGACGCTCTTCCGATCTNNNNTTCTATTTCCTACTTCCCTGCA	GACGTGTGCTCTTCCGATCTTCTGTTTCTTCTTTGATACCTTTGT
1-18	chr6:13566180	ACACGACGCTCTTCCGATCTNNNNATCCCCATCCACTGCTCAAC	GACGTGTGCTCTTCCGATCTACGGCCTCTAAGAATATAAACCA
1-19	chr2:239999000	ACACGACGCTCTTCCGATCTNNNNGAACAGAGATAGGCAGCTACCA	GACGTGTGCTCTTCCGATCTCACAGCCTCCTCCCCTCTG
1-20	chr19:47348978	ACACGACGCTCTTCCGATCTNNNNACTTGGGAGGCGGAGATT	GACGTGTGCTCTTCCGATCTCCCAAAGAGAGAGCTGGTC
1-21	chrX:109959710	ACACGACGCTCTTCCGATCTNNNNAAAGGGAGATAGCAGTGTAGTAC	GACGTGTGCTCTTCCGATCTCTTACACTTGTCCTTTGAGCCA
1-22	chr14:53202846	ACACGACGCTCTTCCGATCTNNNNAAATAACTTCTTGGGAGCTCTGTG	GACGTGTGCTCTTCCGATCTTGTGTTGCATTGGTTTTGTG
1-23	chr19:55115786	ACACGACGCTCTTCCGATCTNNNNGAGACTAGGAAGGAGGAGGCC	GACGTGTGCTCTTCCGATCTGGTTAATGTGGCTCTGGTTCT

TRAC Site 2: 53785:55229

Locus	Genomic Coordinates (hg38)	Forward Primer	Reverse Primer
TRAC 2	chr14:22547708 (On-Target)	ACACGACGCTCTTCCGATCTNNNNCAAACAAATGTGTCACAAAGTAAGG	GACGTGTGCTCTTCCGATCTGGAAGAAGGTGTCTTCTGGAAT
2-1	chr10:48510064	ACACGACGCTCTTCCGATCTNNNNTGAATTTTGGAGTTAGTCAGCGG	GACGTGTGCTCTTCCGATCTATGCCCTTGGTTTTAAAGAGATGA
2-2	chr14:74911492	ACACGACGCTCTTCCGATCTNNNNCAGAGACTGCTGCTGTTAGGG	GACGTGTGCTCTTCCGATCTCAGATTCTACAGGGGCAGTGG
2-3	chr7:29537720	ACACGACGCTCTTCCGATCTNNNNCAGACAGAAATGAAATCCAGTA	GACGTGTGCTCTTCCGATCTTACAGACCTAAATTTCATCTCCT
2-4	chr5:134459566	ACACGACGCTCTTCCGATCTNNNNCTGGTTGCTCTTCCTCACTCCC	GACGTGTGCTCTTCCGATCTCAGAAGCACAGGATGGGAG
2-5	chr3:143018376	ACACGACGCTCTTCCGATCTNNNNTAACGTGGTCTTTTGTATCTGGC	GACGTGTGCTCTTCCGATCTCCATCTACTAAGTAATGCATGGATA
2-6	chr11:68896910	ACACGACGCTCTTCCGATCTNNNNACCCCTTGTGAGCACCTC	GACGTGTGCTCTTCCGATCTTTGGGCGTTCTCTGCAGTCTTG
2-7	chr12:2298932	ACACGACGCTCTTCCGATCTNNNNGCCACTCCAATCTTGCAGGA	GACGTGTGCTCTTCCGATCTATTCCCACACACATACTCAAACC
2-8	chr7:139562630	ACACGACGCTCTTCCGATCTNNNNAAGCAAAACGGGATGGAAACTT	GACGTGTGCTCTTCCGATCTTGTCATCCACCTCACCACGG
2-9	chr16:84138904	ACACGACGCTCTTCCGATCTNNNNACGGGACAATTATGGTCAAAACT	GACGTGTGCTCTTCCGATCTATTGCCCTTTACACTGCTAGGT
2-10	chr14:27506760	ACACGACGCTCTTCCGATCTNNNNCAAGGTGCTGGCAAGGGTA	GACGTGTGCTCTTCCGATCTAGCACACATATATTCATGCACCC
2-11	chr17:80188398	ACACGACGCTCTTCCGATCTNNNNTAGGAGGAAGGGTGAGAAATGC	GACGTGTGCTCTTCCGATCTTCACAGGAGGAAACCATGTTG
2-12	chr4:26571374	ACACGACGCTCTTCCGATCTNNNNTCAATACCGTTACCCTTTATTATGT	GACGTGTGCTCTTCCGATCTGTGGTTGCTGAAATTCTACACA
2-13	chr17:36523328	ACACGACGCTCTTCCGATCTNNNNTATCCTAGGACAGATGGGAATCT	GACGTGTGCTCTTCCGATCTTACCCTTCCTAGAGCTCTCAGA
2-14	chr6:148546158	ACACGACGCTCTTCCGATCTNNNNGATACGCAGAGGACTTGGATCAG	GACGTGTGCTCTTCCGATCTACTAAATGTGCCTCAGGAAGC
2-15	chr18:7456684	ACACGACGCTCTTCCGATCTNNNNGGTTTGCCTAATTACATTTTCACA	GACGTGTGCTCTTCCGATCTCTGCCTTCTGACAAATAACCTGT
2-16	chr9:120508618	ACACGACGCTCTTCCGATCTNNNNGTATGGTGCTAGCTGTGATGAC	GACGTGTGCTCTTCCGATCTGGGGAATGGGAGGTGGGAAT
2-17	chr1:161160086	ACACGACGCTCTTCCGATCTNNNNGGGGAGGGAATGGATCGTAAG	GACGTGTGCTCTTCCGATCTAGTGTAGATGCAAGAATAACAGCC
2-18	chr14:22546294	ACACGACGCTCTTCCGATCTNNNNGATGTCTCTCCCCTCTGACCAT	GACGTGTGCTCTTCCGATCTACCAAAGAAATCAGCCCCTG
2-19	chr14:22546618	ACACGACGCTCTTCCGATCTNNNNGGCAGGAGGTCGGAAAGAATAA	GACGTGTGCTCTTCCGATCTCTTCACCCTCAGAAAGCAGGAG
2-20	chr20:47196592	ACACGACGCTCTTCCGATCTNNNNGCTCATCTGCAACCACCAG	GACGTGTGCTCTTCCGATCTGCAGACGCCTTGAGAATCCTTT
2-21	chr7:2636610	ACACGACGCTCTTCCGATCTNNNNTACCTACAGATGACAGAAGCGG	GACGTGTGCTCTTCCGATCTGGGTCCCATTCGCTGTACAC
2-22	chr17:55642076	ACACGACGCTCTTCCGATCTNNNNACTTCATCTTCAGTGAACCTACA	GACGTGTGCTCTTCCGATCTTAGTACTACAATCTACTTTCCTAGC
2-23	chr18:70127566	ACACGACGCTCTTCCGATCTNNNNTGACCTTACACTGACCTGTTTAGT	GACGTGTGCTCTTCCGATCTGAAGTTAGGGCTGCTGTCAC

TRAC Site 3: 55266:53853

Locus	Genomic Coordinates (hg38)	Forward Primer	Reverse Primer
TRAC 3	chr14:22549694 (On-Target)	ACACGACGCTCTTCCGATCTNNNNAGTCAGCCTGCTCTGGAT	GACGTGTGCTCTTCCGATCTCAGGGTTATTGCGGGTTCA
3-1	chr2:10072832	ACACGACGCTCTTCCGATCTNNNNTAGTCTCGTTCTGTTGCTCACC	GACGTGTGCTCTTCCGATCTCTCCTCCACCTGCACTGCT
3-2	chr8:143573262	ACACGACGCTCTTCCGATCTNNNNCAGTCAGCTCCCCACTGG	GACGTGTGCTCTTCCGATCTACCGCTCCTCTGGTGTCTTCA
3-3	chr21:25838602	ACACGACGCTCTTCCGATCTNNNNAAATCTGATGTGATCTCGGCTAAAA	GACGTGTGCTCTTCCGATCTTGAGAGGGGTCCATGAGTCA
3-4	chr3:185380650	ACACGACGCTCTTCCGATCTNNNNAGACATTCGCAGTCAGAAATGTT	GACGTGTGCTCTTCCGATCTTCTAGGCACATTAAAAGTCTGAGA
3-5	chr3:113729320	ACACGACGCTCTTCCGATCTNNNNGCTGGTCTTGAAATTATGGCCT	GACGTGTGCTCTTCCGATCTGAAACACTTCCAAACTTTCTTT
3-6	chr2:127183046	ACACGACGCTCTTCCGATCTNNNNAGTGTCCCTCTGATAAGTTCCTTT	GACGTGTGCTCTTCCGATCTGTTCATGAGCGAGCCAAGG
3-7	chr3:129120744	ACACGACGCTCTTCCGATCTNNNNTTTCTATCAAACTCCAGCTG	GACGTGTGCTCTTCCGATCTTTTTGTCAGAATTTCCGGCCT
3-8	chr10:24893978	ACACGACGCTCTTCCGATCTNNNNTGAACTTCAGGGAAATGACACAA	GACGTGTGCTCTTCCGATCTGAGGACTACAGTAATGTTATCAGGA
3-9	chr8:80297348	ACACGACGCTCTTCCGATCTNNNNGGAGCAAAGAAGGAAGAGAAAGC	GACGTGTGCTCTTCCGATCTAGTCTTCCTGATACCTTCTGTGTA
3-10	chr17:6710544	ACACGACGCTCTTCCGATCTNNNNAAACTTAGAAGCAGTAGGTGGC	GACGTGTGCTCTTCCGATCTCTTCTAAATAGTGGCGCGGAGG
3-11	chr16:14135786	ACACGACGCTCTTCCGATCTNNNNCAGTAACTGGTCTCTGTCAGA	GACGTGTGCTCTTCCGATCTTGTAGAGGGCATGACACAG
3-12	chr13:111389164	ACACGACGCTCTTCCGATCTNNNNAGGATGCCAGGGGAAGGA	GACGTGTGCTCTTCCGATCTGTTACTTTGGCGTTGCTTGGTT
3-13	chr15:66504312	ACACGACGCTCTTCCGATCTNNNNACTACGTATTTAGTCCAACGCA	GACGTGTGCTCTTCCGATCTTGTGTACTGCTTTTCCACAAATAGT
3-14	chr17:30104782	ACACGACGCTCTTCCGATCTNNNNAGTAGCACTCAACCGTTAATAGG	GACGTGTGCTCTTCCGATCTGAAGCTCCGAAAGTGTCTGGAC
3-15	chr2:135447400	ACACGACGCTCTTCCGATCTNNNNGTGCCTGGCCAACACTTTATT	GACGTGTGCTCTTCCGATCTGAGAATGCTGAAAAGCCACA
3-16	chr17:62774682	ACACGACGCTCTTCCGATCTNNNNAGGATGCACTGCTCACCGTT	GACGTGTGCTCTTCCGATCTATTTGGGAATCTGGAGGTGAGG
3-17	chr10:48510062	ACACGACGCTCTTCCGATCTNNNNATGAATTTTGGAGTTAGTCAGCG	GACGTGTGCTCTTCCGATCTTGCCCTTGGTTTTAAAGAGATGAA
3-18	chr14:22548690	ACACGACGCTCTTCCGATCTNNNNCTTCAAGCAGGGGAGGGACAC	GACGTGTGCTCTTCCGATCTGTGAGCCAAGATGCACCATT
3-19	chr1:213562852	ACACGACGCTCTTCCGATCTNNNNTCCGCTTTCCAAGTAGCTATGA	GACGTGTGCTCTTCCGATCTAAGAGGACCACTTCAGGCCAG
3-20	chr14:96762866	ACACGACGCTCTTCCGATCTNNNNTGTTTTCCAGCTAGCGGG	GACGTGTGCTCTTCCGATCTCTTTGCCATTGAGAGTGGTCAG
3-21	chr20:61650104	ACACGACGCTCTTCCGATCTNNNNTCTCCTGGGCACATTTCCTAAA	GACGTGTGCTCTTCCGATCTGGTATAAGAATATTGATGGGCCGG
3-22	chrX:3550058	ACACGACGCTCTTCCGATCTNNNNCTTATCAGAATCCCAACCTCCATG	GACGTGTGCTCTTCCGATCTATGGGGCCACTCTTCTGAAG
3-23	chrX:100668212	ACACGACGCTCTTCCGATCTNNNNATAAAAGAACAGGCTAGGTTACT	GACGTGTGCTCTTCCGATCTTCTTTAGAAGCCTCCTAAGTAGCT

TRAC Site 4: 53885:53909

Locus	Genomic Coordinates (hg38)	Forward Primer	Reverse Primer
TRAC 4	chr14:22550620 (On-Target)	ACACGACGCTCTTCCGATCTNNNNAAGCCCATAACCGCTGTG	GACGTGTGCTCTTCCGATCTCACTCCCAGCTTCAAGGC
4-1	chr3:19931480	ACACGACGCTCTTCCGATCTNNNNCTGCATGTGTGTGAGATCAGAT	GACGTGTGCTCTTCCGATCTTCTCACACTGCTTTCTGGTTTC
4-2	chr16:85765916	ACACGACGCTCTTCCGATCTNNNNAGGGCAAAGAGGAGGAAGCTCT	GACGTGTGCTCTTCCGATCTCAGAGGTAGGAGGGAAGGTG
4-3	chr22:40240156	ACACGACGCTCTTCCGATCTNNNNAAACTCCTGACCTCATGATTTGC	GACGTGTGCTCTTCCGATCTAGGTGAAGAGCGAATACAACAG
4-4	chr17:44006768	ACACGACGCTCTTCCGATCTNNNNCTCTTTAGCAACAAGGGTGAGG	GACGTGTGCTCTTCCGATCTGAGAAGCCGCTCCTCCTGAC
4-5	chr14:22549696	ACACGACGCTCTTCCGATCTNNNNCCTGCTCTGGATGCTGAAAGA	GACGTGTGCTCTTCCGATCTTTCATCACTTCCGCAATCCTGT
4-6	chr12:48296828	ACACGACGCTCTTCCGATCTNNNNCTAGAAAGGTGCGCATGTTCCG	GACGTGTGCTCTTCCGATCTGAAGCTTCTATCTCATGGCGC
4-7	chr22:42745580	ACACGACGCTCTTCCGATCTNNNNTTAGCTTCCTTAGACTTTTGGGG	GACGTGTGCTCTTCCGATCTCTTTATTTGCTGCCTCTCCTCG
4-8	chr7:106290510	ACACGACGCTCTTCCGATCTNNNNGTGAAACCTTGAACTGCAGCAA	GACGTGTGCTCTTCCGATCTAGGAAAGTCATGTGGGCAAATG
4-9	chr1:207819138	ACACGACGCTCTTCCGATCTNNNNTTTTGTAGCTTTCATGTTGACA	GACGTGTGCTCTTCCGATCTGTGACGGCTGCTTTGTTGTTT
4-10	chr6:20550408	ACACGACGCTCTTCCGATCTNNNNGGCCCGTCTTAGTTATTTCTATCAG	GACGTGTGCTCTTCCGATCTTGCCGGCATGCTGAAAAG
4-11	chr14:22972748	ACACGACGCTCTTCCGATCTNNNNGAAGAAGGCCAAGAAATAACTCATT	GACGTGTGCTCTTCCGATCTGGCAACCAAATTTAAGCTCCTC
4-12	chr19:14384498	ACACGACGCTCTTCCGATCTNNNNTGGTGAGTCATTCCTTCCCTTAG	GACGTGTGCTCTTCCGATCTCCGATCTGGGAATACTGGGGAC
4-13	chr15:41544018	ACACGACGCTCTTCCGATCTNNNNTACATTGCAGCCTATCCTCCAG	GACGTGTGCTCTTCCGATCTAGCTGCGACTCGATGATTCC
4-14	chr19:1872652	ACACGACGCTCTTCCGATCTNNNNCTGTTCTGAGCTCCACTTCCG	GACGTGTGCTCTTCCGATCTCTCAGAAATAAGGTCCCGGGA
4-15	chr18:51458202	ACACGACGCTCTTCCGATCTNNNNTCTTGGACACAGTAATTCCTTC	GACGTGTGCTCTTCCGATCTGTGAAAAGTCCTTGTAAACTATA
4-16	chr19:58546220	ACACGACGCTCTTCCGATCTNNNNTGAGAAGGCTTATCAGGGAGTT	GACGTGTGCTCTTCCGATCTTCCCTAGCAGCATCTTATACCAG
4-17	chr14:22550220	ACACGACGCTCTTCCGATCTNNNNGCAATGGTCCTGTCTCTCAAGA	GACGTGTGCTCTTCCGATCTCCAGATGGGCCCCTTAGTG
4-18	chr22:16602874	ACACGACGCTCTTCCGATCTNNNNCCATTTGGCTTCTTCCTTCCC	GACGTGTGCTCTTCCGATCTTACGTCTCCAAAACTTAGGCAGG
4-19	chr1:21195332	ACACGACGCTCTTCCGATCTNNNNACTGGGCAAGGAGAGGGTA	GACGTGTGCTCTTCCGATCTAGTTTCAAGATTTACCACCAGAGG
4-20	chr7:152435000	ACACGACGCTCTTCCGATCTNNNNGGCTACGTCTCCAAAACTTAGG	GACGTGTGCTCTTCCGATCTGAAGCCATTTGACTTCTTCCTCC
4-21	chr8:129726458	ACACGACGCTCTTCCGATCTNNNNGGAGAAAGACATGGAAGAGGGG	GACGTGTGCTCTTCCGATCTCAGCATTGTCAAACAGCAGGATG
4-22	chr18:268540	ACACGACGCTCTTCCGATCTNNNNGAGCTTAAGATGCGGAGTGGTG	GACGTGTGCTCTTCCGATCTCGGTCATTATTTCAGTGGAGCTT
4-23	chr1:37807680	ACACGACGCTCTTCCGATCTNNNNCTTCCCAGCAGCATGTAGTTC	GACGTGTGCTCTTCCGATCTGGTTTCCGGTGACAGGCTTG
4-24	chr3:8426848	ACACGACGCTCTTCCGATCTNNNNGAATTCCATCCTCGCCCCACAA	GACGTGTGCTCTTCCGATCTTTTTCCAACCATCACAGCAACT
4-25	chr7:134661812	ACACGACGCTCTTCCGATCTNNNNCTGGAAGAGCTAGGCCAGGAAT	GACGTGTGCTCTTCCGATCTCAGAGCCTCACTTGTTCTTCAC
4-26	chr22:18563488	ACACGACGCTCTTCCGATCTNNNNCGCGATCATAGCTCTAATTTAT	GACGTGTGCTCTTCCGATCTTGGAAAACATAGTGAGACCTC

TRAC Site 5: 55248:55254

Locus	Genomic Coordinates (hg38)	Forward Primer	Reverse Primer
TRAC 5	chr14:22550624 (On-Target)	ACACGACGCTCTTCCGATCTNNNNAAGCCCATAACCGCTGTG	GACGTGTGCTCTTCCGATCTCACTCCCAGCTTCAAGGC
5-1	chr2:102757248	ACACGACGCTCTTCCGATCTNNNNACCTGCTACCTATTCTTTCCCC	GACGTGTGCTCTTCCGATCTTTGTTTAAGACTTTCAGCCTGTCC
5-2	chr10:69524212	ACACGACGCTCTTCCGATCTNNNNATGGTTCAGTATTAGGAGATTTAGT	GACGTGTGCTCTTCCGATCTGGTCTTGAACTACTGGCTTCCA
5-3	chr1:14638584	ACACGACGCTCTTCCGATCTNNNNGAGCTGAGATTGCACCACG	GACGTGTGCTCTTCCGATCTGGTCTTCCCCTTAGAGTTCTCAA
5-4	chr14:22549802	ACACGACGCTCTTCCGATCTNNNNTGATGAACCCGCAATAACCCTG	GACGTGTGCTCTTCCGATCTGTGAGAGGGATTCAGGAGAGAC
5-5	chr2:55427510	ACACGACGCTCTTCCGATCTNNNNAGTCAGCTGAGATCACGG	GACGTGTGCTCTTCCGATCTGTAGCTGGCGTTATAGACATGTG
5-6	chr9:127793116	ACACGACGCTCTTCCGATCTNNNNACATTTGTTCATGCAATTAAGCCAT	GACGTGTGCTCTTCCGATCTAGGATCCCTTGAGCTGAGG
5-7	chr9:13565922	ACACGACGCTCTTCCGATCTNNNNTTTCTACTTACATTTCCTGGCCATG	GACGTGTGCTCTTCCGATCTATTGCTCTAGTACCACAGCACT
5-8	chr14:85441244	ACACGACGCTCTTCCGATCTNNNNAGGCACCCTCCACCACGG	GACGTGTGCTCTTCCGATCTTGATAAACATTGGCCAGGCACG
5-9	chr1:46514122	ACACGACGCTCTTCCGATCTNNNNGGCTAACATGTGAAACTAATAAT	GACGTGTGCTCTTCCGATCTCTTCCACCTCCCAGATTG
5-10	chr19:14202338	ACACGACGCTCTTCCGATCTNNNNGAAAAGAAGACACACATGCCTTC	GACGTGTGCTCTTCCGATCTGCGGGAGGATTGCTTGAAT
5-11	chrX:23799240	ACACGACGCTCTTCCGATCTNNNNACTGCTGGGATTATAGGG	GACGTGTGCTCTTCCGATCTCTTTAGTAAGGTGGGAGAAGGG
5-12	chr1:216854368	ACACGACGCTCTTCCGATCTNNNNAAGTGGAAGTTGCAGTGAGT	GACGTGTGCTCTTCCGATCTTAGAAATACATCTAGGGAGTTTT
5-13	chr4:70823174	ACACGACGCTCTTCCGATCTNNNNGTGAGCCCAGACTGCATC	GACGTGTGCTCTTCCGATCTTTGGTCATGGCTGGTCTTG
5-14	chr10:23114076	ACACGACGCTCTTCCGATCTNNNNAATAATATCCATCCTGCTGCTT	GACGTGTGCTCTTCCGATCTGGATTTTGCTATACTGGTCAGGC
5-15	chr10:28771420	ACACGACGCTCTTCCGATCTNNNNTTTGGCAGAGACGGGTTT	GACGTGTGCTCTTCCGATCTCCCGGCAAGACTGAGCAC
5-16	chr18:72313260	ACACGACGCTCTTCCGATCTNNNNTCCTCAAAGAACCTGAAAG	GACGTGTGCTCTTCCGATCTGTCCTGTCTACCACTGAT
5-17	chr6:48877872	ACACGACGCTCTTCCGATCTNNNNGAGAATATAAAACAGAGACCCAGGC	GACGTGTGCTCTTCCGATCTCACCACCATGCCCGGATA
5-18	chr14:22550940	ACACGACGCTCTTCCGATCTNNNNATGGCCTGAAACGCTGTAAAATA	GACGTGTGCTCTTCCGATCTCACAAGTGTCTCTCTTCCTCC
5-19	chr1:93333546	ACACGACGCTCTTCCGATCTNNNNGACTCCGTCTAAGAAAACAAAAC	GACGTGTGCTCTTCCGATCTAAATTCCTTCAACCAAACATCAA
5-20	chr4:78345702	ACACGACGCTCTTCCGATCTNNNNATTTCTCATCTTGTCACCCTA	GACGTGTGCTCTTCCGATCTCCCCTTTGCAGAGAGATTGA
5-21	chr5:180298360	ACACGACGCTCTTCCGATCTNNNNGCTGCTCTGTACAAACCAGGA	GACGTGTGCTCTTCCGATCTTTGAGAAAAGGTGCTTGAGA
5-22	chr9:122350718	ACACGACGCTCTTCCGATCTNNNNGTTTCCGTGGTCTCGTGGCAAA	GACGTGTGCTCTTCCGATCTCTCCCCTGCCTGTGAGTG
5-23	chr9:128878078	ACACGACGCTCTTCCGATCTNNNNCACCATAAACTAGCTATGGGACC	GACGTGTGCTCTTCCGATCTTCATTGACAAGTGGCTGTGAG
5-24	chr10:59689980	ACACGACGCTCTTCCGATCTNNNNATTATCCTCCCTTCTTCATATCTT	GACGTGTGCTCTTCCGATCTATCTTGCTGCATTTCTATAAACA
5-25	chr11:94681368	ACACGACGCTCTTCCGATCTNNNNACTTGTCAGTCTCTCTCACT	GACGTGTGCTCTTCCGATCTCATTTAATGAGACATGTTGACA
5-26	chr16:74830392	ACACGACGCTCTTCCGATCTNNNNACCTGGCTAATTTTGATAGAGATGG	GACGTGTGCTCTTCCGATCTCATCAGACCCGAGCTCAGTG
5-27	chrX:114136706	ACACGACGCTCTTCCGATCTNNNNCGCCCACCACCACGCTTG	GACGTGTGCTCTTCCGATCTTGGCCTTCTAAAATTTGAAATTCGG

Supplementary Table 6 | Primers used for deep sequencing experiments performed in this study. Primers are listed by figure and sample name. In some cases all samples in the figure use the same amplicon. These are indicated in the "Sample" column in the table. Primer sequences are 5' to 3'. The "N" bases in the forward primers are used to allow for cluster identification. At the bottom of the table are the primers used to add the MiSeq adapter as well as the plate and sample barcodes.

Sample	Forward Primer	Reverse Primer
**Fig. 3**		
NC-CN-6-11-N6a	ACACGACGCTCTTCCGATCTNNNNCTCTGGCCCACTGTTTCCC	GACGTGTGCTCTTCCGATCTAGGGAGTGGAGGAAGACGGA
NC-CN-6-11-N6b	ACACGACGCTCTTCCGATCTNNNNCTCTGGCCCACTGTTTCCC	GACGTGTGCTCTTCCGATCTAGGGAGTGGAGGAAGACGGA
NC-CN-6-11-N6c	ACACGACGCTCTTCCGATCTNNNNCTCTGGCCCACTGTTTCCC	GACGTGTGCTCTTCCGATCTAGGGAGTGGAGGAAGACGGA
NC-CN-6-12-N6a	ACACGACGCTCTTCCGATCTNNNNCTCTGGCCCACTGTTTCCC	GACGTGTGCTCTTCCGATCTAGGGAGTGGAGGAAGACGGA
NC-CN-6-12-N6b	ACACGACGCTCTTCCGATCTNNNNCTCTGGCCCACTGTTTCCC	GACGTGTGCTCTTCCGATCTAGGGAGTGGAGGAAGACGGA
NC-CN-6-12-N6c	ACACGACGCTCTTCCGATCTNNNNCTCTGGCCCACTGTTTCCC	GACGTGTGCTCTTCCGATCTAGGGAGTGGAGGAAGACGGA
NC-CN-6-13-N6a	ACACGACGCTCTTCCGATCTNNNNCGTGTCTGGGTCCTCTCCG	GACGTGTGCTCTTCCGATCTACGGTGATGATGCAGGCCT
NC-CN-6-13-N6b	ACACGACGCTCTTCCGATCTNNNNCGTGTCTGGGTCCTCTCCG	GACGTGTGCTCTTCCGATCTACGGTGATGATGCAGGCCT
NC-CN-6-13-N6c	ACACGACGCTCTTCCGATCTNNNNCGTGTCTGGGTCCTCTCCG	GACGTGTGCTCTTCCGATCTACGGTGATGATGCAGGCCT
NC-CN-6-14-N6a	ACACGACGCTCTTCCGATCTNNNNCGTGTCTGGGTCCTCTCCG	GACGTGTGCTCTTCCGATCTACGGTGATGATGCAGGCCT
NC-CN-6-14-N6b	ACACGACGCTCTTCCGATCTNNNNCGTGTCTGGGTCCTCTCCG	GACGTGTGCTCTTCCGATCTACGGTGATGATGCAGGCCT
NC-CN-6-14-N6c	ACACGACGCTCTTCCGATCTNNNNCGTGTCTGGGTCCTCTCCG	GACGTGTGCTCTTCCGATCTACGGTGATGATGCAGGCCT
NC-CN-6-15-N6a	ACACGACGCTCTTCCGATCTNNNNAGGCCTGCATCATCACCGT	GACGTGTGCTCTTCCGATCTAGCTGCCCAAATGAAAGGAGTG
NC-CN-6-15-N6b	ACACGACGCTCTTCCGATCTNNNNAGGCCTGCATCATCACCGT	GACGTGTGCTCTTCCGATCTAGCTGCCCAAATGAAAGGAGTG
NC-CN-6-15-N6c	ACACGACGCTCTTCCGATCTNNNNAGGCCTGCATCATCACCGT	GACGTGTGCTCTTCCGATCTAGCTGCCCAAATGAAAGGAGTG
NC-CN-6-16-N6a	ACACGACGCTCTTCCGATCTNNNNTCCTTTCATTTGGGCAGCTCC	GACGTGTGCTCTTCCGATCTCCCTGGAGGCAGCAAACA
NC-CN-6-16-N6b	ACACGACGCTCTTCCGATCTNNNNTCCTTTCATTTGGGCAGCTCC	GACGTGTGCTCTTCCGATCTCCCTGGAGGCAGCAAACA
NC-CN-6-16-N6c	ACACGACGCTCTTCCGATCTNNNNTCCTTTCATTTGGGCAGCTCC	GACGTGTGCTCTTCCGATCTCCCTGGAGGCAGCAAACA
NC-CN-6-17-N6a	ACACGACGCTCTTCCGATCTNNNNGCCTCCTCCTTCCTAGTCTCCT	GACGTGTGCTCTTCCGATCTGCCAAGCTCTCCCTCCCAG
NC-CN-6-17-N6b	ACACGACGCTCTTCCGATCTNNNNGCCTCCTCCTTCCTAGTCTCCT	GACGTGTGCTCTTCCGATCTGCCAAGCTCTCCCTCCCAG
NC-CN-6-17-N6c	ACACGACGCTCTTCCGATCTNNNNGCCTCCTCCTTCCTAGTCTCCT	GACGTGTGCTCTTCCGATCTGCCAAGCTCTCCCTCCCAG
NC-CN-6-18-N6a	ACACGACGCTCTTCCGATCTNNNNCTGGGAGGGAGAGCTTGGC	GACGTGTGCTCTTCCGATCTCTGCAGCACCAGGATCAGTG
NC-CN-6-18-N6b	ACACGACGCTCTTCCGATCTNNNNCTGGGAGGGAGAGCTTGGC	GACGTGTGCTCTTCCGATCTCTGCAGCACCAGGATCAGTG
NC-CN-6-18-N6c	ACACGACGCTCTTCCGATCTNNNNCTGGGAGGGAGAGCTTGGC	GACGTGTGCTCTTCCGATCTCTGCAGCACCAGGATCAGTG
NC-CN-6-19-N6a	ACACGACGCTCTTCCGATCTNNNNACCTGTGAGATAAGGCCAGT	GACGTGTGCTCTTCCGATCTGGATGGAGAAAGAGAAAGGGAGT
NC-CN-6-19-N6b	ACACGACGCTCTTCCGATCTNNNNACCTGTGAGATAAGGCCAGT	GACGTGTGCTCTTCCGATCTGGATGGAGAAAGAGAAAGGGAGT
NC-CN-6-19-N6c	ACACGACGCTCTTCCGATCTNNNNACCTGTGAGATAAGGCCAGT	GACGTGTGCTCTTCCGATCTGGATGGAGAAAGAGAAAGGGAGT
NC-CN-6-20-N6a	ACACGACGCTCTTCCGATCTNNNNCTGGGTTTGAGTCCTTGGCA	GACGTGTGCTCTTCCGATCTCAGGGGAACGGGGATGCAG
NC-CN-6-20-N6b	ACACGACGCTCTTCCGATCTNNNNCTGGGTTTGAGTCCTTGGCA	GACGTGTGCTCTTCCGATCTCAGGGGAACGGGGATGCAG
NC-CN-6-20-N6c	ACACGACGCTCTTCCGATCTNNNNCTGGGTTTGAGTCCTTGGCA	GACGTGTGCTCTTCCGATCTCAGGGGAACGGGGATGCAG
NC-CN-6-21-N6a	ACACGACGCTCTTCCGATCTNNNNCTGGGTTTGAGTCCTTGGCA	GACGTGTGCTCTTCCGATCTCAGGGGAACGGGGATGCAG
NC-CN-6-21-N6b	ACACGACGCTCTTCCGATCTNNNNCTGGGTTTGAGTCCTTGGCA	GACGTGTGCTCTTCCGATCTCAGGGGAACGGGGATGCAG
NC-CN-6-21-N6c	ACACGACGCTCTTCCGATCTNNNNCTGGGTTTGAGTCCTTGGCA	GACGTGTGCTCTTCCGATCTCAGGGGAACGGGGATGCAG
NC-CN-6-22-N6a	ACACGACGCTCTTCCGATCTNNNNTCGTCCACCATCTCATGCCC	GACGTGTGCTCTTCCGATCTTGGCCTCTCGTGGGGTCCA
NC-CN-6-22-N6b	ACACGACGCTCTTCCGATCTNNNNTCGTCCACCATCTCATGCCC	GACGTGTGCTCTTCCGATCTTGGCCTCTCGTGGGGTCCA
NC-CN-6-22-N6c	ACACGACGCTCTTCCGATCTNNNNTCGTCCACCATCTCATGCCC	GACGTGTGCTCTTCCGATCTTGGCCTCTCGTGGGGTCCA
NC-CN-6-23-N6a	ACACGACGCTCTTCCGATCTNNNNTGTCTACCAGGCTGCCTT	GACGTGTGCTCTTCCGATCTCCACACCCAGACCTGACCC
NC-CN-6-23-N6b	ACACGACGCTCTTCCGATCTNNNNTGTCTACCAGGCTGCCTT	GACGTGTGCTCTTCCGATCTCCACACCCAGACCTGACCC
NC-CN-6-23-N6c	ACACGACGCTCTTCCGATCTNNNNTGTCTACCAGGCTGCCTT	GACGTGTGCTCTTCCGATCTCCACACCCAGACCTGACCC
NC-CN-6-24-N6a	ACACGACGCTCTTCCGATCTNNNNCCCCTCCCCTATCGCCAT	GACGTGTGCTCTTCCGATCTCCCCAAGTCCCTCACCTCT
NC-CN-6-24-N6b	ACACGACGCTCTTCCGATCTNNNNCCCCTCCCCTATCGCCAT	GACGTGTGCTCTTCCGATCTCCCCAAGTCCCTCACCTCT
NC-CN-6-24-N6c	ACACGACGCTCTTCCGATCTNNNNCCCCTCCCCTATCGCCAT	GACGTGTGCTCTTCCGATCTCCCCAAGTCCCTCACCTCT
NC-CN-6-25-N6a	ACACGACGCTCTTCCGATCTNNNNCCCCTCCCCTATCGCCAT	GACGTGTGCTCTTCCGATCTCCCCAAGTCCCTCACCTCT
NC-CN-6-25-N6b	ACACGACGCTCTTCCGATCTNNNNCCCCTCCCCTATCGCCAT	GACGTGTGCTCTTCCGATCTCCCCAAGTCCCTCACCTCT
NC-CN-6-25-N6c	ACACGACGCTCTTCCGATCTNNNNCCCCTCCCCTATCGCCAT	GACGTGTGCTCTTCCGATCTCCCCAAGTCCCTCACCTCT
NC-CN-6-26-N6a	ACACGACGCTCTTCCGATCTNNNNCCCGTGGATCTAGGAGTTTAGC	GACGTGTGCTCTTCCGATCTCCGTTTCTCATTCTTCCCTTAGG
NC-CN-6-26-N6b	ACACGACGCTCTTCCGATCTNNNNCCCGTGGATCTAGGAGTTTAGC	GACGTGTGCTCTTCCGATCTCCGTTTCTCATTCTTCCCTTAGG
NC-CN-6-26-N6c	ACACGACGCTCTTCCGATCTNNNNCCCGTGGATCTAGGAGTTTAGC	GACGTGTGCTCTTCCGATCTCCGTTTCTCATTCTTCCCTTAGG
NC-CN-6-27-N6a	ACACGACGCTCTTCCGATCTNNNNGTGTGCAGCTGTCTCACCC	GACGTGTGCTCTTCCGATCTCTCCTGCCTTAAACCCAGCC
NC-CN-6-27-N6b	ACACGACGCTCTTCCGATCTNNNNGTGTGCAGCTGTCTCACCC	GACGTGTGCTCTTCCGATCTCTCCTGCCTTAAACCCAGCC
NC-CN-6-27-N6c	ACACGACGCTCTTCCGATCTNNNNGTGTGCAGCTGTCTCACCC	GACGTGTGCTCTTCCGATCTCTCCTGCCTTAAACCCAGCC
NC-CN-6-28-N6a	ACACGACGCTCTTCCGATCTNNNNGTGTGCAGCTGTCTCACCC	GACGTGTGCTCTTCCGATCTCTCCTGCCTTAAACCCAGCC
NC-CN-6-28-N6b	ACACGACGCTCTTCCGATCTNNNNGTGTGCAGCTGTCTCACCC	GACGTGTGCTCTTCCGATCTCTCCTGCCTTAAACCCAGCC
NC-CN-6-28-N6c	ACACGACGCTCTTCCGATCTNNNNGTGTGCAGCTGTCTCACCC	GACGTGTGCTCTTCCGATCTCTCCTGCCTTAAACCCAGCC
NC-CN-6-29-N6a	ACACGACGCTCTTCCGATCTNNNNGCTGGGTTTAAGGCAGGAG	GACGTGTGCTCTTCCGATCTTCTCATCTGGGTGCGGGA
NC-CN-6-29-N6b	ACACGACGCTCTTCCGATCTNNNNGCTGGGTTTAAGGCAGGAG	GACGTGTGCTCTTCCGATCTTCTCATCTGGGTGCGGGA
NC-CN-6-29-N6c	ACACGACGCTCTTCCGATCTNNNNGCTGGGTTTAAGGCAGGAG	GACGTGTGCTCTTCCGATCTTCTCATCTGGGTGCGGGA
NC-CN-7-11-N7a	ACACGACGCTCTTCCGATCTNNNNCTCTGGCCCACTGTTTCCC	GACGTGTGCTCTTCCGATCTAGGGAGTGGAGGAAGACGGA
NC-CN-7-11-N7b	ACACGACGCTCTTCCGATCTNNNNCTCTGGCCCACTGTTTCCC	GACGTGTGCTCTTCCGATCTAGGGAGTGGAGGAAGACGGA
NC-CN-7-11-N7c	ACACGACGCTCTTCCGATCTNNNNCTCTGGCCCACTGTTTCCC	GACGTGTGCTCTTCCGATCTAGGGAGTGGAGGAAGACGGA
NC-CN-7-12-N7a	ACACGACGCTCTTCCGATCTNNNNCTCTGGCCCACTGTTTCCC	GACGTGTGCTCTTCCGATCTAGGGAGTGGAGGAAGACGGA
NC-CN-7-12-N7b	ACACGACGCTCTTCCGATCTNNNNCTCTGGCCCACTGTTTCCC	GACGTGTGCTCTTCCGATCTAGGGAGTGGAGGAAGACGGA

NC-CN-7-12-N7c	ACACGACGCTCTTCCGATCTNNNNCTCTGGCCCACTGTTTCCC	GACGTGTGCTCTTCCGATCTAGGGAGTGGAGGAAGACGGA
NC-CN-7-13-N7a	ACACGACGCTCTTCCGATCTNNNNCGTGTCTGGGTCCTCTCCG	GACGTGTGCTCTTCCGATCTACGGTGATGATGCAGGCCT
NC-CN-7-13-N7b	ACACGACGCTCTTCCGATCTNNNNCGTGTCTGGGTCCTCTCCG	GACGTGTGCTCTTCCGATCTACGGTGATGATGCAGGCCT
NC-CN-7-13-N7c	ACACGACGCTCTTCCGATCTNNNNCGTGTCTGGGTCCTCTCCG	GACGTGTGCTCTTCCGATCTACGGTGATGATGCAGGCCT
NC-CN-7-14-N7a	ACACGACGCTCTTCCGATCTNNNNCGTGTCTGGGTCCTCTCCG	GACGTGTGCTCTTCCGATCTACGGTGATGATGCAGGCCT
NC-CN-7-14-N7b	ACACGACGCTCTTCCGATCTNNNNCGTGTCTGGGTCCTCTCCG	GACGTGTGCTCTTCCGATCTACGGTGATGATGCAGGCCT
NC-CN-7-14-N7c	ACACGACGCTCTTCCGATCTNNNNCGTGTCTGGGTCCTCTCCG	GACGTGTGCTCTTCCGATCTACGGTGATGATGCAGGCCT
NC-CN-7-15-N7a	ACACGACGCTCTTCCGATCTNNNNAGGCCTGCATCATCACCGT	GACGTGTGCTCTTCCGATCTAGCTGCCCAAATGAAAGGAGTG
NC-CN-7-15-N7b	ACACGACGCTCTTCCGATCTNNNNAGGCCTGCATCATCACCGT	GACGTGTGCTCTTCCGATCTAGCTGCCCAAATGAAAGGAGTG
NC-CN-7-15-N7c	ACACGACGCTCTTCCGATCTNNNNAGGCCTGCATCATCACCGT	GACGTGTGCTCTTCCGATCTAGCTGCCCAAATGAAAGGAGTG
NC-CN-7-16-N7a	ACACGACGCTCTTCCGATCTNNNNTCCCAGGGCCGGTTAATGT	GACGTGTGCTCTTCCGATCTGGTTAGACCCAATATCAGGAGAC
NC-CN-7-16-N7b	ACACGACGCTCTTCCGATCTNNNNTCCCAGGGCCGGTTAATGT	GACGTGTGCTCTTCCGATCTGGTTAGACCCAATATCAGGAGAC
NC-CN-7-16-N7c	ACACGACGCTCTTCCGATCTNNNNTCCCAGGGCCGGTTAATGT	GACGTGTGCTCTTCCGATCTGGTTAGACCCAATATCAGGAGAC
NC-CN-7-19-N7a	ACACGACGCTCTTCCGATCTNNNNACCTGTGAGATAAGGCCAGT	GACGTGTGCTCTTCCGATCTGGATGGAGAAAGAGAAAGGGAGT
NC-CN-7-19-N7b	ACACGACGCTCTTCCGATCTNNNNACCTGTGAGATAAGGCCAGT	GACGTGTGCTCTTCCGATCTGGATGGAGAAAGAGAAAGGGAGT
NC-CN-7-19-N7c	ACACGACGCTCTTCCGATCTNNNNACCTGTGAGATAAGGCCAGT	GACGTGTGCTCTTCCGATCTGGATGGAGAAAGAGAAAGGGAGT
NC-CN-7-20-N7a	ACACGACGCTCTTCCGATCTNNNNCTGGGTTTGAGTCCTTGGCA	GACGTGTGCTCTTCCGATCTCAGGGGAACGGGGATGCAG
NC-CN-7-20-N7b	ACACGACGCTCTTCCGATCTNNNNCTGGGTTTGAGTCCTTGGCA	GACGTGTGCTCTTCCGATCTCAGGGGAACGGGGATGCAG
NC-CN-7-20-N7c	ACACGACGCTCTTCCGATCTNNNNCTGGGTTTGAGTCCTTGGCA	GACGTGTGCTCTTCCGATCTCAGGGGAACGGGGATGCAG
NC-CN-7-21-N7a	ACACGACGCTCTTCCGATCTNNNNTCGTCCACCATCTCATGCCC	GACGTGTGCTCTTCCGATCTTGGCCTCTCGTGGGGTCCA
NC-CN-7-21-N7b	ACACGACGCTCTTCCGATCTNNNNTCGTCCACCATCTCATGCCC	GACGTGTGCTCTTCCGATCTTGGCCTCTCGTGGGGTCCA
NC-CN-7-21-N7c	ACACGACGCTCTTCCGATCTNNNNTCGTCCACCATCTCATGCCC	GACGTGTGCTCTTCCGATCTTGGCCTCTCGTGGGGTCCA
NC-CN-7-22-N7a	ACACGACGCTCTTCCGATCTNNNNTGTCTACCAGGCTGCCTT	GACGTGTGCTCTTCCGATCTCCACACCCAGACCTGACCC
NC-CN-7-22-N7b	ACACGACGCTCTTCCGATCTNNNNTGTCTACCAGGCTGCCTT	GACGTGTGCTCTTCCGATCTCCACACCCAGACCTGACCC
NC-CN-7-22-N7c	ACACGACGCTCTTCCGATCTNNNNTGTCTACCAGGCTGCCTT	GACGTGTGCTCTTCCGATCTCCACACCCAGACCTGACCC
NC-CN-7-23-N7a	ACACGACGCTCTTCCGATCTNNNNAGGTCCCCAAGCATCGCC	GACGTGTGCTCTTCCGATCTAGCCCCTCCTACTCTAGCCC
NC-CN-7-23-N7b	ACACGACGCTCTTCCGATCTNNNNAGGTCCCCAAGCATCGCC	GACGTGTGCTCTTCCGATCTAGCCCCTCCTACTCTAGCCC
NC-CN-7-23-N7c	ACACGACGCTCTTCCGATCTNNNNAGGTCCCCAAGCATCGCC	GACGTGTGCTCTTCCGATCTAGCCCCTCCTACTCTAGCCC
NC-CN-7-24-N7a	ACACGACGCTCTTCCGATCTNNNNCCCCTCCCCTATCGCCAT	GACGTGTGCTCTTCCGATCTCCCCAAGTCCCTCACCTCT
NC-CN-7-24-N7b	ACACGACGCTCTTCCGATCTNNNNCCCCTCCCCTATCGCCAT	GACGTGTGCTCTTCCGATCTCCCCAAGTCCCTCACCTCT
NC-CN-7-24-N7c	ACACGACGCTCTTCCGATCTNNNNCCCCTCCCCTATCGCCAT	GACGTGTGCTCTTCCGATCTCCCCAAGTCCCTCACCTCT
NC-CN-7-26-N7a	ACACGACGCTCTTCCGATCTNNNNGTGTGCAGCTGTCTCACCC	GACGTGTGCTCTTCCGATCTCTCCTGCCTTAAACCCAGCC
NC-CN-7-26-N7b	ACACGACGCTCTTCCGATCTNNNNGTGTGCAGCTGTCTCACCC	GACGTGTGCTCTTCCGATCTCTCCTGCCTTAAACCCAGCC
NC-CN-7-26-N7c	ACACGACGCTCTTCCGATCTNNNNGTGTGCAGCTGTCTCACCC	GACGTGTGCTCTTCCGATCTCTCCTGCCTTAAACCCAGCC
NC-CN-7-27-N7a	ACACGACGCTCTTCCGATCTNNNNGCTGGGTTTAAGGCAGGAG	GACGTGTGCTCTTCCGATCTTCTCATCTGGGTGCGGGA
NC-CN-7-27-N7b	ACACGACGCTCTTCCGATCTNNNNGCTGGGTTTAAGGCAGGAG	GACGTGTGCTCTTCCGATCTTCTCATCTGGGTGCGGGA
NC-CN-7-27-N7c	ACACGACGCTCTTCCGATCTNNNNGCTGGGTTTAAGGCAGGAG	GACGTGTGCTCTTCCGATCTTCTCATCTGGGTGCGGGA
NC-CN-7-28-N7a	ACACGACGCTCTTCCGATCTNNNNGCTGGGTTTAAGGCAGGAG	GACGTGTGCTCTTCCGATCTTCTCATCTGGGTGCGGGA
NC-CN-7-28-N7b	ACACGACGCTCTTCCGATCTNNNNGCTGGGTTTAAGGCAGGAG	GACGTGTGCTCTTCCGATCTTCTCATCTGGGTGCGGGA
NC-CN-7-28-N7c	ACACGACGCTCTTCCGATCTNNNNGCTGGGTTTAAGGCAGGAG	GACGTGTGCTCTTCCGATCTTCTCATCTGGGTGCGGGA
C11	ACACGACGCTCTTCCGATCTNNNNCTCTGGCCCACTGTTTCCC	GACGTGTGCTCTTCCGATCTAGGGAGTGGAGGAAGACGGA
C12	ACACGACGCTCTTCCGATCTNNNNCGTGTCTGGGTCCTCTCCG	GACGTGTGCTCTTCCGATCTACGGTGATGATGCAGGCCT
C13	ACACGACGCTCTTCCGATCTNNNNAGGCCTGCATCATCACCGT	GACGTGTGCTCTTCCGATCTAGCTGCCCAAATGAAAGGAGTG
C14	ACACGACGCTCTTCCGATCTNNNNTCCTTTCATTTGGGCAGCTCC	GACGTGTGCTCTTCCGATCTCCCTGGAGGCAGCAAACA
C15	ACACGACGCTCTTCCGATCTNNNNTCCTTTCATTTGGGCAGCTCC	GACGTGTGCTCTTCCGATCTCCCTGGAGGCAGCAAACA
C16	ACACGACGCTCTTCCGATCTNNNNTCCCAGGGCCGGTTAATGT	GACGTGTGCTCTTCCGATCTGGTTAGACCCAATATCAGGAGAC
C17	ACACGACGCTCTTCCGATCTNNNNGCCTCCTCCTTCCTAGTCTCCT	GACGTGTGCTCTTCCGATCTGCCAAGCTCTCCCTCCCAG
C18	ACACGACGCTCTTCCGATCTNNNNCTGGGAGGGAGAGCTTGGC	GACGTGTGCTCTTCCGATCTCTGCAGCACCAGGATCAGTG
C19	ACACGACGCTCTTCCGATCTNNNNACCTGTGAGATAAGGCCAGT	GACGTGTGCTCTTCCGATCTGGATGGAGAAAGAGAAAGGGAGT
C21	ACACGACGCTCTTCCGATCTNNNNTGTCTACCAGGCTGCCTT	GACGTGTGCTCTTCCGATCTCCACACCCAGACCTGACCC
C22	ACACGACGCTCTTCCGATCTNNNNTGTCTACCAGGCTGCCTT	GACGTGTGCTCTTCCGATCTCCACACCCAGACCTGACCC
C23	ACACGACGCTCTTCCGATCTNNNNAGGTCCCCAAGCATCGCC	GACGTGTGCTCTTCCGATCTAGCCCCTCCTACTCTAGCCC
C24	ACACGACGCTCTTCCGATCTNNNNGGTTGGGCCTGGACTCTG	GACGTGTGCTCTTCCGATCTGGGATGAACTCGGCTCGT
C25	ACACGACGCTCTTCCGATCTNNNNCCCCTCCCCTATCGCCAT	GACGTGTGCTCTTCCGATCTCCCCAAGTCCCTCACCTCT
C26	ACACGACGCTCTTCCGATCTNNNNCCCGTGGATCTAGGAGTTTAGC	GACGTGTGCTCTTCCGATCTCCGTTTCTCATTCTTCCCTTAGG
C27	ACACGACGCTCTTCCGATCTNNNNGTGTGCAGCTGTCTCACCC	GACGTGTGCTCTTCCGATCTCTCCTGCCTTAAACCCAGCC
C28	ACACGACGCTCTTCCGATCTNNNNGCTGGGTTTAAGGCAGGAG	GACGTGTGCTCTTCCGATCTTCTCATCTGGGTGCGGGA
C29	ACACGACGCTCTTCCGATCTNNNNAGGCGTATCTTAAACAGCCAGT	GACGTGTGCTCTTCCGATCTGTGGGAAACAGCCGTCAG

Fig. 4

HBG1 - all samples	ACACGACGCTCTTCCGATCTNNNNCTGGAATGACTGAATCGGAACAAG	GACGTGTGCTCTTCCGATCTGCCTCACTGGATACTCTAAGAC
CEP290	ACACGACGCTCTTCCGATCTNNNNGGATGGTGTCGATCTCCTGAAC	GACGTGTGCTCTTCCGATCTGAGACAGGAATAATGGCTGCCAC

Supplementary Fig. 5

AAVS1 locus - all samples	ACACGACGCTCTTCCGATCTNNNNGTGTGTCACCAGATAAGGAAT	GACGTGTGCTCTTCCGATCTGGCTCTGGTTCTGGGTACTTTTA

Supplementary Fig. 6

CCR5 locus - all samples	ACACGACGCTCTTCCGATCTNNNNGTGTTCATCTTTGGTTTTGTGG	GACGTGTGCTCTTCCGATCTCAGAAGGGGACAGTAAGAAGGA

Supplementary Fig. 7

NC/CN-6-1-L1	ACACGACGCTCTTCCGATCTNNNNCTTGGATTTCAGCGGCACAAG	GACGTGTGCTCTTCCGATCTAAAGTCTCACTCACCTTTGCAG
NC/CN-6-1-L2	ACACGACGCTCTTCCGATCTNNNNCTTGGATTTCAGCGGCACAAG	GACGTGTGCTCTTCCGATCTAAAGTCTCACTCACCTTTGCAG
NC/CN-6-1-L3	ACACGACGCTCTTCCGATCTNNNNCTTGGATTTCAGCGGCACAAG	GACGTGTGCTCTTCCGATCTAAAGTCTCACTCACCTTTGCAG
NC/CN-6-1-L4	ACACGACGCTCTTCCGATCTNNNNCTTGGATTTCAGCGGCACAAG	GACGTGTGCTCTTCCGATCTAAAGTCTCACTCACCTTTGCAG
NC/CN-6-1-L5	ACACGACGCTCTTCCGATCTNNNNCTTGGATTTCAGCGGCACAAG	GACGTGTGCTCTTCCGATCTAAAGTCTCACTCACCTTTGCAG
NC/CN-6-1-L6	ACACGACGCTCTTCCGATCTNNNNCTTGGATTTCAGCGGCACAAG	GACGTGTGCTCTTCCGATCTAAAGTCTCACTCACCTTTGCAG
NC/CN-6-1-L7	ACACGACGCTCTTCCGATCTNNNNCTTGGATTTCAGCGGCACAAG	GACGTGTGCTCTTCCGATCTAAAGTCTCACTCACCTTTGCAG
NC/CN-6-1-L8	ACACGACGCTCTTCCGATCTNNNNCTTGGATTTCAGCGGCACAAG	GACGTGTGCTCTTCCGATCTAAAGTCTCACTCACCTTTGCAG
NC/CN-6-2-L1	ACACGACGCTCTTCCGATCTNNNNCTTGGATTTCAGCGGCACAAG	GACGTGTGCTCTTCCGATCTAAGTCTCACTCACCTTTGCAGA
NC/CN-6-2-L2	ACACGACGCTCTTCCGATCTNNNNCTTGGATTTCAGCGGCACAAG	GACGTGTGCTCTTCCGATCTAAGTCTCACTCACCTTTGCAGA
NC/CN-6-2-L3	ACACGACGCTCTTCCGATCTNNNNCTTGGATTTCAGCGGCACAAG	GACGTGTGCTCTTCCGATCTAAGTCTCACTCACCTTTGCAGA
NC/CN-6-2-L4	ACACGACGCTCTTCCGATCTNNNNCTTGGATTTCAGCGGCACAAG	GACGTGTGCTCTTCCGATCTAAGTCTCACTCACCTTTGCAGA
NC/CN-6-2-L5	ACACGACGCTCTTCCGATCTNNNNCTTGGATTTCAGCGGCACAAG	GACGTGTGCTCTTCCGATCTAAGTCTCACTCACCTTTGCAGA
NC/CN-6-2-L6	ACACGACGCTCTTCCGATCTNNNNCTTGGATTTCAGCGGCACAAG	GACGTGTGCTCTTCCGATCTAAGTCTCACTCACCTTTGCAGA
NC/CN-6-2-L7	ACACGACGCTCTTCCGATCTNNNNCTTGGATTTCAGCGGCACAAG	GACGTGTGCTCTTCCGATCTAAGTCTCACTCACCTTTGCAGA
NC/CN-6-2-L8	ACACGACGCTCTTCCGATCTNNNNCTTGGATTTCAGCGGCACAAG	GACGTGTGCTCTTCCGATCTAAGTCTCACTCACCTTTGCAGA
NC/CN-6-3-L1	ACACGACGCTCTTCCGATCTNNNNAGGAGCATGAGTTCACTGAGTT	GACGTGTGCTCTTCCGATCTCTTTGCCTGGAGATGCATACTC
NC/CN-6-3-L2	ACACGACGCTCTTCCGATCTNNNNAGGAGCATGAGTTCACTGAGTT	GACGTGTGCTCTTCCGATCTCTTTGCCTGGAGATGCATACTC
NC/CN-6-3-L3	ACACGACGCTCTTCCGATCTNNNNAGGAGCATGAGTTCACTGAGTT	GACGTGTGCTCTTCCGATCTCTTTGCCTGGAGATGCATACTC
NC/CN-6-3-L4	ACACGACGCTCTTCCGATCTNNNNAGGAGCATGAGTTCACTGAGTT	GACGTGTGCTCTTCCGATCTCTTTGCCTGGAGATGCATACTC
NC/CN-6-3-L5	ACACGACGCTCTTCCGATCTNNNNAGGAGCATGAGTTCACTGAGTT	GACGTGTGCTCTTCCGATCTCTTTGCCTGGAGATGCATACTC
NC/CN-6-3-L6	ACACGACGCTCTTCCGATCTNNNNAGGAGCATGAGTTCACTGAGTT	GACGTGTGCTCTTCCGATCTCTTTGCCTGGAGATGCATACTC
NC/CN-6-3-L7	ACACGACGCTCTTCCGATCTNNNNAGGAGCATGAGTTCACTGAGTT	GACGTGTGCTCTTCCGATCTCTTTGCCTGGAGATGCATACTC
NC/CN-6-3-L8	ACACGACGCTCTTCCGATCTNNNNAGGAGCATGAGTTCACTGAGTT	GACGTGTGCTCTTCCGATCTCTTTGCCTGGAGATGCATACTC
NC/CN-6-4-L1	ACACGACGCTCTTCCGATCTNNNNCCTGCTGTGGTACTGGCC	GACGTGTGCTCTTCCGATCTTAGGTTGCCGCACAGACTTCAG
NC/CN-6-4-L2	ACACGACGCTCTTCCGATCTNNNNCCTGCTGTGGTACTGGCC	GACGTGTGCTCTTCCGATCTTAGGTTGCCGCACAGACTTCAG
NC/CN-6-4-L3	ACACGACGCTCTTCCGATCTNNNNCCTGCTGTGGTACTGGCC	GACGTGTGCTCTTCCGATCTTAGGTTGCCGCACAGACTTCAG
NC/CN-6-4-L4	ACACGACGCTCTTCCGATCTNNNNCCTGCTGTGGTACTGGCC	GACGTGTGCTCTTCCGATCTTAGGTTGCCGCACAGACTTCAG
NC/CN-6-4-L5	ACACGACGCTCTTCCGATCTNNNNCCTGCTGTGGTACTGGCC	GACGTGTGCTCTTCCGATCTTAGGTTGCCGCACAGACTTCAG
NC/CN-6-4-L6	ACACGACGCTCTTCCGATCTNNNNCCTGCTGTGGTACTGGCC	GACGTGTGCTCTTCCGATCTTAGGTTGCCGCACAGACTTCAG
NC/CN-6-4-L7	ACACGACGCTCTTCCGATCTNNNNCCTGCTGTGGTACTGGCC	GACGTGTGCTCTTCCGATCTTAGGTTGCCGCACAGACTTCAG
NC/CN-6-4-L8	ACACGACGCTCTTCCGATCTNNNNCCTGCTGTGGTACTGGCC	GACGTGTGCTCTTCCGATCTTAGGTTGCCGCACAGACTTCAG
NC/CN-6-5-L1	ACACGACGCTCTTCCGATCTNNNNGTGTGTGAGTATGCATCTCCAG	GACGTGTGCTCTTCCGATCTAGGTCAACTCATTCCCCATCAT
NC/CN-6-5-L2	ACACGACGCTCTTCCGATCTNNNNGTGTGTGAGTATGCATCTCCAG	GACGTGTGCTCTTCCGATCTAGGTCAACTCATTCCCCATCAT
NC/CN-6-5-L3	ACACGACGCTCTTCCGATCTNNNNGTGTGTGAGTATGCATCTCCAG	GACGTGTGCTCTTCCGATCTAGGTCAACTCATTCCCCATCAT
NC/CN-6-5-L4	ACACGACGCTCTTCCGATCTNNNNGTGTGTGAGTATGCATCTCCAG	GACGTGTGCTCTTCCGATCTAGGTCAACTCATTCCCCATCAT
NC/CN-6-5-L5	ACACGACGCTCTTCCGATCTNNNNGTGTGTGAGTATGCATCTCCAG	GACGTGTGCTCTTCCGATCTAGGTCAACTCATTCCCCATCAT
NC/CN-6-5-L6	ACACGACGCTCTTCCGATCTNNNNGTGTGTGAGTATGCATCTCCAG	GACGTGTGCTCTTCCGATCTAGGTCAACTCATTCCCCATCAT
NC/CN-6-5-L7	ACACGACGCTCTTCCGATCTNNNNGTGTGTGAGTATGCATCTCCAG	GACGTGTGCTCTTCCGATCTAGGTCAACTCATTCCCCATCAT
NC/CN-6-5-L8	ACACGACGCTCTTCCGATCTNNNNGTGTGTGAGTATGCATCTCCAG	GACGTGTGCTCTTCCGATCTAGGTCAACTCATTCCCCATCAT
NC/CN-6-6-L1	ACACGACGCTCTTCCGATCTNNNNTCCGGGTGACAGTGCTTC	GACGTGTGCTCTTCCGATCTAGGTTCACTTGATTTCCACTGGA
NC/CN-6-6-L2	ACACGACGCTCTTCCGATCTNNNNTCCGGGTGACAGTGCTTC	GACGTGTGCTCTTCCGATCTAGGTTCACTTGATTTCCACTGGA
NC/CN-6-6-L3	ACACGACGCTCTTCCGATCTNNNNTCCGGGTGACAGTGCTTC	GACGTGTGCTCTTCCGATCTAGGTTCACTTGATTTCCACTGGA
NC/CN-6-6-L4	ACACGACGCTCTTCCGATCTNNNNTCCGGGTGACAGTGCTTC	GACGTGTGCTCTTCCGATCTAGGTTCACTTGATTTCCACTGGA
NC/CN-6-6-L5	ACACGACGCTCTTCCGATCTNNNNTCCGGGTGACAGTGCTTC	GACGTGTGCTCTTCCGATCTAGGTTCACTTGATTTCCACTGGA
NC/CN-6-6-L6	ACACGACGCTCTTCCGATCTNNNNTCCGGGTGACAGTGCTTC	GACGTGTGCTCTTCCGATCTAGGTTCACTTGATTTCCACTGGA
NC/CN-6-6-L7	ACACGACGCTCTTCCGATCTNNNNTCCGGGTGACAGTGCTTC	GACGTGTGCTCTTCCGATCTAGGTTCACTTGATTTCCACTGGA
NC/CN-6-6-L8	ACACGACGCTCTTCCGATCTNNNNTCCGGGTGACAGTGCTTC	GACGTGTGCTCTTCCGATCTAGGTTCACTTGATTTCCACTGGA
NC/CN-6-7-L1	ACACGACGCTCTTCCGATCTNNNNGATGGGGAATGAGTTGACCTTC	GACGTGTGCTCTTCCGATCTCTCCACCTTGCAGATGTAGAGT
NC/CN-6-7-L2	ACACGACGCTCTTCCGATCTNNNNGATGGGGAATGAGTTGACCTTC	GACGTGTGCTCTTCCGATCTCTCCACCTTGCAGATGTAGAGT
NC/CN-6-7-L3	ACACGACGCTCTTCCGATCTNNNNGATGGGGAATGAGTTGACCTTC	GACGTGTGCTCTTCCGATCTCTCCACCTTGCAGATGTAGAGT
NC/CN-6-7-L4	ACACGACGCTCTTCCGATCTNNNNGATGGGGAATGAGTTGACCTTC	GACGTGTGCTCTTCCGATCTCTCCACCTTGCAGATGTAGAGT
NC/CN-6-7-L5	ACACGACGCTCTTCCGATCTNNNNGATGGGGAATGAGTTGACCTTC	GACGTGTGCTCTTCCGATCTCTCCACCTTGCAGATGTAGAGT
NC/CN-6-7-L6	ACACGACGCTCTTCCGATCTNNNNGATGGGGAATGAGTTGACCTTC	GACGTGTGCTCTTCCGATCTCTCCACCTTGCAGATGTAGAGT
NC/CN-6-7-L7	ACACGACGCTCTTCCGATCTNNNNGATGGGGAATGAGTTGACCTTC	GACGTGTGCTCTTCCGATCTCTCCACCTTGCAGATGTAGAGT
NC/CN-6-7-L8	ACACGACGCTCTTCCGATCTNNNNGATGGGGAATGAGTTGACCTTC	GACGTGTGCTCTTCCGATCTCTCCACCTTGCAGATGTAGAGT
NC/CN-6-8-L1	ACACGACGCTCTTCCGATCTNNNNCATGGACACGGGACTCTACATC	GACGTGTGCTCTTCCGATCTCAACAGGTGTCAACTCAGTGAA
NC/CN-6-8-L2	ACACGACGCTCTTCCGATCTNNNNCATGGACACGGGACTCTACATC	GACGTGTGCTCTTCCGATCTCAACAGGTGTCAACTCAGTGAA
NC/CN-6-8-L3	ACACGACGCTCTTCCGATCTNNNNCATGGACACGGGACTCTACATC	GACGTGTGCTCTTCCGATCTCAACAGGTGTCAACTCAGTGAA
NC/CN-6-8-L4	ACACGACGCTCTTCCGATCTNNNNCATGGACACGGGACTCTACATC	GACGTGTGCTCTTCCGATCTCAACAGGTGTCAACTCAGTGAA
NC/CN-6-8-L5	ACACGACGCTCTTCCGATCTNNNNCATGGACACGGGACTCTACATC	GACGTGTGCTCTTCCGATCTCAACAGGTGTCAACTCAGTGAA
NC/CN-6-8-L6	ACACGACGCTCTTCCGATCTNNNNCATGGACACGGGACTCTACATC	GACGTGTGCTCTTCCGATCTCAACAGGTGTCAACTCAGTGAA
NC/CN-6-8-L7	ACACGACGCTCTTCCGATCTNNNNCATGGACACGGGACTCTACATC	GACGTGTGCTCTTCCGATCTCAACAGGTGTCAACTCAGTGAA
NC/CN-6-8-L8	ACACGACGCTCTTCCGATCTNNNNCATGGACACGGGACTCTACATC	GACGTGTGCTCTTCCGATCTCAACAGGTGTCAACTCAGTGAA
NC/CN-6-9-L1	ACACGACGCTCTTCCGATCTNNNNGACTCTACATCTGCAAGGTGGA	GACGTGTGCTCTTCCGATCTCAATGCAACAGGTGTCAACTCA
NC/CN-6-9-L2	ACACGACGCTCTTCCGATCTNNNNGACTCTACATCTGCAAGGTGGA	GACGTGTGCTCTTCCGATCTCAATGCAACAGGTGTCAACTCA
NC/CN-6-9-L3	ACACGACGCTCTTCCGATCTNNNNGACTCTACATCTGCAAGGTGGA	GACGTGTGCTCTTCCGATCTCAATGCAACAGGTGTCAACTCA
NC/CN-6-9-L4	ACACGACGCTCTTCCGATCTNNNNGACTCTACATCTGCAAGGTGGA	GACGTGTGCTCTTCCGATCTCAATGCAACAGGTGTCAACTCA
NC/CN-6-9-L5	ACACGACGCTCTTCCGATCTNNNNGACTCTACATCTGCAAGGTGGA	GACGTGTGCTCTTCCGATCTCAATGCAACAGGTGTCAACTCA
NC/CN-6-9-L6	ACACGACGCTCTTCCGATCTNNNNGACTCTACATCTGCAAGGTGGA	GACGTGTGCTCTTCCGATCTCAATGCAACAGGTGTCAACTCA
NC/CN-6-9-L7	ACACGACGCTCTTCCGATCTNNNNGACTCTACATCTGCAAGGTGGA	GACGTGTGCTCTTCCGATCTCAATGCAACAGGTGTCAACTCA
NC/CN-6-9-L8	ACACGACGCTCTTCCGATCTNNNNGACTCTACATCTGCAAGGTGGA	GACGTGTGCTCTTCCGATCTCAATGCAACAGGTGTCAACTCA
NC/CN-6-10-L1	ACACGACGCTCTTCCGATCTNNNNCATGGACACGGGACTCTACATC	GACGTGTGCTCTTCCGATCTAATGCAACAGGTGTCAACTCAG
NC/CN-6-10-L2	ACACGACGCTCTTCCGATCTNNNNCATGGACACGGGACTCTACATC	GACGTGTGCTCTTCCGATCTAATGCAACAGGTGTCAACTCAG
NC/CN-6-10-L3	ACACGACGCTCTTCCGATCTNNNNCATGGACACGGGACTCTACATC	GACGTGTGCTCTTCCGATCTAATGCAACAGGTGTCAACTCAG
NC/CN-6-10-L4	ACACGACGCTCTTCCGATCTNNNNCATGGACACGGGACTCTACATC	GACGTGTGCTCTTCCGATCTAATGCAACAGGTGTCAACTCAG
NC/CN-6-10-L5	ACACGACGCTCTTCCGATCTNNNNCATGGACACGGGACTCTACATC	GACGTGTGCTCTTCCGATCTAATGCAACAGGTGTCAACTCAG
NC/CN-6-10-L6	ACACGACGCTCTTCCGATCTNNNNCATGGACACGGGACTCTACATC	GACGTGTGCTCTTCCGATCTAATGCAACAGGTGTCAACTCAG
NC/CN-6-10-L7	ACACGACGCTCTTCCGATCTNNNNCATGGACACGGGACTCTACATC	GACGTGTGCTCTTCCGATCTAATGCAACAGGTGTCAACTCAG
NC/CN-6-10-L8	ACACGACGCTCTTCCGATCTNNNNCATGGACACGGGACTCTACATC	GACGTGTGCTCTTCCGATCTAATGCAACAGGTGTCAACTCAG

C1	ACACGACGCTCTTCCGATCTNNNNGCCCTGCACTCTCCTGTTTT	GACGTGTGCTCTTCCGATCTAAATCACTGCCTTTGACTGCTG
C2	ACACGACGCTCTTCCGATCTNNNNATGAAGGAGCATGAGTTCACTG	GACGTGTGCTCTTCCGATCTCTTTGCCTGGAGATGCATACTC
C3	ACACGACGCTCTTCCGATCTNNNNCCTGCTGTGGTACTGGCC	GACGTGTGCTCTTCCGATCTTAGGTTGCCGCACAGACTTCAG
C4	ACACGACGCTCTTCCGATCTNNNNCATCGCCAGCTTTGTGTGTG	GACGTGTGCTCTTCCGATCTGGTCAACTCATTCCCCATCATG
C5	ACACGACGCTCTTCCGATCTNNNNTCCGGGTGACAGTGCTTC	GACGTGTGCTCTTCCGATCTAGGTTCACTTGATTTCCACTGGA
C6	ACACGACGCTCTTCCGATCTNNNNACCTACATGATGGGGAATGAGT	GACGTGTGCTCTTCCGATCTCTCCACCTTGCAGATGTAGAGT
C7	ACACGACGCTCTTCCGATCTNNNNCCTCCAGTGGAAATCAAGTGAA	GACGTGTGCTCTTCCGATCTTAAATCTGGGTTCCGTTGCCTA
C8	ACACGACGCTCTTCCGATCTNNNNCATGGACACGGGACTCTACATC	GACGTGTGCTCTTCCGATCTGTGTCAACTCAGTGAAATGGCT
C9	ACACGACGCTCTTCCGATCTNNNNCATGGACACGGGACTCTACATC	GACGTGTGCTCTTCCGATCTGTGTCAACTCAGTGAAATGGCT
C10	ACACGACGCTCTTCCGATCTNNNNCATGGACACGGGACTCTACATC	GACGTGTGCTCTTCCGATCTAATGCAACAGGTGTCAACTCAG
NC/CN-7-1-L1	ACACGACGCTCTTCCGATCTNNNNCTTGGATTTCAGCGGCACAAG	GACGTGTGCTCTTCCGATCTAAAGTCTCACTCACCTTTGCAG
NC/CN-7-1-L2	ACACGACGCTCTTCCGATCTNNNNCTTGGATTTCAGCGGCACAAG	GACGTGTGCTCTTCCGATCTAAAGTCTCACTCACCTTTGCAG
NC/CN-7-1-L3	ACACGACGCTCTTCCGATCTNNNNCTTGGATTTCAGCGGCACAAG	GACGTGTGCTCTTCCGATCTAAAGTCTCACTCACCTTTGCAG
NC/CN-7-1-L4	ACACGACGCTCTTCCGATCTNNNNCTTGGATTTCAGCGGCACAAG	GACGTGTGCTCTTCCGATCTAAAGTCTCACTCACCTTTGCAG
NC/CN-7-1-L5	ACACGACGCTCTTCCGATCTNNNNCTTGGATTTCAGCGGCACAAG	GACGTGTGCTCTTCCGATCTAAAGTCTCACTCACCTTTGCAG
NC/CN-7-1-L6	ACACGACGCTCTTCCGATCTNNNNCTTGGATTTCAGCGGCACAAG	GACGTGTGCTCTTCCGATCTAAAGTCTCACTCACCTTTGCAG
NC/CN-7-1-L7	ACACGACGCTCTTCCGATCTNNNNCTTGGATTTCAGCGGCACAAG	GACGTGTGCTCTTCCGATCTAAAGTCTCACTCACCTTTGCAG
NC/CN-7-1-L8	ACACGACGCTCTTCCGATCTNNNNCTTGGATTTCAGCGGCACAAG	GACGTGTGCTCTTCCGATCTAAAGTCTCACTCACCTTTGCAG
NC/CN-7-2-L1	ACACGACGCTCTTCCGATCTNNNNCTTGGATTTCAGCGGCACAAG	GACGTGTGCTCTTCCGATCTCTCCAAAAGTCTCACTCACCTT
NC/CN-7-2-L2	ACACGACGCTCTTCCGATCTNNNNCTTGGATTTCAGCGGCACAAG	GACGTGTGCTCTTCCGATCTCTCCAAAAGTCTCACTCACCTT
NC/CN-7-2-L3	ACACGACGCTCTTCCGATCTNNNNCTTGGATTTCAGCGGCACAAG	GACGTGTGCTCTTCCGATCTCTCCAAAAGTCTCACTCACCTT
NC/CN-7-2-L4	ACACGACGCTCTTCCGATCTNNNNCTTGGATTTCAGCGGCACAAG	GACGTGTGCTCTTCCGATCTCTCCAAAAGTCTCACTCACCTT
NC/CN-7-2-L5	ACACGACGCTCTTCCGATCTNNNNCTTGGATTTCAGCGGCACAAG	GACGTGTGCTCTTCCGATCTCTCCAAAAGTCTCACTCACCTT
NC/CN-7-2-L6	ACACGACGCTCTTCCGATCTNNNNCTTGGATTTCAGCGGCACAAG	GACGTGTGCTCTTCCGATCTCTCCAAAAGTCTCACTCACCTT
NC/CN-7-2-L7	ACACGACGCTCTTCCGATCTNNNNCTTGGATTTCAGCGGCACAAG	GACGTGTGCTCTTCCGATCTCTCCAAAAGTCTCACTCACCTT
NC/CN-7-2-L8	ACACGACGCTCTTCCGATCTNNNNCTTGGATTTCAGCGGCACAAG	GACGTGTGCTCTTCCGATCTCTCCAAAAGTCTCACTCACCTT
NC/CN-7-3-L1	ACACGACGCTCTTCCGATCTNNNNAAGGAGCATGAGTTCACTGAGT	GACGTGTGCTCTTCCGATCTCTTTGCCTGGAGATGCATACTC
NC/CN-7-3-L2	ACACGACGCTCTTCCGATCTNNNNAAGGAGCATGAGTTCACTGAGT	GACGTGTGCTCTTCCGATCTCTTTGCCTGGAGATGCATACTC
NC/CN-7-3-L3	ACACGACGCTCTTCCGATCTNNNNAAGGAGCATGAGTTCACTGAGT	GACGTGTGCTCTTCCGATCTCTTTGCCTGGAGATGCATACTC
NC/CN-7-3-L4	ACACGACGCTCTTCCGATCTNNNNAAGGAGCATGAGTTCACTGAGT	GACGTGTGCTCTTCCGATCTCTTTGCCTGGAGATGCATACTC
NC/CN-7-3-L5	ACACGACGCTCTTCCGATCTNNNNAAGGAGCATGAGTTCACTGAGT	GACGTGTGCTCTTCCGATCTCTTTGCCTGGAGATGCATACTC
NC/CN-7-3-L6	ACACGACGCTCTTCCGATCTNNNNAAGGAGCATGAGTTCACTGAGT	GACGTGTGCTCTTCCGATCTCTTTGCCTGGAGATGCATACTC
NC/CN-7-3-L7	ACACGACGCTCTTCCGATCTNNNNAAGGAGCATGAGTTCACTGAGT	GACGTGTGCTCTTCCGATCTCTTTGCCTGGAGATGCATACTC
NC/CN-7-3-L8	ACACGACGCTCTTCCGATCTNNNNAAGGAGCATGAGTTCACTGAGT	GACGTGTGCTCTTCCGATCTCTTTGCCTGGAGATGCATACTC
NC/CN-7-4-L1	ACACGACGCTCTTCCGATCTNNNNCGCCAGCTTTGTGTGTGAGTA	GACGTGTGCTCTTCCGATCTAGGTCAACTCATTCCCCATCAT
NC/CN-7-4-L2	ACACGACGCTCTTCCGATCTNNNNCGCCAGCTTTGTGTGTGAGTA	GACGTGTGCTCTTCCGATCTAGGTCAACTCATTCCCCATCAT
NC/CN-7-4-L3	ACACGACGCTCTTCCGATCTNNNNCGCCAGCTTTGTGTGTGAGTA	GACGTGTGCTCTTCCGATCTAGGTCAACTCATTCCCCATCAT
NC/CN-7-4-L4	ACACGACGCTCTTCCGATCTNNNNCGCCAGCTTTGTGTGTGAGTA	GACGTGTGCTCTTCCGATCTAGGTCAACTCATTCCCCATCAT
NC/CN-7-4-L5	ACACGACGCTCTTCCGATCTNNNNCGCCAGCTTTGTGTGTGAGTA	GACGTGTGCTCTTCCGATCTAGGTCAACTCATTCCCCATCAT
NC/CN-7-4-L6	ACACGACGCTCTTCCGATCTNNNNCGCCAGCTTTGTGTGTGAGTA	GACGTGTGCTCTTCCGATCTAGGTCAACTCATTCCCCATCAT
NC/CN-7-4-L7	ACACGACGCTCTTCCGATCTNNNNCGCCAGCTTTGTGTGTGAGTA	GACGTGTGCTCTTCCGATCTAGGTCAACTCATTCCCCATCAT
NC/CN-7-4-L8	ACACGACGCTCTTCCGATCTNNNNCGCCAGCTTTGTGTGTGAGTA	GACGTGTGCTCTTCCGATCTAGGTCAACTCATTCCCCATCAT
NC/CN-7-5-L1	ACACGACGCTCTTCCGATCTNNNNAGCTTTGTGTGTGAGTATGCAT	GACGTGTGCTCTTCCGATCTAGGTCAACTCATTCCCCATCAT
NC/CN-7-5-L2	ACACGACGCTCTTCCGATCTNNNNAGCTTTGTGTGTGAGTATGCAT	GACGTGTGCTCTTCCGATCTAGGTCAACTCATTCCCCATCAT
NC/CN-7-5-L3	ACACGACGCTCTTCCGATCTNNNNAGCTTTGTGTGTGAGTATGCAT	GACGTGTGCTCTTCCGATCTAGGTCAACTCATTCCCCATCAT
NC/CN-7-5-L4	ACACGACGCTCTTCCGATCTNNNNAGCTTTGTGTGTGAGTATGCAT	GACGTGTGCTCTTCCGATCTAGGTCAACTCATTCCCCATCAT
NC/CN-7-5-L5	ACACGACGCTCTTCCGATCTNNNNAGCTTTGTGTGTGAGTATGCAT	GACGTGTGCTCTTCCGATCTAGGTCAACTCATTCCCCATCAT
NC/CN-7-5-L6	ACACGACGCTCTTCCGATCTNNNNAGCTTTGTGTGTGAGTATGCAT	GACGTGTGCTCTTCCGATCTAGGTCAACTCATTCCCCATCAT
NC/CN-7-5-L7	ACACGACGCTCTTCCGATCTNNNNAGCTTTGTGTGTGAGTATGCAT	GACGTGTGCTCTTCCGATCTAGGTCAACTCATTCCCCATCAT
NC/CN-7-5-L8	ACACGACGCTCTTCCGATCTNNNNAGCTTTGTGTGTGAGTATGCAT	GACGTGTGCTCTTCCGATCTAGGTCAACTCATTCCCCATCAT
NC/CN-7-6-L1	ACACGACGCTCTTCCGATCTNNNNTGTGTGTGAGTATGCATCTCCA	GACGTGTGCTCTTCCGATCTTGGAATCATCTAGGAAGGTCAACT
NC/CN-7-6-L2	ACACGACGCTCTTCCGATCTNNNNTGTGTGTGAGTATGCATCTCCA	GACGTGTGCTCTTCCGATCTTGGAATCATCTAGGAAGGTCAACT
NC/CN-7-6-L3	ACACGACGCTCTTCCGATCTNNNNTGTGTGTGAGTATGCATCTCCA	GACGTGTGCTCTTCCGATCTTGGAATCATCTAGGAAGGTCAACT
NC/CN-7-6-L4	ACACGACGCTCTTCCGATCTNNNNTGTGTGTGAGTATGCATCTCCA	GACGTGTGCTCTTCCGATCTTGGAATCATCTAGGAAGGTCAACT
NC/CN-7-6-L5	ACACGACGCTCTTCCGATCTNNNNTGTGTGTGAGTATGCATCTCCA	GACGTGTGCTCTTCCGATCTTGGAATCATCTAGGAAGGTCAACT
NC/CN-7-6-L6	ACACGACGCTCTTCCGATCTNNNNTGTGTGTGAGTATGCATCTCCA	GACGTGTGCTCTTCCGATCTTGGAATCATCTAGGAAGGTCAACT
NC/CN-7-6-L7	ACACGACGCTCTTCCGATCTNNNNTGTGTGTGAGTATGCATCTCCA	GACGTGTGCTCTTCCGATCTTGGAATCATCTAGGAAGGTCAACT
NC/CN-7-6-L8	ACACGACGCTCTTCCGATCTNNNNTGTGTGTGAGTATGCATCTCCA	GACGTGTGCTCTTCCGATCTTGGAATCATCTAGGAAGGTCAACT
NC/CN-7-7-L1	ACACGACGCTCTTCCGATCTNNNNACCTCCAGTGGAAATCAAGTGA	GACGTGTGCTCTTCCGATCTATAAATCTGGGTTCCGTTGCCT
NC/CN-7-7-L2	ACACGACGCTCTTCCGATCTNNNNACCTCCAGTGGAAATCAAGTGA	GACGTGTGCTCTTCCGATCTATAAATCTGGGTTCCGTTGCCT
NC/CN-7-7-L3	ACACGACGCTCTTCCGATCTNNNNACCTCCAGTGGAAATCAAGTGA	GACGTGTGCTCTTCCGATCTATAAATCTGGGTTCCGTTGCCT
NC/CN-7-7-L4	ACACGACGCTCTTCCGATCTNNNNACCTCCAGTGGAAATCAAGTGA	GACGTGTGCTCTTCCGATCTATAAATCTGGGTTCCGTTGCCT
NC/CN-7-7-L5	ACACGACGCTCTTCCGATCTNNNNACCTCCAGTGGAAATCAAGTGA	GACGTGTGCTCTTCCGATCTATAAATCTGGGTTCCGTTGCCT
NC/CN-7-7-L6	ACACGACGCTCTTCCGATCTNNNNACCTCCAGTGGAAATCAAGTGA	GACGTGTGCTCTTCCGATCTATAAATCTGGGTTCCGTTGCCT
NC/CN-7-7-L7	ACACGACGCTCTTCCGATCTNNNNACCTCCAGTGGAAATCAAGTGA	GACGTGTGCTCTTCCGATCTATAAATCTGGGTTCCGTTGCCT
NC/CN-7-7-L8	ACACGACGCTCTTCCGATCTNNNNACCTCCAGTGGAAATCAAGTGA	GACGTGTGCTCTTCCGATCTATAAATCTGGGTTCCGTTGCCT
NC/CN-7-8-L1	ACACGACGCTCTTCCGATCTNNNNATGGACACGGGACTCTACATCT	GACGTGTGCTCTTCCGATCTGTGTCAACTCAGTGAAATGGCT
NC/CN-7-8-L2	ACACGACGCTCTTCCGATCTNNNNATGGACACGGGACTCTACATCT	GACGTGTGCTCTTCCGATCTGTGTCAACTCAGTGAAATGGCT
NC/CN-7-8-L3	ACACGACGCTCTTCCGATCTNNNNATGGACACGGGACTCTACATCT	GACGTGTGCTCTTCCGATCTGTGTCAACTCAGTGAAATGGCT
NC/CN-7-8-L4	ACACGACGCTCTTCCGATCTNNNNATGGACACGGGACTCTACATCT	GACGTGTGCTCTTCCGATCTGTGTCAACTCAGTGAAATGGCT
NC/CN-7-8-L5	ACACGACGCTCTTCCGATCTNNNNATGGACACGGGACTCTACATCT	GACGTGTGCTCTTCCGATCTGTGTCAACTCAGTGAAATGGCT
NC/CN-7-8-L6	ACACGACGCTCTTCCGATCTNNNNATGGACACGGGACTCTACATCT	GACGTGTGCTCTTCCGATCTGTGTCAACTCAGTGAAATGGCT
NC/CN-7-8-L7	ACACGACGCTCTTCCGATCTNNNNATGGACACGGGACTCTACATCT	GACGTGTGCTCTTCCGATCTGTGTCAACTCAGTGAAATGGCT
NC/CN-7-8-L8	ACACGACGCTCTTCCGATCTNNNNATGGACACGGGACTCTACATCT	GACGTGTGCTCTTCCGATCTGTGTCAACTCAGTGAAATGGCT
NC/CN-7-9-L1	ACACGACGCTCTTCCGATCTNNNNATGGACACGGGACTCTACATCT	GACGTGTGCTCTTCCGATCTGTGTCAACTCAGTGAAATGGCT
NC/CN-7-9-L2	ACACGACGCTCTTCCGATCTNNNNATGGACACGGGACTCTACATCT	GACGTGTGCTCTTCCGATCTGTGTCAACTCAGTGAAATGGCT
NC/CN-7-9-L3	ACACGACGCTCTTCCGATCTNNNNATGGACACGGGACTCTACATCT	GACGTGTGCTCTTCCGATCTGTGTCAACTCAGTGAAATGGCT
NC/CN-7-9-L4	ACACGACGCTCTTCCGATCTNNNNATGGACACGGGACTCTACATCT	GACGTGTGCTCTTCCGATCTGTGTCAACTCAGTGAAATGGCT
NC/CN-7-9-L5	ACACGACGCTCTTCCGATCTNNNNATGGACACGGGACTCTACATCT	GACGTGTGCTCTTCCGATCTGTGTCAACTCAGTGAAATGGCT
NC/CN-7-9-L6	ACACGACGCTCTTCCGATCTNNNNATGGACACGGGACTCTACATCT	GACGTGTGCTCTTCCGATCTGTGTCAACTCAGTGAAATGGCT

NC/CN-7-9-L7	ACACGACGCTCTTCCGATCTNNNNATGGACACGGGACTCTACATCT	GACGTGTGCTCTTCCGATCTGTGTCAACTCAGTGAAATGGCT
NC/CN-7-9-L8	ACACGACGCTCTTCCGATCTNNNNATGGACACGGGACTCTACATCT	GACGTGTGCTCTTCCGATCTGTGTCAACTCAGTGAAATGGCT
NC/CN-7-10-L1	ACACGACGCTCTTCCGATCTNNNNCATGGACACGGGACTCTACATC	GACGTGTGCTCTTCCGATCTAATGCAACAGGTGTCAACTCAG
NC/CN-7-10-L2	ACACGACGCTCTTCCGATCTNNNNCATGGACACGGGACTCTACATC	GACGTGTGCTCTTCCGATCTAATGCAACAGGTGTCAACTCAG
NC/CN-7-10-L3	ACACGACGCTCTTCCGATCTNNNNCATGGACACGGGACTCTACATC	GACGTGTGCTCTTCCGATCTAATGCAACAGGTGTCAACTCAG
NC/CN-7-10-L4	ACACGACGCTCTTCCGATCTNNNNCATGGACACGGGACTCTACATC	GACGTGTGCTCTTCCGATCTAATGCAACAGGTGTCAACTCAG
NC/CN-7-10-L5	ACACGACGCTCTTCCGATCTNNNNCATGGACACGGGACTCTACATC	GACGTGTGCTCTTCCGATCTAATGCAACAGGTGTCAACTCAG
NC/CN-7-10-L6	ACACGACGCTCTTCCGATCTNNNNCATGGACACGGGACTCTACATC	GACGTGTGCTCTTCCGATCTAATGCAACAGGTGTCAACTCAG
NC/CN-7-10-L7	ACACGACGCTCTTCCGATCTNNNNCATGGACACGGGACTCTACATC	GACGTGTGCTCTTCCGATCTAATGCAACAGGTGTCAACTCAG
NC/CN-7-10-L8	ACACGACGCTCTTCCGATCTNNNNCATGGACACGGGACTCTACATC	GACGTGTGCTCTTCCGATCTAATGCAACAGGTGTCAACTCAG

Supplementary Fig. 9

AAVS1 locus - all samples	ACACGACGCTCTTCCGATCTNNNNGTGTGTCACCAGATAAGGAAT	GACGTGTGCTCTTCCGATCTGGCTCTGGTTCTGGGTACTTTTA

Supplementary Fig. 10

5-1-N6a	ACACGACGCTCTTCCGATCTNNNNGGTTAGACCCAATATCAGGAGAC	GACGTGTGCTCTTCCGATCTGCTTTTCTGTCACCAATCCTGTC
5-1-N6b	ACACGACGCTCTTCCGATCTNNNNGGTTAGACCCAATATCAGGAGAC	GACGTGTGCTCTTCCGATCTGCTTTTCTGTCACCAATCCTGTC
5-2-N6a	ACACGACGCTCTTCCGATCTNNNNGGTGTCCGTGTGGAAAACTCC	GACGTGTGCTCTTCCGATCTGGAATATGTCCCAGATAGCACTG
5-2-N6b	ACACGACGCTCTTCCGATCTNNNNGGTGTCCGTGTGGAAAACTCC	GACGTGTGCTCTTCCGATCTGGAATATGTCCCAGATAGCACTG
5-3-N6a	ACACGACGCTCTTCCGATCTNNNNCTGGGTTTGAGTCCTTGGCA	GACGTGTGCTCTTCCGATCTCAGGGGAACGGGGATGCAG
5-3-N6b	ACACGACGCTCTTCCGATCTNNNNCTGGGTTTGAGTCCTTGGCA	GACGTGTGCTCTTCCGATCTCAGGGGAACGGGGATGCAG
5-4-N6a	ACACGACGCTCTTCCGATCTNNNNTGTCTACCAGGCTGCCTT	GACGTGTGCTCTTCCGATCTCCACACCCAGACCTGACCC
5-4-N6b	ACACGACGCTCTTCCGATCTNNNNTGTCTACCAGGCTGCCTT	GACGTGTGCTCTTCCGATCTCCACACCCAGACCTGACCC
5-5-N6a	ACACGACGCTCTTCCGATCTNNNNAGGTCCCCAAGCATCGCC	GACGTGTGCTCTTCCGATCTAGCCCCTCCTACTCTAGCCC
5-5-N6b	ACACGACGCTCTTCCGATCTNNNNAGGTCCCCAAGCATCGCC	GACGTGTGCTCTTCCGATCTAGCCCCTCCTACTCTAGCCC
5-6-N6a	ACACGACGCTCTTCCGATCTNNNNCTGGCCTCCTGGGTTGAATG	GACGTGTGCTCTTCCGATCTCAGTACAGGCATCCCTGTGA
5-6-N6b	ACACGACGCTCTTCCGATCTNNNNCTGGCCTCCTGGGTTGAATG	GACGTGTGCTCTTCCGATCTCAGTACAGGCATCCCTGTGA
5-7-N6a	ACACGACGCTCTTCCGATCTNNNNGGAAAGGCCCATTGCTCTCCTT	GACGTGTGCTCTTCCGATCTCATGGAAACTCGGGCTGTGAAG
5-7-N6b	ACACGACGCTCTTCCGATCTNNNNGGAAAGGCCCATTGCTCTCCTT	GACGTGTGCTCTTCCGATCTCATGGAAACTCGGGCTGTGAAG
5-8-N6a	ACACGACGCTCTTCCGATCTNNNNCCTCCTCCAGATGGGCAGCT	GACGTGTGCTCTTCCGATCTCTGAACTGGAGCTGAGGAAGG
5-8-N6b	ACACGACGCTCTTCCGATCTNNNNCCTCCTCCAGATGGGCAGCT	GACGTGTGCTCTTCCGATCTCTGAACTGGAGCTGAGGAAGG
5-9-N6a	ACACGACGCTCTTCCGATCTNNNNCCCTGGACTTCGGCTTTTGTCC	GACGTGTGCTCTTCCGATCTGCTGTCACACTCCAGTTCACTGA
5-9-N6b	ACACGACGCTCTTCCGATCTNNNNCCCTGGACTTCGGCTTTTGTCC	GACGTGTGCTCTTCCGATCTGCTGTCACACTCCAGTTCACTGA
5-10-N6a	ACACGACGCTCTTCCGATCTNNNNGCTGGGTTTAAGGCAGGAG	GACGTGTGCTCTTCCGATCTTCTCATCTGGGTGCGGGA
5-10-N6b	ACACGACGCTCTTCCGATCTNNNNGCTGGGTTTAAGGCAGGAG	GACGTGTGCTCTTCCGATCTTCTCATCTGGGTGCGGGA
5-11-N6a	ACACGACGCTCTTCCGATCTNNNNAGGCGTATCTTAAACAGCCAGT	GACGTGTGCTCTTCCGATCTGTGGGAAACAGCCGTCAG
5-11-N6b	ACACGACGCTCTTCCGATCTNNNNAGGCGTATCTTAAACAGCCAGT	GACGTGTGCTCTTCCGATCTGTGGGAAACAGCCGTCAG
6-1-N6a	ACACGACGCTCTTCCGATCTNNNNGCTTACGATGGAGCCAGAGAG	GACGTGTGCTCTTCCGATCTGTCAGAGCAGCTCAGGTTCTG
6-1-N6b	ACACGACGCTCTTCCGATCTNNNNGCTTACGATGGAGCCAGAGAG	GACGTGTGCTCTTCCGATCTGTCAGAGCAGCTCAGGTTCTG
6-1-N7a	ACACGACGCTCTTCCGATCTNNNNGCTTACGATGGAGCCAGAGAG	GACGTGTGCTCTTCCGATCTGTCAGAGCAGCTCAGGTTCTG
6-1-N7b	ACACGACGCTCTTCCGATCTNNNNGCTTACGATGGAGCCAGAGAG	GACGTGTGCTCTTCCGATCTGTCAGAGCAGCTCAGGTTCTG
6-2-N6a	ACACGACGCTCTTCCGATCTNNNNGGTGTCCGTGTGGAAAACTCC	GACGTGTGCTCTTCCGATCTGGAATATGTCCCAGATAGCACTG
6-2-N6b	ACACGACGCTCTTCCGATCTNNNNGGTGTCCGTGTGGAAAACTCC	GACGTGTGCTCTTCCGATCTGGAATATGTCCCAGATAGCACTG
6-2-N7a	ACACGACGCTCTTCCGATCTNNNNGGTGTCCGTGTGGAAAACTCC	GACGTGTGCTCTTCCGATCTGGAATATGTCCCAGATAGCACTG
6-2-N7b	ACACGACGCTCTTCCGATCTNNNNGGTGTCCGTGTGGAAAACTCC	GACGTGTGCTCTTCCGATCTGGAATATGTCCCAGATAGCACTG
6-3-N6a	ACACGACGCTCTTCCGATCTNNNNCTGGGTTTGAGTCCTTGGCA	GACGTGTGCTCTTCCGATCTCAGGGGAACGGGGATGCAG
6-3-N6b	ACACGACGCTCTTCCGATCTNNNNCTGGGTTTGAGTCCTTGGCA	GACGTGTGCTCTTCCGATCTCAGGGGAACGGGGATGCAG
6-3-N7a	ACACGACGCTCTTCCGATCTNNNNCTGGGTTTGAGTCCTTGGCA	GACGTGTGCTCTTCCGATCTCAGGGGAACGGGGATGCAG
6-3-N7b	ACACGACGCTCTTCCGATCTNNNNCTGGGTTTGAGTCCTTGGCA	GACGTGTGCTCTTCCGATCTCAGGGGAACGGGGATGCAG
6-4-N6a	ACACGACGCTCTTCCGATCTNNNNCAGAGGCCCCAGGCCACCTA	GACGTGTGCTCTTCCGATCTCCACAGTTGGAGGAGAATCCA
6-4-N6b	ACACGACGCTCTTCCGATCTNNNNCAGAGGCCCCAGGCCACCTA	GACGTGTGCTCTTCCGATCTCCACAGTTGGAGGAGAATCCA
6-4-N7a	ACACGACGCTCTTCCGATCTNNNNCAGAGGCCCCAGGCCACCTA	GACGTGTGCTCTTCCGATCTCCACAGTTGGAGGAGAATCCA
6-4-N7b	ACACGACGCTCTTCCGATCTNNNNCAGAGGCCCCAGGCCACCTA	GACGTGTGCTCTTCCGATCTCCACAGTTGGAGGAGAATCCA
6-5-N6a	ACACGACGCTCTTCCGATCTNNNNTGTCTACCAGGCTGCCTT	GACGTGTGCTCTTCCGATCTCCACACCCAGACCTGACCC
6-5-N6b	ACACGACGCTCTTCCGATCTNNNNTGTCTACCAGGCTGCCTT	GACGTGTGCTCTTCCGATCTCCACACCCAGACCTGACCC
6-5-N7a	ACACGACGCTCTTCCGATCTNNNNTGTCTACCAGGCTGCCTT	GACGTGTGCTCTTCCGATCTCCACACCCAGACCTGACCC
6-5-N7b	ACACGACGCTCTTCCGATCTNNNNTGTCTACCAGGCTGCCTT	GACGTGTGCTCTTCCGATCTCCACACCCAGACCTGACCC
6-6-N6a	ACACGACGCTCTTCCGATCTNNNNCCCCTCCCCTATCGCCAT	GACGTGTGCTCTTCCGATCTCCCCAAGTCCCTCACCTCT
6-6-N6b	ACACGACGCTCTTCCGATCTNNNNCCCCTCCCCTATCGCCAT	GACGTGTGCTCTTCCGATCTCCCCAAGTCCCTCACCTCT
6-6-N7a	ACACGACGCTCTTCCGATCTNNNNCCCCTCCCCTATCGCCAT	GACGTGTGCTCTTCCGATCTCCCCAAGTCCCTCACCTCT
6-6-N7b	ACACGACGCTCTTCCGATCTNNNNCCCCTCCCCTATCGCCAT	GACGTGTGCTCTTCCGATCTCCCCAAGTCCCTCACCTCT
6-7-N6a	ACACGACGCTCTTCCGATCTNNNNCCTCCTCCAGATGGGCAGCT	GACGTGTGCTCTTCCGATCTCTGAACTGGAGCTGAGGAAGG
6-7-N6b	ACACGACGCTCTTCCGATCTNNNNCCTCCTCCAGATGGGCAGCT	GACGTGTGCTCTTCCGATCTCTGAACTGGAGCTGAGGAAGG
6-7-N7a	ACACGACGCTCTTCCGATCTNNNNCCTCCTCCAGATGGGCAGCT	GACGTGTGCTCTTCCGATCTCTGAACTGGAGCTGAGGAAGG
6-7-N7b	ACACGACGCTCTTCCGATCTNNNNCCTCCTCCAGATGGGCAGCT	GACGTGTGCTCTTCCGATCTCTGAACTGGAGCTGAGGAAGG
6-8-N6a	ACACGACGCTCTTCCGATCTNNNNCCCGTGGATCTAGGAGTTTAGC	GACGTGTGCTCTTCCGATCTCCGTTTCTCATTCTTCCCTTAGG
6-8-N6b	ACACGACGCTCTTCCGATCTNNNNCCCGTGGATCTAGGAGTTTAGC	GACGTGTGCTCTTCCGATCTCCGTTTCTCATTCTTCCCTTAGG
6-8-N7a	ACACGACGCTCTTCCGATCTNNNNCCCGTGGATCTAGGAGTTTAGC	GACGTGTGCTCTTCCGATCTCCGTTTCTCATTCTTCCCTTAGG
6-8-N7b	ACACGACGCTCTTCCGATCTNNNNCCCGTGGATCTAGGAGTTTAGC	GACGTGTGCTCTTCCGATCTCCGTTTCTCATTCTTCCCTTAGG
6-9-N6a	ACACGACGCTCTTCCGATCTNNNNCCCTGGACTTCGGCTTTTGTCC	GACGTGTGCTCTTCCGATCTGCTGTCACACTCCAGTTCACTGA
6-9-N6b	ACACGACGCTCTTCCGATCTNNNNCCCTGGACTTCGGCTTTTGTCC	GACGTGTGCTCTTCCGATCTGCTGTCACACTCCAGTTCACTGA
6-9-N7a	ACACGACGCTCTTCCGATCTNNNNCCCTGGACTTCGGCTTTTGTCC	GACGTGTGCTCTTCCGATCTGCTGTCACACTCCAGTTCACTGA
6-9-N7b	ACACGACGCTCTTCCGATCTNNNNCCCTGGACTTCGGCTTTTGTCC	GACGTGTGCTCTTCCGATCTGCTGTCACACTCCAGTTCACTGA
6-10-N6a	ACACGACGCTCTTCCGATCTNNNNCCTCAGTGAACTGGAGTGTGAC	GACGTGTGCTCTTCCGATCTTGGAGGAAGGCCACCCTGTG
6-10-N6b	ACACGACGCTCTTCCGATCTNNNNCCTCAGTGAACTGGAGTGTGAC	GACGTGTGCTCTTCCGATCTTGGAGGAAGGCCACCCTGTG
6-10-N7a	ACACGACGCTCTTCCGATCTNNNNCCTCAGTGAACTGGAGTGTGAC	GACGTGTGCTCTTCCGATCTTGGAGGAAGGCCACCCTGTG
6-10-N7b	ACACGACGCTCTTCCGATCTNNNNCCTCAGTGAACTGGAGTGTGAC	GACGTGTGCTCTTCCGATCTTGGAGGAAGGCCACCCTGTG
6-11-N6a	ACACGACGCTCTTCCGATCTNNNNTCGTGGCTCCCTCTGGAGTC	GACGTGTGCTCTTCCGATCTCACCACCTCCAGGTTCTCATCA
6-11-N6b	ACACGACGCTCTTCCGATCTNNNNTCGTGGCTCCCTCTGGAGTC	GACGTGTGCTCTTCCGATCTCACCACCTCCAGGTTCTCATCA
6-11-N7a	ACACGACGCTCTTCCGATCTNNNNTCGTGGCTCCCTCTGGAGTC	GACGTGTGCTCTTCCGATCTCACCACCTCCAGGTTCTCATCA

6-11-N7b	ACACGACGCTCTTCCGATCTNNNNTCGTGGCTCCCTCTGGAGTC	GACGTGTGCTCTTCCGATCTCACCACCTCCAGGTTCTCATCA
7-1-N6a	ACACGACGCTCTTCCGATCTNNNNGGTTCTGGGTACTTTTATCTGTCC	GACGTGTGCTCTTCCGATCTGAATCTGCCTAACAGGAGGTG
7-1-N6b	ACACGACGCTCTTCCGATCTNNNNGGTTCTGGGTACTTTTATCTGTCC	GACGTGTGCTCTTCCGATCTGAATCTGCCTAACAGGAGGTG
7-1-N7a	ACACGACGCTCTTCCGATCTNNNNGGTTCTGGGTACTTTTATCTGTCC	GACGTGTGCTCTTCCGATCTGAATCTGCCTAACAGGAGGTG
7-1-N7b	ACACGACGCTCTTCCGATCTNNNNGGTTCTGGGTACTTTTATCTGTCC	GACGTGTGCTCTTCCGATCTGAATCTGCCTAACAGGAGGTG
7-2-N6a	ACACGACGCTCTTCCGATCTNNNNGCTTACGATGGAGCCAGAGAG	GACGTGTGCTCTTCCGATCTGTCAGAGCAGCTCAGGTTCTG
7-2-N6b	ACACGACGCTCTTCCGATCTNNNNGCTTACGATGGAGCCAGAGAG	GACGTGTGCTCTTCCGATCTGTCAGAGCAGCTCAGGTTCTG
7-2-N7a	ACACGACGCTCTTCCGATCTNNNNGCTTACGATGGAGCCAGAGAG	GACGTGTGCTCTTCCGATCTGTCAGAGCAGCTCAGGTTCTG
7-2-N7b	ACACGACGCTCTTCCGATCTNNNNGCTTACGATGGAGCCAGAGAG	GACGTGTGCTCTTCCGATCTGTCAGAGCAGCTCAGGTTCTG
7-3-N6a	ACACGACGCTCTTCCGATCTNNNNGGTGTCCGTGTGGAAAACTCC	GACGTGTGCTCTTCCGATCTGGAATATGTCCCAGATAGCACTG
7-3-N6b	ACACGACGCTCTTCCGATCTNNNNGGTGTCCGTGTGGAAAACTCC	GACGTGTGCTCTTCCGATCTGGAATATGTCCCAGATAGCACTG
7-3-N7a	ACACGACGCTCTTCCGATCTNNNNGGTGTCCGTGTGGAAAACTCC	GACGTGTGCTCTTCCGATCTGGAATATGTCCCAGATAGCACTG
7-3-N7b	ACACGACGCTCTTCCGATCTNNNNGGTGTCCGTGTGGAAAACTCC	GACGTGTGCTCTTCCGATCTGGAATATGTCCCAGATAGCACTG
7-4-N6a	ACACGACGCTCTTCCGATCTNNNNCTGGGTTTGAGTCCTTGGCA	GACGTGTGCTCTTCCGATCTCAGGGGAACGGGGATGCAG
7-4-N6b	ACACGACGCTCTTCCGATCTNNNNCTGGGTTTGAGTCCTTGGCA	GACGTGTGCTCTTCCGATCTCAGGGGAACGGGGATGCAG
7-4-N7a	ACACGACGCTCTTCCGATCTNNNNCTGGGTTTGAGTCCTTGGCA	GACGTGTGCTCTTCCGATCTCAGGGGAACGGGGATGCAG
7-4-N7b	ACACGACGCTCTTCCGATCTNNNNCTGGGTTTGAGTCCTTGGCA	GACGTGTGCTCTTCCGATCTCAGGGGAACGGGGATGCAG
7-5-N6a	ACACGACGCTCTTCCGATCTNNNNCAGAGGCCCCAGGCCACCTA	GACGTGTGCTCTTCCGATCTCCACAGTTGGAGGAGAATCCA
7-5-N6b	ACACGACGCTCTTCCGATCTNNNNCAGAGGCCCCAGGCCACCTA	GACGTGTGCTCTTCCGATCTCCACAGTTGGAGGAGAATCCA
7-5-N7a	ACACGACGCTCTTCCGATCTNNNNCAGAGGCCCCAGGCCACCTA	GACGTGTGCTCTTCCGATCTCCACAGTTGGAGGAGAATCCA
7-5-N7b	ACACGACGCTCTTCCGATCTNNNNCAGAGGCCCCAGGCCACCTA	GACGTGTGCTCTTCCGATCTCCACAGTTGGAGGAGAATCCA
7-6-N6a	ACACGACGCTCTTCCGATCTNNNNTGTCTACCAGGCTGCCTT	GACGTGTGCTCTTCCGATCTCCACACCCAGACCTGACCC
7-6-N6b	ACACGACGCTCTTCCGATCTNNNNTGTCTACCAGGCTGCCTT	GACGTGTGCTCTTCCGATCTCCACACCCAGACCTGACCC
7-6-N7a	ACACGACGCTCTTCCGATCTNNNNTGTCTACCAGGCTGCCTT	GACGTGTGCTCTTCCGATCTCCACACCCAGACCTGACCC
7-6-N7b	ACACGACGCTCTTCCGATCTNNNNTGTCTACCAGGCTGCCTT	GACGTGTGCTCTTCCGATCTCCACACCCAGACCTGACCC
7-7-N6a	ACACGACGCTCTTCCGATCTNNNNCTCCTGGGTTTGAGAGAGGAG	GACGTGTGCTCTTCCGATCTCTCAGACCCACGAGTCCAGG
7-7-N6b	ACACGACGCTCTTCCGATCTNNNNCTCCTGGGTTTGAGAGAGGAG	GACGTGTGCTCTTCCGATCTCTCAGACCCACGAGTCCAGG
7-7-N7a	ACACGACGCTCTTCCGATCTNNNNCTCCTGGGTTTGAGAGAGGAG	GACGTGTGCTCTTCCGATCTCTCAGACCCACGAGTCCAGG
7-7-N7b	ACACGACGCTCTTCCGATCTNNNNCTCCTGGGTTTGAGAGAGGAG	GACGTGTGCTCTTCCGATCTCTCAGACCCACGAGTCCAGG
7-8-N6a	ACACGACGCTCTTCCGATCTNNNNCTGGCCTCCTGGGTTGAATG	GACGTGTGCTCTTCCGATCTCAGTACAGGCATCCCTGTGA
7-8-N6b	ACACGACGCTCTTCCGATCTNNNNCTGGCCTCCTGGGTTGAATG	GACGTGTGCTCTTCCGATCTCAGTACAGGCATCCCTGTGA
7-8-N7a	ACACGACGCTCTTCCGATCTNNNNCTGGCCTCCTGGGTTGAATG	GACGTGTGCTCTTCCGATCTCAGTACAGGCATCCCTGTGA
7-8-N7b	ACACGACGCTCTTCCGATCTNNNNCTGGCCTCCTGGGTTGAATG	GACGTGTGCTCTTCCGATCTCAGTACAGGCATCCCTGTGA
7-9-N6a	ACACGACGCTCTTCCGATCTNNNNGGAAAGGCCCATTGCTCTCCTT	GACGTGTGCTCTTCCGATCTCATGGAAACTCGGGCTGTGAAG
7-9-N6b	ACACGACGCTCTTCCGATCTNNNNGGAAAGGCCCATTGCTCTCCTT	GACGTGTGCTCTTCCGATCTCATGGAAACTCGGGCTGTGAAG
7-9-N7a	ACACGACGCTCTTCCGATCTNNNNGGAAAGGCCCATTGCTCTCCTT	GACGTGTGCTCTTCCGATCTCATGGAAACTCGGGCTGTGAAG
7-9-N7b	ACACGACGCTCTTCCGATCTNNNNGGAAAGGCCCATTGCTCTCCTT	GACGTGTGCTCTTCCGATCTCATGGAAACTCGGGCTGTGAAG
7-10-N6a	ACACGACGCTCTTCCGATCTNNNNCCCGTGGATCTAGGAGTTTAGC	GACGTGTGCTCTTCCGATCTCCGTTTCTCATTCTTCCCTTAGG
7-10-N6b	ACACGACGCTCTTCCGATCTNNNNCCCGTGGATCTAGGAGTTTAGC	GACGTGTGCTCTTCCGATCTCCGTTTCTCATTCTTCCCTTAGG
7-10-N7a	ACACGACGCTCTTCCGATCTNNNNCCCGTGGATCTAGGAGTTTAGC	GACGTGTGCTCTTCCGATCTCCGTTTCTCATTCTTCCCTTAGG
7-10-N7b	ACACGACGCTCTTCCGATCTNNNNCCCGTGGATCTAGGAGTTTAGC	GACGTGTGCTCTTCCGATCTCCGTTTCTCATTCTTCCCTTAGG
7-11-N6a	ACACGACGCTCTTCCGATCTNNNNGCTGGGTTTAAGGCAGGAG	GACGTGTGCTCTTCCGATCTTCTCATCTGGGTGCGGGA
7-11-N6b	ACACGACGCTCTTCCGATCTNNNNGCTGGGTTTAAGGCAGGAG	GACGTGTGCTCTTCCGATCTTCTCATCTGGGTGCGGGA
7-11-N7a	ACACGACGCTCTTCCGATCTNNNNGCTGGGTTTAAGGCAGGAG	GACGTGTGCTCTTCCGATCTTCTCATCTGGGTGCGGGA
7-11-N7b	ACACGACGCTCTTCCGATCTNNNNGCTGGGTTTAAGGCAGGAG	GACGTGTGCTCTTCCGATCTTCTCATCTGGGTGCGGGA
8-1-N6a	ACACGACGCTCTTCCGATCTNNNNGGTTAGACCCAATATCAGGAGAC	GACGTGTGCTCTTCCGATCTGCTTTTCTGTCACCAATCCTGTC
8-1-N6b	ACACGACGCTCTTCCGATCTNNNNGGTTAGACCCAATATCAGGAGAC	GACGTGTGCTCTTCCGATCTGCTTTTCTGTCACCAATCCTGTC
8-1-N7a	ACACGACGCTCTTCCGATCTNNNNGGTTAGACCCAATATCAGGAGAC	GACGTGTGCTCTTCCGATCTGCTTTTCTGTCACCAATCCTGTC
8-1-N7b	ACACGACGCTCTTCCGATCTNNNNGGTTAGACCCAATATCAGGAGAC	GACGTGTGCTCTTCCGATCTGCTTTTCTGTCACCAATCCTGTC
8-2-N6a	ACACGACGCTCTTCCGATCTNNNNGGTTCTGGGTACTTTTATCTGTCC	GACGTGTGCTCTTCCGATCTGAATCTGCCTAACAGGAGGTG
8-2-N6b	ACACGACGCTCTTCCGATCTNNNNGGTTCTGGGTACTTTTATCTGTCC	GACGTGTGCTCTTCCGATCTGAATCTGCCTAACAGGAGGTG
8-2-N7a	ACACGACGCTCTTCCGATCTNNNNGGTTCTGGGTACTTTTATCTGTCC	GACGTGTGCTCTTCCGATCTGAATCTGCCTAACAGGAGGTG
8-2-N7b	ACACGACGCTCTTCCGATCTNNNNGGTTCTGGGTACTTTTATCTGTCC	GACGTGTGCTCTTCCGATCTGAATCTGCCTAACAGGAGGTG
8-3-N6a	ACACGACGCTCTTCCGATCTNNNNGCTTACGATGGAGCCAGAGAG	GACGTGTGCTCTTCCGATCTGTCAGAGCAGCTCAGGTTCTG
8-3-N6b	ACACGACGCTCTTCCGATCTNNNNGCTTACGATGGAGCCAGAGAG	GACGTGTGCTCTTCCGATCTGTCAGAGCAGCTCAGGTTCTG
8-3-N7a	ACACGACGCTCTTCCGATCTNNNNGCTTACGATGGAGCCAGAGAG	GACGTGTGCTCTTCCGATCTGTCAGAGCAGCTCAGGTTCTG
8-3-N7b	ACACGACGCTCTTCCGATCTNNNNGCTTACGATGGAGCCAGAGAG	GACGTGTGCTCTTCCGATCTGTCAGAGCAGCTCAGGTTCTG
8-4-N6a	ACACGACGCTCTTCCGATCTNNNNCTGGGTTTGAGTCCTTGGCA	GACGTGTGCTCTTCCGATCTCAGGGGAACGGGGATGCAG
8-4-N6b	ACACGACGCTCTTCCGATCTNNNNCTGGGTTTGAGTCCTTGGCA	GACGTGTGCTCTTCCGATCTCAGGGGAACGGGGATGCAG
8-4-N7a	ACACGACGCTCTTCCGATCTNNNNCTGGGTTTGAGTCCTTGGCA	GACGTGTGCTCTTCCGATCTCAGGGGAACGGGGATGCAG
8-4-N7b	ACACGACGCTCTTCCGATCTNNNNCTGGGTTTGAGTCCTTGGCA	GACGTGTGCTCTTCCGATCTCAGGGGAACGGGGATGCAG
8-5-N6a	ACACGACGCTCTTCCGATCTNNNNCAGAGGCCCCAGGCCACCTA	GACGTGTGCTCTTCCGATCTCCACAGTTGGAGGAGAATCCA
8-5-N6b	ACACGACGCTCTTCCGATCTNNNNCAGAGGCCCCAGGCCACCTA	GACGTGTGCTCTTCCGATCTCCACAGTTGGAGGAGAATCCA
8-5-N7a	ACACGACGCTCTTCCGATCTNNNNCAGAGGCCCCAGGCCACCTA	GACGTGTGCTCTTCCGATCTCCACAGTTGGAGGAGAATCCA
8-5-N7b	ACACGACGCTCTTCCGATCTNNNNCAGAGGCCCCAGGCCACCTA	GACGTGTGCTCTTCCGATCTCCACAGTTGGAGGAGAATCCA
8-6-N6a	ACACGACGCTCTTCCGATCTNNNNTGTCTACCAGGCTGCCTT	GACGTGTGCTCTTCCGATCTCCACACCCAGACCTGACCC
8-6-N6b	ACACGACGCTCTTCCGATCTNNNNTGTCTACCAGGCTGCCTT	GACGTGTGCTCTTCCGATCTCCACACCCAGACCTGACCC
8-6-N7a	ACACGACGCTCTTCCGATCTNNNNTGTCTACCAGGCTGCCTT	GACGTGTGCTCTTCCGATCTCCACACCCAGACCTGACCC
8-6-N7b	ACACGACGCTCTTCCGATCTNNNNTGTCTACCAGGCTGCCTT	GACGTGTGCTCTTCCGATCTCCACACCCAGACCTGACCC
8-7-N6a	ACACGACGCTCTTCCGATCTNNNNCTCCTGGGTTTGAGAGAGGAG	GACGTGTGCTCTTCCGATCTCTCAGACCCACGAGTCCAGG
8-7-N6b	ACACGACGCTCTTCCGATCTNNNNCTCCTGGGTTTGAGAGAGGAG	GACGTGTGCTCTTCCGATCTCTCAGACCCACGAGTCCAGG
8-7-N7a	ACACGACGCTCTTCCGATCTNNNNCTCCTGGGTTTGAGAGAGGAG	GACGTGTGCTCTTCCGATCTCTCAGACCCACGAGTCCAGG
8-7-N7b	ACACGACGCTCTTCCGATCTNNNNCTCCTGGGTTTGAGAGAGGAG	GACGTGTGCTCTTCCGATCTCTCAGACCCACGAGTCCAGG
8-8-N6a	ACACGACGCTCTTCCGATCTNNNNCATCTTTCACAGGGATGCCTGT	GACGTGTGCTCTTCCGATCTCACCCAGTTGTCATGGCGATAG
8-8-N6b	ACACGACGCTCTTCCGATCTNNNNCATCTTTCACAGGGATGCCTGT	GACGTGTGCTCTTCCGATCTCACCCAGTTGTCATGGCGATAG
8-8-N7a	ACACGACGCTCTTCCGATCTNNNNCATCTTTCACAGGGATGCCTGT	GACGTGTGCTCTTCCGATCTCACCCAGTTGTCATGGCGATAG
8-8-N7b	ACACGACGCTCTTCCGATCTNNNNCATCTTTCACAGGGATGCCTGT	GACGTGTGCTCTTCCGATCTCACCCAGTTGTCATGGCGATAG
8-9-N6a	ACACGACGCTCTTCCGATCTNNNNCCCCTCCCCTATCGCCAT	GACGTGTGCTCTTCCGATCTCCCCAAGTCCCTCACCTCT
8-9-N6b	ACACGACGCTCTTCCGATCTNNNNCCCCTCCCCTATCGCCAT	GACGTGTGCTCTTCCGATCTCCCCAAGTCCCTCACCTCT
8-9-N7a	ACACGACGCTCTTCCGATCTNNNNCCCCTCCCCTATCGCCAT	GACGTGTGCTCTTCCGATCTCCCCAAGTCCCTCACCTCT

8-9-N7b	ACACGACGCTCTTCCGATCTNNNNCCCCTCCCCTATCGCCAT	GACGTGTGCTCTTCCGATCTCCCCAAGTCCCTCACCTCT
8-10-N6a	ACACGACGCTCTTCCGATCTNNNNGCTGGGTTTAAGGCAGGAG	GACGTGTGCTCTTCCGATCTTCTCATCTGGGTGCGGGA
8-10-N6b	ACACGACGCTCTTCCGATCTNNNNGCTGGGTTTAAGGCAGGAG	GACGTGTGCTCTTCCGATCTTCTCATCTGGGTGCGGGA
8-10-N7a	ACACGACGCTCTTCCGATCTNNNNGCTGGGTTTAAGGCAGGAG	GACGTGTGCTCTTCCGATCTTCTCATCTGGGTGCGGGA
8-10-N7b	ACACGACGCTCTTCCGATCTNNNNGCTGGGTTTAAGGCAGGAG	GACGTGTGCTCTTCCGATCTTCTCATCTGGGTGCGGGA
8-11-N6a	ACACGACGCTCTTCCGATCTNNNNAGGCGTATCTTAAACAGCCAGT	GACGTGTGCTCTTCCGATCTGTGGGAAACAGCCGTCAG
8-11-N6b	ACACGACGCTCTTCCGATCTNNNNAGGCGTATCTTAAACAGCCAGT	GACGTGTGCTCTTCCGATCTGTGGGAAACAGCCGTCAG
8-11-N7a	ACACGACGCTCTTCCGATCTNNNNAGGCGTATCTTAAACAGCCAGT	GACGTGTGCTCTTCCGATCTGTGGGAAACAGCCGTCAG
8-11-N7b	ACACGACGCTCTTCCGATCTNNNNAGGCGTATCTTAAACAGCCAGT	GACGTGTGCTCTTCCGATCTGTGGGAAACAGCCGTCAG
9-1-N7a	ACACGACGCTCTTCCGATCTNNNNTCCTTTCATTTGGGCAGCTCC	GACGTGTGCTCTTCCGATCTCCCTGGAGGCAGCAAACA
9-1-N7b	ACACGACGCTCTTCCGATCTNNNNTCCTTTCATTTGGGCAGCTCC	GACGTGTGCTCTTCCGATCTCCCTGGAGGCAGCAAACA
9-2-N7a	ACACGACGCTCTTCCGATCTNNNNGGTGTCCGTGTGGAAAACTCC	GACGTGTGCTCTTCCGATCTGGAATATGTCCCAGATAGCACTG
9-2-N7b	ACACGACGCTCTTCCGATCTNNNNGGTGTCCGTGTGGAAAACTCC	GACGTGTGCTCTTCCGATCTGGAATATGTCCCAGATAGCACTG
9-3-N7a	ACACGACGCTCTTCCGATCTNNNNCTGGGTTTGAGTCCTTGGCA	GACGTGTGCTCTTCCGATCTCAGGGGAACGGGGATGCAG
9-3-N7b	ACACGACGCTCTTCCGATCTNNNNCTGGGTTTGAGTCCTTGGCA	GACGTGTGCTCTTCCGATCTCAGGGGAACGGGGATGCAG
9-4-N7a	ACACGACGCTCTTCCGATCTNNNNCAGAGGCCCCAGGCCACCTA	GACGTGTGCTCTTCCGATCTCCACAGTTGGAGGAGAATCCA
9-4-N7b	ACACGACGCTCTTCCGATCTNNNNCAGAGGCCCCAGGCCACCTA	GACGTGTGCTCTTCCGATCTCCACAGTTGGAGGAGAATCCA
9-5-N7a	ACACGACGCTCTTCCGATCTNNNNTGTCTACCAGGCTGCCTT	GACGTGTGCTCTTCCGATCTCCACACCCAGACCTGACCC
9-5-N7b	ACACGACGCTCTTCCGATCTNNNNTGTCTACCAGGCTGCCTT	GACGTGTGCTCTTCCGATCTCCACACCCAGACCTGACCC
9-6-N7a	ACACGACGCTCTTCCGATCTNNNNGATGGTTGGTCACTGTCTCTG	GACGTGTGCTCTTCCGATCTCTAAGAACTTGGGAACAGCCAC
9-6-N7b	ACACGACGCTCTTCCGATCTNNNNGATGGTTGGTCACTGTCTCTG	GACGTGTGCTCTTCCGATCTCTAAGAACTTGGGAACAGCCAC
9-7-N7a	ACACGACGCTCTTCCGATCTNNNNCTCCTGGGTTTGAGAGAGGAG	GACGTGTGCTCTTCCGATCTCTCAGACCCACGAGTCCAGG
9-7-N7b	ACACGACGCTCTTCCGATCTNNNNCTCCTGGGTTTGAGAGAGGAG	GACGTGTGCTCTTCCGATCTCTCAGACCCACGAGTCCAGG
9-8-N7a	ACACGACGCTCTTCCGATCTNNNNGGAAAGGCCCATTGCTCTCCTT	GACGTGTGCTCTTCCGATCTCATGGAAACTCGGGCTGTGAAG
9-8-N7b	ACACGACGCTCTTCCGATCTNNNNGGAAAGGCCCATTGCTCTCCTT	GACGTGTGCTCTTCCGATCTCATGGAAACTCGGGCTGTGAAG
9-9-N7a	ACACGACGCTCTTCCGATCTNNNNCCCTGGACTTCGGCTTTTGTCC	GACGTGTGCTCTTCCGATCTGCTGTCACACTCCAGTTCACTGA
9-9-N7b	ACACGACGCTCTTCCGATCTNNNNCCCTGGACTTCGGCTTTTGTCC	GACGTGTGCTCTTCCGATCTGCTGTCACACTCCAGTTCACTGA
9-10-N7a	ACACGACGCTCTTCCGATCTNNNNCCTCAGTGAACTGGAGTGTGAC	GACGTGTGCTCTTCCGATCTTGGAGGAAGGCCACCCTGTG
9-10-N7b	ACACGACGCTCTTCCGATCTNNNNCCTCAGTGAACTGGAGTGTGAC	GACGTGTGCTCTTCCGATCTTGGAGGAAGGCCACCCTGTG
9-11-N7a	ACACGACGCTCTTCCGATCTNNNNGCTGGGTTTAAGGCAGGAG	GACGTGTGCTCTTCCGATCTTCTCATCTGGGTGCGGGA
9-11-N7b	ACACGACGCTCTTCCGATCTNNNNGCTGGGTTTAAGGCAGGAG	GACGTGTGCTCTTCCGATCTTCTCATCTGGGTGCGGGA

Supplementary Fig. 17

NoSkip-1	ACACGACGCTCTTCCGATCTNNNNAACGGGGAAGGAGGATGCC	GACGTGTGCTCTTCCGATCTGACTCCACCAACGCCGAC
NoSkip-2	ACACGACGCTCTTCCGATCTNNNNGAGGGCAGCGCAAAGTGAC	GACGTGTGCTCTTCCGATCTCTTCCGATGTTGAGCCCCTC
NoSkip-3	ACACGACGCTCTTCCGATCTNNNNGCCCAAATGAAAGGAGTGAGAG	GACGTGTGCTCTTCCGATCTCAAAGTACCCCGTCTCCCTG
NoSkip-4	ACACGACGCTCTTCCGATCTNNNNAAACATGCTGTCCTGAAGTGG	GACGTGTGCTCTTCCGATCTCCCCTTACCTCTCTAGTCT
NoSkip-5	ACACGACGCTCTTCCGATCTNNNNGAGACTAGGAAGGAGGAGGCC	GACGTGTGCTCTTCCGATCTGGTTAATGTGGCTCTGGTTCT
NoSkip-6	ACACGACGCTCTTCCGATCTNNNNCTCCAGCCCCTCCTACTCTAG	GACGTGTGCTCTTCCGATCTGGCTGTTCCCAAGTTCTTA
NoSkip-7	ACACGACGCTCTTCCGATCTNNNNCTGGGAACGGGATGAACT	GACGTGTGCTCTTCCGATCTCTCAGGCATCTTTCACAGGGATG
NoSkip-8	ACACGACGCTCTTCCGATCTNNNNCATGGAAACTCGGGCTGTGAAG	GACGTGTGCTCTTCCGATCTCAGGCATCTTTCACAGGGATG
NoSkip-9	ACACGACGCTCTTCCGATCTNNNNAGCTAAACTCCTAGATCCACGG	GACGTGTGCTCTTCCGATCTCTTCACAGCCCGAGTTTCCATG
NoSkip-10	ACACGACGCTCTTCCGATCTNNNNCCTCCAGGTTCTCATCAATGC	GACGTGTGCTCTTCCGATCTCACTGTCCTAGCACCGTGTC
Skip-1-1c	ACACGACGCTCTTCCGATCTNNNNGAAGTCGAGGGAGGGATGGTAA	GACGTGTGCTCTTCCGATCTCTGACCACTCTGCCCCAG
Skip-1-LRQEKERP	ACACGACGCTCTTCCGATCTNNNNGAAGTCGAGGGAGGGATGGTAA	GACGTGTGCTCTTCCGATCTCTGACCACTCTGCCCCAG
Skip-2-1c	ACACGACGCTCTTCCGATCTNNNNTAGAGGTGACCCAGGCCACAA	GACGTGTGCTCTTCCGATCTCTTTGAGCTCTACTGGCTTCTG
Skip-2-LRQEKERP	ACACGACGCTCTTCCGATCTNNNNTAGAGGTGACCCAGGCCACAA	GACGTGTGCTCTTCCGATCTCTTTGAGCTCTACTGGCTTCTG
Skip-3-1c	ACACGACGCTCTTCCGATCTNNNNAGTGGAGGAAGACGGAACC	GACGTGTGCTCTTCCGATCTGTCCTGCTTTCTCTGACCTGC
Skip-3-LRQEKERP	ACACGACGCTCTTCCGATCTNNNNAGTGGAGGAAGACGGAACC	GACGTGTGCTCTTCCGATCTGTCCTGCTTTCTCTGACCTGC
Skip-4-1c	ACACGACGCTCTTCCGATCTNNNNGGAGACTAGGAAGGAGGAGGC	GACGTGTGCTCTTCCGATCTCGGTTAATGTGGCTCTGGTT
Skip-4-LRQEKERP	ACACGACGCTCTTCCGATCTNNNNGGAGACTAGGAAGGAGGAGGC	GACGTGTGCTCTTCCGATCTCGGTTAATGTGGCTCTGGTT
Skip-5-1c	ACACGACGCTCTTCCGATCTNNNNCTGGGAACGGGATGAACTC	GACGTGTGCTCTTCCGATCTCTCAGGCATCTTTCACAGGGATG
Skip-5-LRQEKERP	ACACGACGCTCTTCCGATCTNNNNCTGGGAACGGGATGAACTC	GACGTGTGCTCTTCCGATCTCTCAGGCATCTTTCACAGGGATG
Skip-6-1c	ACACGACGCTCTTCCGATCTNNNNAGCTAAACTCCTAGATCCACGG	GACGTGTGCTCTTCCGATCTCTTCACAGCCCGAGTTTCCATG
Skip-6-LRQEKERP	ACACGACGCTCTTCCGATCTNNNNAGCTAAACTCCTAGATCCACGG	GACGTGTGCTCTTCCGATCTCTTCACAGCCCGAGTTTCCATG
Skip-7-1c	ACACGACGCTCTTCCGATCTNNNNGACAAAAGCCGAAGTCCAGG	GACGTGTGCTCTTCCGATCTCAGATGGGCAGCTTTGGAGAG
Skip-7-LRQEKERP	ACACGACGCTCTTCCGATCTNNNNGACAAAAGCCGAAGTCCAGG	GACGTGTGCTCTTCCGATCTCAGATGGGCAGCTTTGGAGAG
Skip-8-1c	ACACGACGCTCTTCCGATCTNNNNCAGGCTGTCACACTCCAGTT	GACGTGTGCTCTTCCGATCTCCCACCCAGTGTCCACAAG
Skip-8-LRQEKERP	ACACGACGCTCTTCCGATCTNNNNCAGGCTGTCACACTCCAGTT	GACGTGTGCTCTTCCGATCTCCCACCCAGTGTCCACAAG
Skip-9-1c	ACACGACGCTCTTCCGATCTNNNNCAGGCTGTCACACTCCAGTTC	GACGTGTGCTCTTCCGATCTGACTTCGGCTTTTGTCCCC
Skip-9-LRQEKERP	ACACGACGCTCTTCCGATCTNNNNCAGGCTGTCACACTCCAGTTC	GACGTGTGCTCTTCCGATCTGACTTCGGCTTTTGTCCCC
Skip-10-1c	ACACGACGCTCTTCCGATCTNNNNCCTCCAGGTTCTCATCAATGC	GACGTGTGCTCTTCCGATCTCACTGTCCTAGCACCGTGTC
Skip-10-LRQEKERP	ACACGACGCTCTTCCGATCTNNNNCCTCCAGGTTCTCATCAATGC	GACGTGTGCTCTTCCGATCTCACTGTCCTAGCACCGTGTC

Supplementary Fig. 20

Nat Methods **9**, 588-590		
1	ACACGACGCTCTTCCGATCTNNNNAATCACAGCCCTCTCGATCGA	GACGTGTGCTCTTCCGATCTAAGAAGAGACTGAGTGCTGGGA
2	ACACGACGCTCTTCCGATCTNNNNTTGTCTATGGGGTTTGAATAAGC	GACGTGTGCTCTTCCGATCTATGTTTATTAAGCCATGCCCTG
3	ACACGACGCTCTTCCGATCTNNNNGGAGGTCCTAACTAAGCCAG	GACGTGTGCTCTTCCGATCTTGACAGCCCATTCTTCTAGTTCT
4	ACACGACGCTCTTCCGATCTNNNNAAGTTCTTTTCATTATAGCATGC	GACGTGTGCTCTTCCGATCTAGTCACTTATTTTCTAAAGGTTACC
5	ACACGACGCTCTTCCGATCTNNNNCTCAACTCAAATGTTCCTCCCTC	GACGTGTGCTCTTCCGATCTACAGTGGTTTAAACTAAGATGCAA
6	ACACGACGCTCTTCCGATCTNNNNGCTGAAGGAGGTGTCACTAT	GACGTGTGCTCTTCCGATCTACGTAACTGAAGTAGTGGAAC
7	ACACGACGCTCTTCCGATCTNNNNTGCTGAAGGAGGTGTCACTATTA	GACGTGTGCTCTTCCGATCTGAACTTTCTCTTCCATGGCAAC
8	ACACGACGCTCTTCCGATCTNNNNGGCATCTAAACATTGCTCAGACC	GACGTGTGCTCTTCCGATCTCAGAGGGACAAATATGCTAAAA
9	ACACGACGCTCTTCCGATCTNNNNTTGCAGTGAGCCGAGATC	GACGTGTGCTCTTCCGATCTGTCATTTCGTGTGTCCAGC
10	ACACGACGCTCTTCCGATCTNNNNAGTGCTGGAAAGTTTATGTACCA	GACGTGTGCTCTTCCGATCTATGCCTCAAAGTTGTCAACCAC
11	ACACGACGCTCTTCCGATCTNNNNAGACATGGAAGAGAAATATGCAGAT	GACGTGTGCTCTTCCGATCTCCCAGTGGTTACAACATGTCTT
12	ACACGACGCTCTTCCGATCTNNNNCCCCTCCAATCTTCCAAGTTAC	GACGTGTGCTCTTCCGATCTTGTCTGTCATGTTCATCACTGC
13	ACACGACGCTCTTCCGATCTNNNNTCAGACATGGAAAAGCAGCAAA	GACGTGTGCTCTTCCGATCTGCCAAACATTAGTCATCGTTATAT
14	ACACGACGCTCTTCCGATCTNNNNGTGTCTAACAGGCCTGAC	GACGTGTGCTCTTCCGATCTAAGCCTGAGGTGGTAGAACATC
15	ACACGACGCTCTTCCGATCTNNNNGTCTGGGAGGTCATTAACATGTT	GACGTGTGCTCTTCCGATCTAGAGAGAGAGGACAACGAAGAG
PNAS **96**, 2758-2763		
1	ACACGACGCTCTTCCGATCTNNNNCTCCTTAAGTGGCCGCGCAG	GACGTGTGCTCTTCCGATCTGATCGGCCATGGTCCCTC
2	ACACGACGCTCTTCCGATCTNNNNATGAAACAGATGAAGAGTGAGCT	GACGTGTGCTCTTCCGATCTGTGGGTTTTAGTAGTGTTTCAAGG
3	ACACGACGCTCTTCCGATCTNNNNCAATTCCTAAGTACTTCTGTAGCCC	GACGTGTGCTCTTCCGATCTGAAACCTGTTCCTGAGTAAATGT
4	ACACGACGCTCTTCCGATCTNNNNACGGTCATTTTCTGTTAGCTTTGTA	GACGTGTGCTCTTCCGATCTTAAATCTTCCTTTCAAGCCTTT
5	ACACGACGCTCTTCCGATCTNNNNTAGAGTCTACAGGGGAGAAGG	GACGTGTGCTCTTCCGATCTTGCCTTTACAATGTATCCGGAAT
6	ACACGACGCTCTTCCGATCTNNNNGTCATATTTGGCTGCCTTTTCA	GACGTGTGCTCTTCCGATCTATAGTTCCCCATTAGCTACTGAA
7	ACACGACGCTCTTCCGATCTNNNNTAATTGCAATCAGATTCCAGATG	GACGTGTGCTCTTCCGATCTTCTTTTCCTCTTCGGTTTGTTGT
8	ACACGACGCTCTTCCGATCTNNNNTTAGTACAACAGGGAGCATG	GACGTGTGCTCTTCCGATCTGTTTTACTGGGCATTGGAGC
9	ACACGACGCTCTTCCGATCTNNNNGGATTCAAGTTACGCTACTCCAA	GACGTGTGCTCTTCCGATCTTCCTTCCAGTATAGTAAGGGCAC
10	ACACGACGCTCTTCCGATCTNNNNTAGGTAACAAGCGCAGTCAT	GACGTGTGCTCTTCCGATCTTCCCCATTAGCTACTGAAGT
11	ACACGACGCTCTTCCGATCTNNNNGGAACTGTACCTTGCCTTCAC	GACGTGTGCTCTTCCGATCTTGAAGAGAAGGGTATAAGCAGAT
12	ACACGACGCTCTTCCGATCTNNNNAACCTTTTGCAAGCTTCTTCCTT	GACGTGTGCTCTTCCGATCTGCAGATTCATGGTGACTAGCT
13	ACACGACGCTCTTCCGATCTNNNNCTGAAAGCACTGCGGAGGA	GACGTGTGCTCTTCCGATCTAAAGCCCCAACTGCCCAG
14	ACACGACGCTCTTCCGATCTNNNNAGGAGTGAGCTTCCCAGAGC	GACGTGTGCTCTTCCGATCTGTTCCCAGGTCCCTAGGTG
15	ACACGACGCTCTTCCGATCTNNNNTGAATTATGACATCTAGAAGAAGGA	GACGTGTGCTCTTCCGATCTACTGAAGTGATGATTTTAACAGA

Primers to add MiSeq adapters

forward primer	AATGATACGGCGACCACCGAGATCTACAC<u>XXXXXXXX</u>ACACTCTTTCCCTACACGACGCTCTT<br>"X" represents the plate barcode sequence
reverse primer	CAAGCAGAAGACGGCATACGAGAT<u>ZZZZZZZZ</u>GTGACTGGAGTTCAGACGTGTGCTCTTCCGATCT<br>"Z" represents the sample barcode sequence

Supplementary Methods

Construction of base-skipping linker libraries. Linker libraries were incorporated into the three host ZFPs (**Supplementary Figure 11**) via PCR with degenerate oligonucleotides utilizing an NNS randomization scheme. Oligonucleotides were purchased from Integrated DNA Technologies (IDT, Skokie, IL) and the linker length ranged from 2 to 12 residues. PCR with these oligonucleotides was performed on the amino-terminal two-finger module for the host ZFPs using Accuprime Pfx Polymerase (Thermo Fisher, Waltham, MA) and the corresponding carboxy-terminal two-finger modules for each host were amplified with standard primers. All primers contained BsaI restriction sites to generate complementary overhangs used for ligation of the modules and subsequent ligation into the phagemid vector. Sub-libraries for each linker length were amplified separately to avoid length bias during PCR. Following the initial PCR, the reactions were purified using the QIAGEN QIAquick PCR Purification Kit (QIAGEN, Hilden, Germany). The two-finger modules for each library length were then combined with the appropriate carboxy-terminal two-finger modules and digested for two hours with 500 units of BsaI-HF (NEB, Ipswich, MA). Digested amplicons were purified with QIAGEN MinElute PCR Purification Kit and the two fragments were ligated using T4 DNA ligase (Thermo Fisher). Ligated products were run on a 2% agarose gel and extracted using the QIAGEN QIAquick Gel Extraction Kit. Extracted products were then ligated into the phagemid vector pBluescript II SK(+) (Agilent Technologies, La Jolla, CA) with T4 DNA Ligase to fuse the assembled ZFP containing the linker library to gIII of M13K07 phage. These ligations were then purified by ethanol precipitation. A library pool was generated containing 3µl of each individual library, which was then combined with 300µl Invitrogen ElectroMAX DH12S cells (Thermo Fisher). The cells and library DNA were distributed into eight electroporation cuvettes (Bio-Rad, Hercules, CA) with 40 µl added to each cuvette. The cells were then electroporated using a Bio-Rad Gene Pulser II as per the manufacturer's protocol. All of the electroporations were pooled and recovered for one hour in 20ml of 2xYT at 37ºC with shaking at 250 rpm. A sample was taken at this point for plating on LB plates containing ampicillin to determine the number of transformants. The cells were then diluted into 400ml of 2xYT containing 100µg/ml ampicillin and 2% glucose and incubated for six hours at 37ºC at 250 rpm. The cells were then pelleted and resuspended in 2ml of 2xYT. A volume of 400µl was used to extract the DNA library using the QIAGEN HiSpeed Plasmid Maxi Kit.

Selection of base-skipping linkers using phage display. Selections were initiated by combining 4 µg of library DNA with 300 µl of XL1-Blue Electrocompetent cells (Agilent Technologies). Cells and DNA were transferred in 40 µl aliquots to electroporation cuvettes (Bio-Rad) and electroporated using a Bio-Rad Gene Pulser II as per the manufacturer's protocol. All of the electroporations were then pooled and recovered in 10 ml of 2xYT media in a 50 ml T-flask (Corning, Corning, NY) at 37ºC and 250 rpm. A sample was taken at this point to plate on ampicillin LB plates determine the number of transformants. Cells were infected with helper phage by adding 500 µl of M13K07 phage (Thermo Fisher) followed by incubation at 37ºC at 250 rpm for 1.5 hours in media containing 150 µg/ml ampicillin, 50 µg/ml kanamycin, and 10 µM $ZnCl_2$. Cells were then transferred to 8-well deepwell reservoir (E&K Scientific, Santa

Clara, CA) and 0.1 mM IPTG was added to induce expression of the ZFP-gIII fusion protein. The reservoir was incubated at 30ºC and 75 rpm for 24 hours to produce phage particles.

Following the overnight incubation, the reservoir was spun at 3283 x g in a centrifuge with a swing-bucket rotor to recover the phage particles. Binding reactions were set up in RNase-free striptubes (Thermo Fisher) in a binding buffer consisting of PBS (without magnesium or calcium) containing 1% Tween-20, 2% BSA, 10 µg/ml salmon sperm DNA, 10 µM $ZnCl_2$, and 1mM DTT. The binding reaction was started by the addition of 20 µl recovered phage particles and 0.2 pmol of appropriate target site for each host ZFP. The binding reaction was incubated at room temperature for one hour followed by transfer of the reaction to fresh RNase-free striptubes containing 50 µl of M280 Dynabeads (Thermo Fisher) for an additional 15 minutes at room temperature with slow rotation of the striptubes. Beads with bound phage were then washed three times with PBS containing 0.1% Tween-20 and 10 µM $ZnCl_2$. Beads were then further washed two times with PBS containing 10 µM $ZnCl_2$. The last wash was transferred to fresh striptubes before the removal of the buffer. After removal of the final wash buffer, 200 µl of K91 cells (OD_{600} 0.8-1.0) were added to the tubes and cells/beads mixture was transferred to a 96-well deepwell block (E&K Scientific). An additional 100 µl of K91 cells was added and the block was incubated at 37ºC and 250rpm for 30 minutes to infect the cells with the bound phage. An additional 200 µl of 2xYT was added and the block was incubated for a further 30 mintutes. The cells were then infected with helper phage by the addition of 20 µl M13K07 helper phage, 150 µg/ml ampicillin, and 10 µM $ZnCl_2$ followed by incubation for 1.5 hours at 37ºC and 250 rpm. Finally, 500 µl of 2xYT containing 150 µg/ml ampicillin, 50 µg/ml kanamycin, 10 µM $ZnCl_2$, and 0.1 mM IPTG was added prior to incubation at 30ºC and 250 rpm for 20 hours. Following this incubation, the deepwell block was spun at 3283 x g to recover the phage particles that would be used for the subsequent round of selection, starting from the binding reaction step. Starting in round 2, 1000x competitor target (**Supplementary Figure 12**) was added for each subsequent round.

Retention efficiencies were determined as follows. For the input phage, a 10-fold dilution series of phage was made in 96-well plates. Phage then infected K91 cells by addition of 50 µl of each dilution to 50 µl of K91 cells. The plate was incubated at 37ºC for one hour. Six replicates of 5 µl from each dilution were spotted onto LB plates containing ampicillin and incubated overnight at 30ºC to determine the number of infected cells. For the output phage, 10 µl were taken following the infection of K91 cells with the bound phage and added to a 96-well plate containing 100 µl of 2xYT media. A 10-fold dilution series was made of these cells. Six replicates of 5 µl from each dilution were spotted onto LB plates containing ampicillin and incubated overnight at 30ºC to determine the number of infected cells. Retention efficiencies were calculated for all selections and each round as (output phage/input phage * 100).

Supplementary References

1. Perez, E.E. et al. Establishment of HIV-1 resistance in CD4+ T cells by genome editing using zinc-finger nucleases. *Nat Biotechnol* **26**, 808-816 (2008).
2. Hockemeyer, D. et al. Efficient targeting of expressed and silent genes in human ESCs and iPSCs using zinc-finger nucleases. *Nat Biotechnol* **27**, 851-857 (2009).
3. Urnov, F.D. et al. Highly efficient endogenous human gene correction using designed zinc-finger nucleases. *Nature* **435**, 646-651 (2005).
4. Pavletich, N.P. & Pabo, C.O. Zinc finger-DNA recognition: crystal structure of a Zif268-DNA complex at 2.1 A. *Science* **252**, 809-817 (1991).
5. Kim, J.S. & Pabo, C.O. Getting a handhold on DNA: design of poly-zinc finger proteins with femtomolar dissociation constants. *Proc Natl Acad Sci U S A* **95**, 2812-2817 (1998).
6. Nomura, W. & Sugiura, Y. Effects of length and position of an extended linker on sequence-selective DNA recognition of zinc finger peptides. *Biochemistry* **42**, 14805-14813 (2003).
7. Miller, J.C. et al. Improved specificity of TALE-based genome editing using an expanded RVD repertoire. *Nat Methods* **12**, 465-471 (2015).
8. Gupta, A. et al. An optimized two-finger archive for ZFN-mediated gene targeting. *Nat Methods* **9**, 588-590 (2012).
9. Segal, D.J., Dreier, B., Beerli, R.R. & Barbas, C.F., 3rd Toward controlling gene expression at will: selection and design of zinc finger domains recognizing each of the 5'-GNN-3' DNA target sequences. *Proc Natl Acad Sci U S A* **96**, 2758-2763 (1999).
